# Uncovering new antibiofilm roseabols from marine *Clonostachys rosea* through structural and multiomics approaches

**DOI:** 10.1007/s13659-026-00659-3

**Published:** 2026-11-11

**Authors:** Gerardo Della Sala, Carmen Festa, Mattia Cammarota, Marcella Pasqualetti, Martina Braconcini, Donatella de Pascale, Valentina Catania, Donatella Ferraro, Domenico Schillaci, Simona De Marino, Massimiliano Fenice, Maria Valeria D’Auria

**Affiliations:** 1 Department of Ecosustainable Marine Biotechnology, Stazione Zoologica Anton Dohrn, Naples, Italy; 2 Department of Pharmacy, University of Naples “Federico II”, Naples, Italy; 3 Department of Biological and Ecological Sciences, University of Tuscia, Viterbo, Italy; 4 Department of Earth and Marine Sciences (DiSTeM), University of Palermo, Palermo, Italy; 5 Department of Health Promotion, Mother and Child Care, Internal Medicine and Medical Specialties (PROMISE), University of Palermo, Palermo, Italy

**Keywords:** Marine fungi, *Clonostachys rosea*, Molecular networking, Peptaibols, Antibiofilm activity, Nonribosomal peptide synthetase

## Abstract

Through a combined approach, that involved the one-strain-many-compounds (OSMAC) culturing approach, metabolomic and genomic studies, and bioassay-guided purification we disclose the marine fungus *Clonostachys rosea* as producer of a family of peptaibols with new structural features. HRMS/MS, NMR, and advanced Marfey’s analysis enabled the structural elucidation of two major compounds of the roseabol family, named roseabols B (**1**) and C (**2**). Molecular networking further revealed a highly diverse and microheterogeneous peptaibol profile. Biological evaluation highlighted the ability of the isolated peptaibols to inhibit biofilm formation against *Escherichia coli* and *Candida albicans*. Overall, these studies demonstrate the effectiveness of metabolomics-driven strategies in discovering novel natural products and identify marine-derived peptaibols as promising antibiofilm agents.

**Graphical abstract:**

**Figure:**
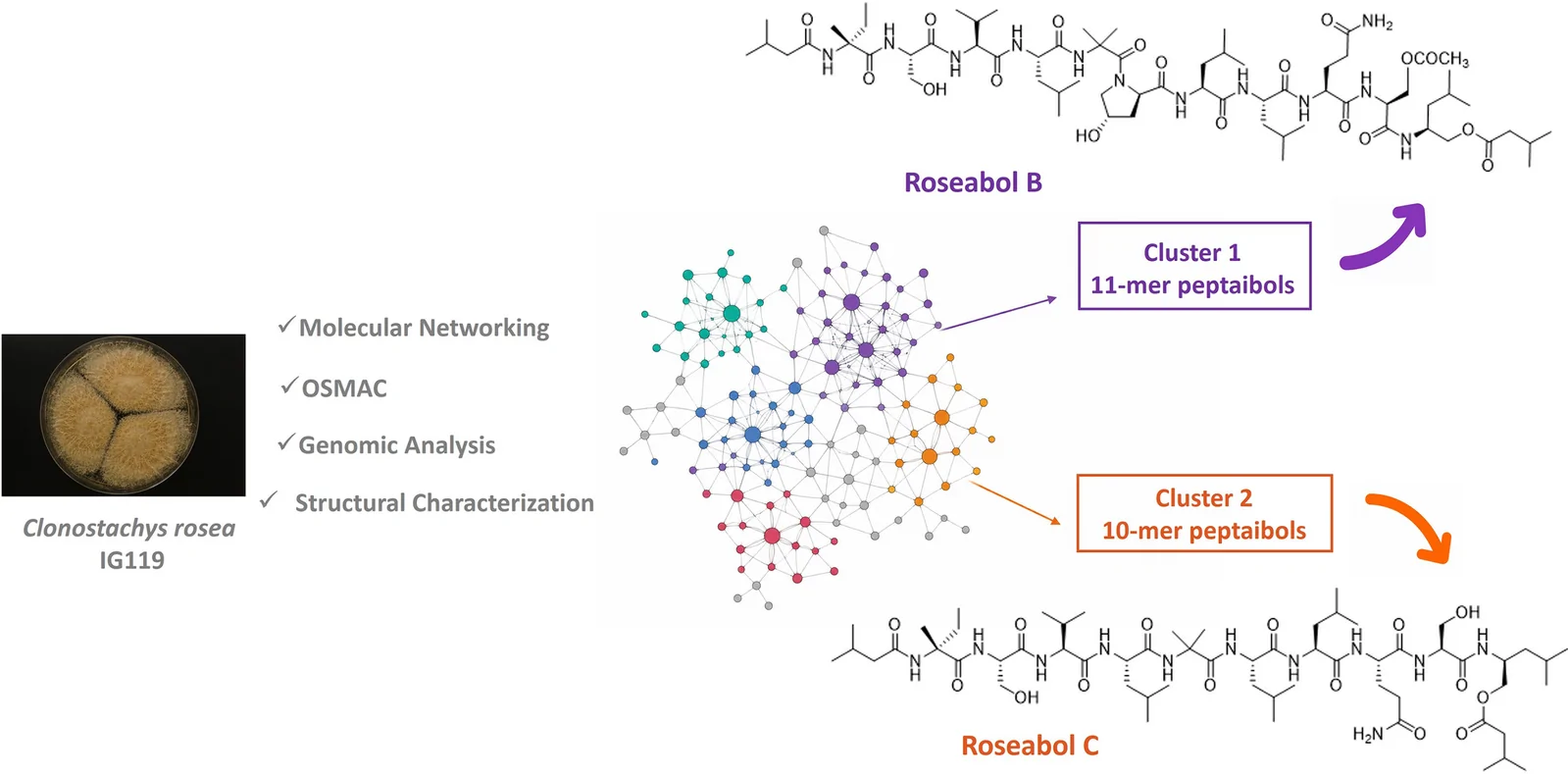

**Supplementary Information:**

The online version contains supplementary material available at https://doi.org/10.1007/s13659-026-00659-3.

## Introduction

Marine-derived fungi have been proven to be a relevant source of structurally unique and drug-like compounds [1]. However, the metabolome of these microorganisms appears very complex, and the production of certain compounds rather than others appears strongly influenced by cultivation conditions.

Recent advances in untargeted metabolomics, including analytical techniques and data analysis methods, have facilitated the use of mass spectrometry (MS)-based approaches for a fast and reliable analysis of crude extracts and identification of specific metabolites produced under varying culture conditions [2]. In this context the MS-based Global Natural Product Social Molecular Networking (GNPS) platform [3] enables the dereplication of complex extracts in a fast and high-throughput manner as well as the identification of novel chemical scaffolds and analogs of known substances and the determination of the best condition to produce specific secondary metabolites.

The ascomycete *Clonostachys rosea* (family Bionectriaceae) is cosmopolitan and inhabits several habitats, such as soils and plants [4]. Members of this species can colonize living plants and, as mycoparasites, they show antagonistic activities against fungal phytopathogens [5], nematodes [6], and insects [7]. This bioactivity could be ascribed to different mechanisms, the most relevant one is the production of hydrolytic enzymes, such as chitinases, glucanases and proteases, able to degrade cell-walls, but also the capability of inactivating mycotoxins through specific biotransformation and through the activation of defense enzymes and genes related to defense in plants [8]. Surely, the production of specialistic bioactive secondary metabolites represents one of the mechanisms used in the biocontrol activity, although there are relatively few reports on the isolation of secondary metabolites from this species [9–15].

There are few reports of *C. rosea* from marine environments and the compounds described include a rare unsaturated fatty acid [16], the triterpene eburicol [17], and the flavone chrysin [18], all of which have shown antiproliferative activity. Recently, our group reported the isolation of a new marine strain, *C. rosea* IG119, from the marine phanerogam *Posidonia oceanica* [19]. This strain can grow and produce chitinolytic enzymes under polyextremophilic conditions [20, 21].

As part of a project aimed at exploring the biotechnological potential of marine polyextremophilic fungal strains belonging to our in-house collection, we found that the extract of the previously characterized marine strain *C. rosea* IG119 [19] exhibited significant antimicrobial activity against selected ESKAPEE pathogens. This finding prompted further investigations into the secondary metabolite production of this strain. Untargeted metabolomic analysis combined with feature-based molecular networking (FBMN) [22] enabled the annotation of several metabolites belonging to the peptaibol family. Although peptaibols have previously been reported from a terrestrial *C. rosea* strain [13], the metabolomic profile of strain IG119 suggested the presence of a complex and largely unexplored peptaibol mixture. The application of the OSMAC (One Strain–Many Compounds) approach allowed us to optimize their production and facilitate the isolation of these metabolites for their structural and pharmacological characterization.

Peptaibols represent the largest family of fungal peptides and are characterized by the presence of non-proteinogenic amino acids, such as α-aminoisobutyric acid (Aib) and isovaline (Iva), together with proteinogenic residues including alanine, valine, leucine, and isoleucine. These peptides have attracted considerable attention because of their remarkable structural diversity and broad spectrum of biological activities [23–25], particularly antimicrobial and antifungal effects. More recently, increasing attention has been devoted to the discovery of natural products able to interfere with microbial biofilms, which are recognized as major contributors to persistent and chronic infections. In this context, the present study aimed to characterize the peptaibol metabolome of the marine strain *C. rosea* IG119 and to evaluate the antimicrobial and antibiofilm potential of the metabolites produced by this fungus.

## Results and discussion

### Selection of fermentation conditions

**Fig. 1:**
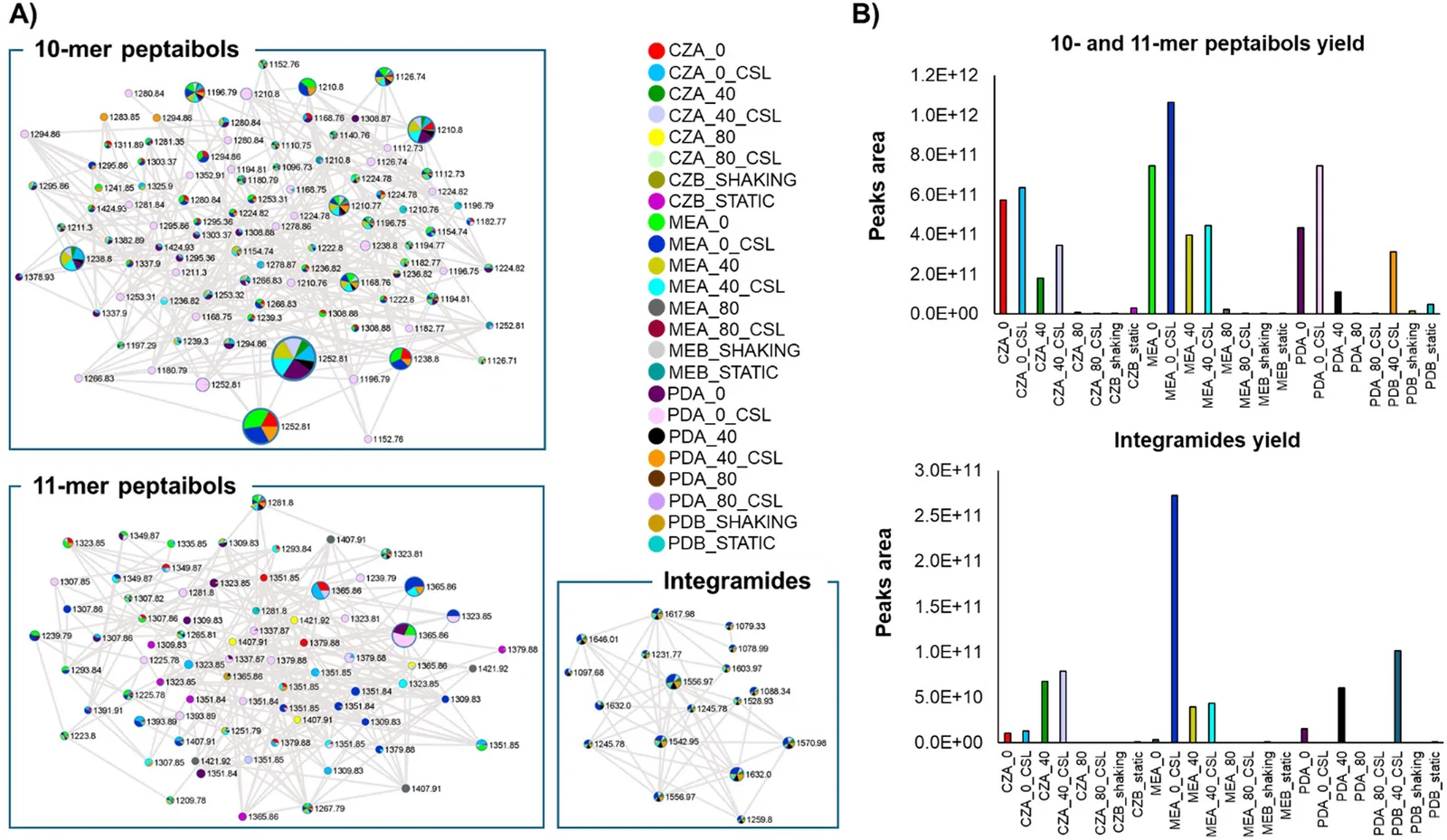
**A** 10- and 11-mer peptaibol and integramide molecular networks from crude extracts produced by *Clonostachys rosea* IG119 in 24 different culture conditions. Nodes are visualized as pie charts showing metabolite distribution in the 24 extracts. Node size is relative to the areas of the relevant peaks in the extracted-ion chromatograms from the LC–MS run. **B** Histograms report peak areas of peptaibols and integramides from the OSMAC conditions. Culture media abbreviations are as follows: CZA, Czapek-Dox Agar; CZB, Czapek-Dox Broth; MEA, Malt Extract Agar; MEB, Malt Extract Broth; PDA, Potato Dextrose Agar; PDB, Potato Dextrose Broth. CSL indicates supplementation with Corn Steep Liquor. Numbers associated with each condition refer to the salinity level (‰ sea salt)

In the frame of a research project aimed at discovering the biotechnological potential of polyextremophilic marine fungi, we found that the CHCl_3_ fraction of the small-scale EtOAc extract of the marine strain *C. rosea* IG119 grown on MEA medium displayed significant antibacterial activity against selected Gram-positive and Gram-negative bacterial pathogens, including *Staphylococcus aureus* ATCC 25923, *Pseudomonas aeruginosa* ATCC 15442, *Escherichia coli* ATCC 25922, together with a weak activity against *Enterococcus faecalis* ATCC 29212 and *Candida albicans* ATCC 10231 (Table S1). Therefore, this strain was selected for further chemical investigation to characterize its secondary metabolite profile and identify bioactive constituents. To gain insight into the metabolite composition, the extract was preliminarily analyzed by UPLC–UV–MS and molecular networking. This analysis revealed a cluster of high molecular weight nodes (*m*/*z* 1108–1365), whose manual inspection of the MS^2^ fragmentations suggested the presence of a family of undescribed 10-mer and 11-mer peptaibols. (Figure S1). Based on these findings, an OSMAC approach [26] was used to optimize culture conditions to target enhanced peptaibol production. Three different media, potato dextrose (PD), Czapek (CZ) and malt extract (ME), were tested under liquid (static and shaken) and solid conditions, at different salinity levels, and in the presence or absence of corn steep liquor (CSL), as a production elicitor [27], resulting in a total of 24 culture conditions (Fig. 1).

The resulting cultures were extracted with EtOAc and the crude extracts analyzed by high-resolution liquid chromatography coupled with tandem mass spectrometry (LC–HRMS/MS). A comparative metabolomic analysis was then performed through FBMN using the GNPS platform, thus generating a global molecular network where three molecular clusters, namely integramides, 11-mer peptaibols and 10-mer-peptaibols were identified (Fig. 1A).

Specifically, this analysis highlighted significant variations in the production of these metabolite families across different culture media. Comparative quantitative analysis of the metabolic profiles (Fig. 1B) reveals that solid ME medium supplemented with CSL (1 g/L) yielded the highest peptaibol production. In addition, this condition was also the only one able to induce appreciable integramide production. Accordingly, this medium was selected as the optimal condition for large-scale fermentation.

### MS-based molecular networking analysis of *Clonostachys rosea* IG119 peptaibiome

Preliminary LC/MS analysis, integrated with molecular networking (Figure S1), revealed that *C. rosea* IG119 produces a highly complex and microheterogeneous mixture of peptaibols and peptaibiotics, primarily occurring as minor components. These findings align with previous studies demonstrating the efficacy of untargeted MS-based approaches in expanding the known diversity of peptaibols [28, 29].

Following the large-scale fermentation of *C. rosea* in ME medium, supplemented with CSL, the methanol-soluble fraction of the crude EtOAc extract was prepared at 1 mg/mL in LC–MS grade methanol. Samples were analyzed via LC–HRMS/MS utilizing data-dependent acquisition (DDA) in positive ionization mode. Raw data were preprocessed using MZmine 2.53 and exported to the GNPS platform for FBMN analysis [22].

By organizing metabolites into clusters based on MS/MS fragmentation similarity, FBMN enables the visualization of structurally related compounds and the annotation of known metabolites through spectral library matching. Through careful optimization of chromatographic and data-processing parameters, the resulting molecular network resolved the three distinct clusters corresponding to integramides, 11-mer and 10-mer peptaibols produced under the selected growth condition, as depicted in Figure S2 and Tables S2–S4. These clusters were subsequently investigated for MS-based structural prediction, as detailed below.

### Integramides molecular cluster

The integramide cluster, in which the node at *m*/*z* 1632.0019 [M + H]⁺ was automatically annotated as integramide A (**3**) by GNPS, was not observed in the preliminary analysis of the small-scale extract, since the production of integramide A and its congeners seems to be strongly influenced by the culture conditions. Detailed examination of the MS^2^ fragmentation patterns confirmed the presence of integramide A and suggested several related analogues (Figure S2; Table S2). The ESI MS/MS fragmentation of the peak at *m*/*z* 1632.0019 was in accordance with the primary structure of integramide A (**3**) and with reported MS^2^ experimental values. An isobaric species (R_t_ 12.0 min) showed a fragmentation pattern compatible with Iva_4_ → Aib_4_ and Aib_8_ → Val/Iva_8_ substitutions with respect to integramide A. It represents an undescribed variant and was named as integramide C (**5**). Manual inspection of the MS^2^ fragmentation pattern suggested the identity of peak at *m*/*z* 1646.0180 as integramide B (**4**), whereas the two new species at *m*/*z* 1603.9694 and 1617.9854, designated as integramides D (**6**) and E (**7**), were two positional variants of integramide A (**3**), with the former replacing Iva_1_ and Iva_4_ with two Aib residues and the latter featuring Iva_4_ → Aib_4_ substitution.

The observed structural variability within members of this small family likely reflects the relaxed selectivity of the adenylation domains of the nonribosomal peptide synthetase (NRPS) involved in the biosynthesis of these peptides, a common feature within peptaibol synthetases [25].

Integramides A and B belong to the family of peptaibiotics, that share with peptaibols the high percentage of α-dialkylated amino acids but differ for the presence of a terminal amino acid instead of a terminal amino alcohol. They were isolated from the fungal extracts of *Dendrodochium* sp. and, interestingly, displayed inhibitory activity against HIV-integrase [30]. After their isolation, De Zotti et al. [31] reported the total synthesis of integramide A, solving some stereochemical ambiguities, and the synthesis of some modified analogues that retain the anti-viral activity [32].

Unfortunately, integramides were detected as minor components co-eluting with major 10- and 11-mer peptaibols, and we were unable to perform their isolation and NMR-based structural characterization.

### 11-mer roseabols molecular cluster

The 11-mer peptaibol cluster was the most populated. The major node at *m*/*z* 1365.8629 corresponded to roseabol B (**1**), whose structure was used as a reference for the annotation of related congeners.

Comprehensive manual dereplication of each node based on HRMS and MS/MS data allowed the identification of several analogues (Figure S2; Table S3). Overall, mass fragmentation spectra of these 11-mer peptaibols are characterized by a series of *b*_10_–*b*_1_ ions accompanied by *y*_10_–*y*_6_ and *y*_4_–*y*_3_ ions of lower intensity, which facilitate full sequence of assignment. Notably, product ion spectra are dominated by the *b*_5_ and *y*_6_ fragment ions, arising from the preferential cleavage at Aib_5_–Hyp/Pro_6_ peptide bond.

Structural diversification of the 11-mer peptaibols’ backbone is primarily driven by amino acid substitutions at positions 1 (Aib/Iva), 3 (Val/Aib or Leu/Ile), 5 (Aib/Iva), and 6 (Hyp/Pro). To a lesser extent, variability is also observed at positions 2 (Ser/Ala), 4 (Leu/Aib/MetO), 9 (Gln/Glu or Glu-OMe), and 10 (Ser/Ala or AcLys), while the Leu_7_–Leu_8_ dipeptide motif remains strictly conserved. Such sequence heterogeneity is characteristic of peptaibols and reflects the broad substrate specificity of the NRPS adenylation domains responsible for their biosynthesis.

Furthermore, these 11-mer peptaibols exhibit a high degree of chemical diversity due to the presence of up to four acyl groups at specific backbone sites. Notably, roseabol congeners display a distinct acylation pattern, including acetylation, isovalerylation, succinylation, and/or propionylation. These modifications occur at Iva/Aib_1_ α-amino group, at the side‐chain hydroxy groups of Ser_10_, Hyp_6_, and Ser_2_, and at the terminal hydroxy groups of Leuol_11_. The substitution patterns of roseabols were inferred through the identification of characteristic neutral losses—specifically as ketenes or carboxylic acids—from the corresponding fragment ions.

Based on these observations and assuming a shared biosynthetic origin from the same NRPS cluster, residues at position 3, 4, 6–11 were assigned by analogy with roseabol B (**1**) (characterized by NMR in Sect. 2.6), although MS data do not allow discrimination between isobaric amino acids, such as leucine and isoleucine. Similarly, the presence of isovaleric acid residues acylating the N-and C-termini was assumed across roseabol congeners.

### 10-mer roseabols molecular cluster

The 10-mer peptaibol cluster was annotated following a similar workflow (Figure S2; Table S4). These compounds are closely related to the 11-mer congeners but lack the sixth amino acid residue, typically Hyp or Pro.

This observation suggests that module 6 of the NRPS synthetase might be skipped for the generation of the shorter chain congeners, a phenomenon already reported, and demonstrated by genome analysis, to produce peptaibols.

By analogy with the parent structure of roseabol C (**2**) (characterized by NMR in Sect. 2.6), we tentatively discriminated between the isobaric Ile/Leu, Ileuol/Leuol, and Val/Iva residues, thus assigning Leu at positions 4, 6, and 7, Val at position 3, and Leuol as the C-teminal amino alcohol, where applicable (Table S4).

### Isolation and structure elucidation of roseabols B (1) and C (2)

**Fig. 2:**
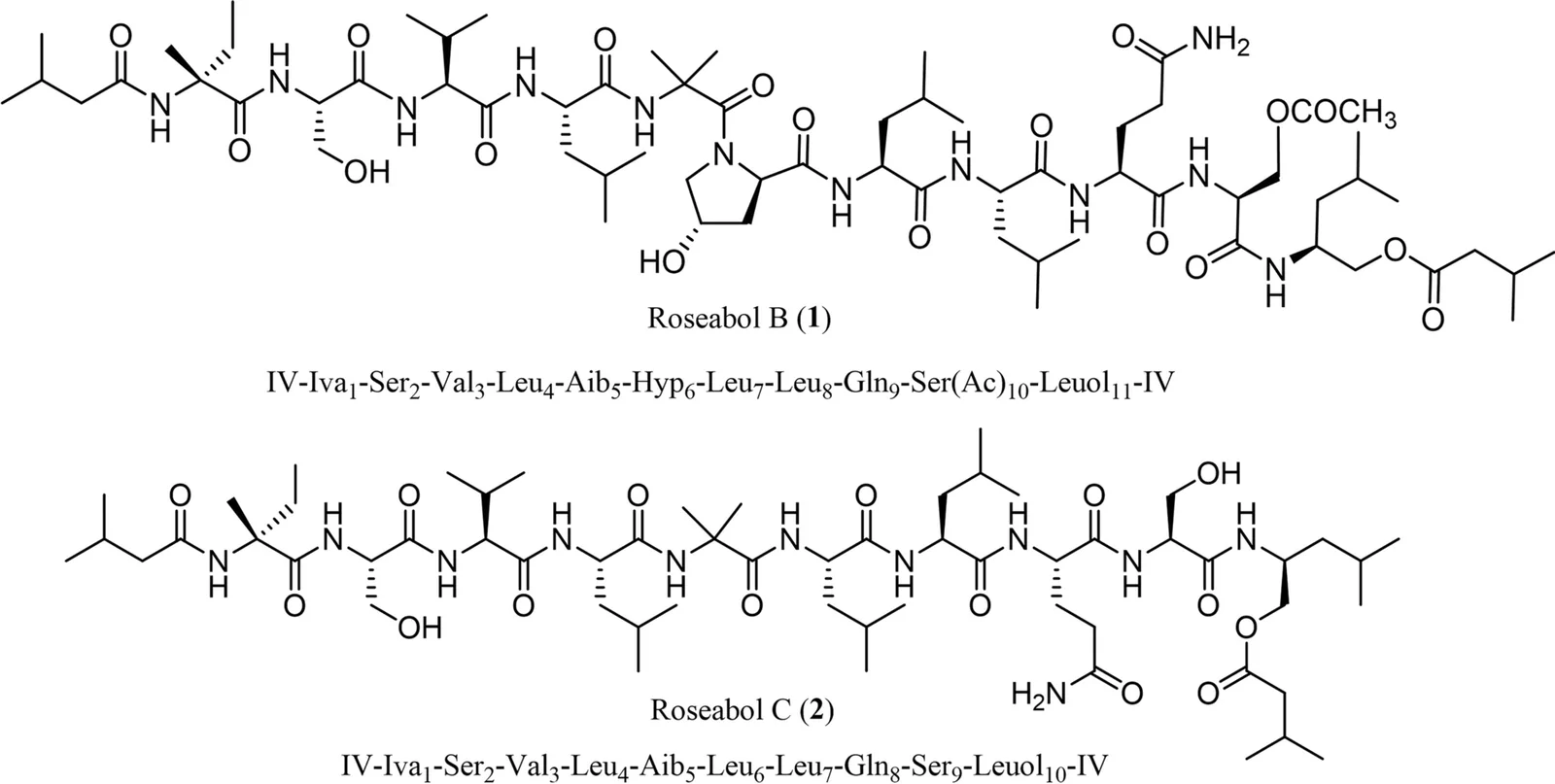
Structures of roseabols B (**1**) and C (**2**), new peptaibols isolated from the marine strain *Clonostachys rosea*. IV = Isovaleryl unit; Leuol = leucinol unit

As revealed by MN analysis, the methanolic extract is a highly complex, microheterogeneous mixture of peptaibols. To isolate the major components, reversed-phase MPLC followed by repeated HPLC runs was performed on the methanolic extract, affording two major peptaibols, named roseabols B (**1**) and C (**2**) (Fig. 2).

#### Structural characterization of roseabol B (1)

The molecular formula of roseabol B (**1**) was established as C_66_H_116_N_12_O_18_ based on HRESIMS data (*m*/*z* 1365.8629 [M + H]^+^), in association with the NMR analysis.

**Fig. 3:**
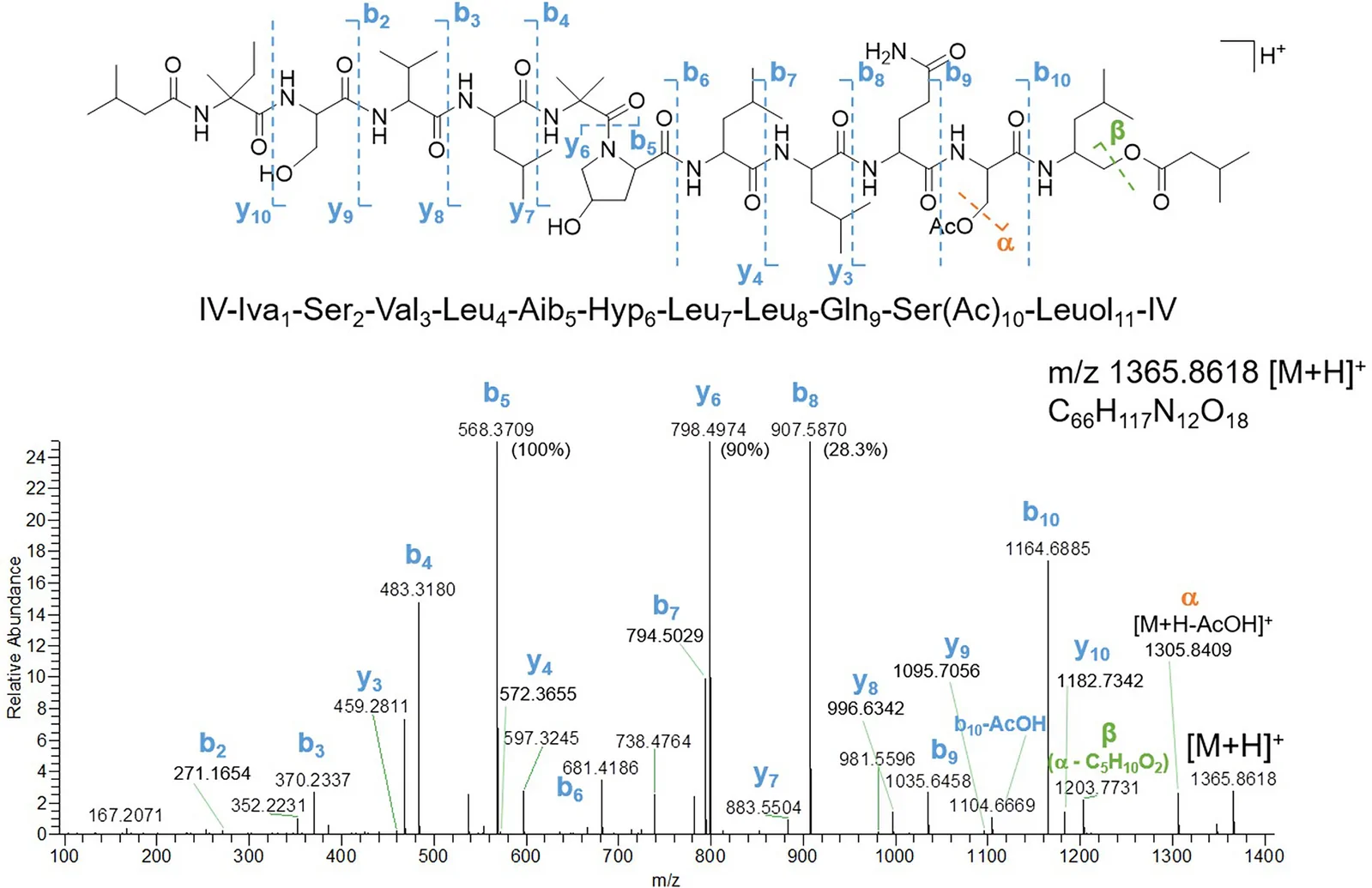
MS/MS spectrum of roseabol B (**1**)

The MS/MS spectrum (Fig. 3) showed two fragment ions at *m*/*z* 1305.8409 and 1203.7731 arising from the b-elimination of an acetic acid moiety and a C5 carboxy residue, respectively, supporting the presence of two C2 and C5 acyl substituents. In addition, as already reported for peptaibol derivatives [25] mostly b-series ions are observed, which comprise ions from *b*_10_ to *b*_2_, corresponding to sequential cleavage from the C-terminus toward the N-terminus.

In the C-terminal portion, the fragment at *m*/*z* 1164.6885 (*b*_10_) was indicative of a C-terminal C5 acylated-leucinol/isoleucinol (Lxxol) residue. The subsequent losses of acetic acid and serine (*b*_9_) were consistent with an Ac-Ser motif. Further characteristic losses of amino acid residues and comparative analysis using y-type fragments, indicated the sequence C_4_H_9_CO-Vxx_1_-Ser_2_-Vxx_3_-Lxx_4_-Aib_5_-Hyp_6_-Lxx_7_-Lxx_8_-Gln_9_-Ser(Ac)_10_-Lxxol_11_(COC_4_H_9_) (Fig. 3).

With the aid of preliminary information obtained by mass spectrometry analysis, NMR investigation allowed us to solve the structural ambiguities left by MS, such as the actual identity of isobaric amino acid residues (Leu/Ile, Leuol/Ileol, Val/Iva) and the linear or branched nature of the C5 acyl units at both N- and C-termini. Furthermore, NMR analysis enabled the assignment of all NMR chemical shifts.

The planar structure was elucidated by comprehensive NMR spectroscopic analysis, including one- and two-dimensional NMR experiments (^1^H, ^13^C, HSQC, TOCSY, COSY, HMBC, and 2D HSQC-TOCSY). NMR experiments were acquired in both acetone-d_6_ (Table S5) and in DMSO-d₆ (Table 1), solvents that allows the visualization of the exchangeable protons. The data obtained in DMSO-d_6_ are discussed in the main text, as this solvent provided better signal dispersion.

**Table 1:**
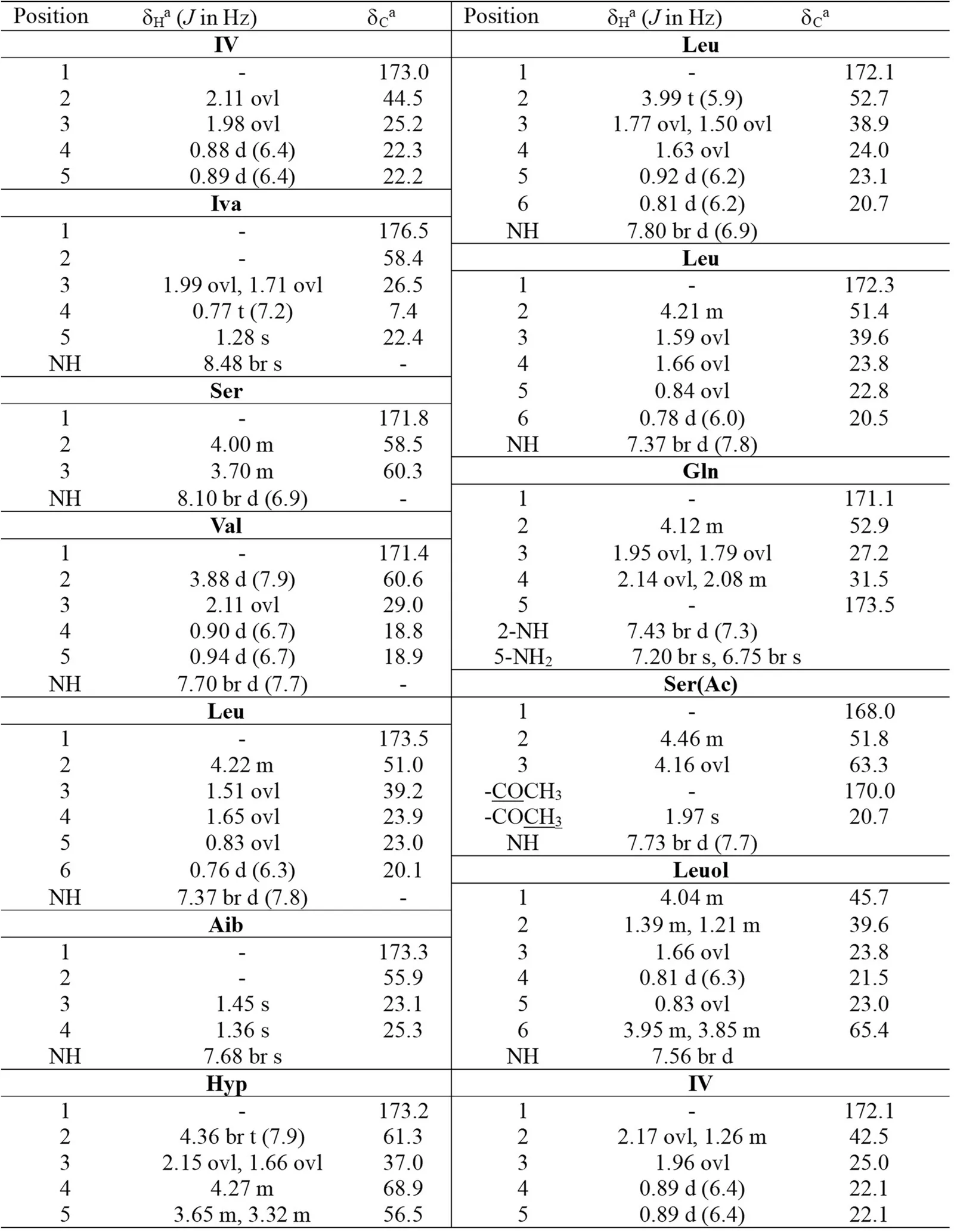
^1^H and ^13^C NMR data (700 and 175 MHz, DMSO-d_6_) of roseabol B (**1**)

^a1^H and ^13^C assignments aided by 2D COSY, TOCSY, HSQC, HMBC and 2D HSQC-TOCSY experiments

The ^1^H- and ^13^C-NMR spectra of roseabol B (**1**) displayed signals attributable to ten amide NH protons (*δ*_H_ 8.48–7.37) and fourteen carbonyl carbons (*δ*_C_ 176.5–168.0), respectively, consistent with the presence of a peptide structure.

**Fig. 4:**
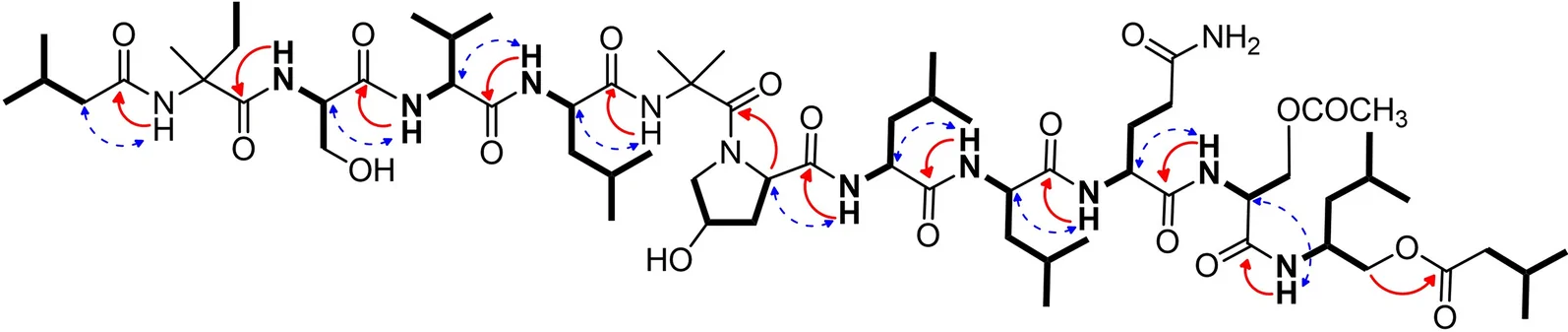
Selected HMBC correlations (red arrows), NOESY correlations (blue dashed arrows), COSY/TOCSY connectivities (in bold) observed for roseabol B (**1**), supporting the assignment of the amino acid sequence and confirming the linear connectivity of all residues

The 2D HSQC-TOCSY experiment was highly diagnostic for this analysis, enabling simultaneous tracing of individual amino acid spin systems and assignment of ^13^C chemical shifts by identifying all protons associated with each carbon spin system. The comprehensive correlations observed in the 2D HSQC-TOCSY spectrum not only facilitated the assignment of ^13^C chemical shifts but also provided crucial information on the connectivity and spin networks of the peptide backbone. Using this approach, and through careful interpretation of COSY and HMBC data, we were able to identify two serine residues [Ser(Ac)_10_ and Ser_2_], one isovaline (Iva_1_), one valine (Val_3_), one hydroxyproline (Hyp_6_), one glutamine (Gln_9_), three leucines (Leu_4_, Leu_7_ and Leu_8_), one α-aminoisobutyric acid (Aib_5_) and a C-terminal leucinol residue (Leuol_11_) together with one acetyl and two isovaleryl groups. The presence of the C-terminal isovaleryl-leucinol_11_ was confirmed by the combined analysis of COSY and TOCSY experiments (Fig. 4). In particular, the isovaleryl moiety was identified through characteristic COSY correlations between H_2_−2 and H-3, which in turn exhibited further spin–spin couplings with the methyl protons in the TOCSY spectrum. A similar analysis allowed the assignment of a leucinol moiety. The downfield shift of the two methylene carbinol protons of leucinol residue were indicative of an acylation shift and, consistently, in the HMBC experiment, a clear cross-peak was observed between H_2_−6 of Leuol_11_ (*δ*_H_ 3.95 m, 3.85 m) and C-1 (*δ*_C_ 172.1) of the isovaleric acid residue, confirming the ester linkage at the C-terminus. To our knowledge, this represents the first reported peptaibol structure bearing an isovaleryl-acylated C terminus, whereas two peptaibols with an acetylated C terminus have previously been described [33].

The combined HMBC (Fig. 4) and NOESY data unambiguously established the amino acid sequence and connectivity of all residues in roseabol B (**1**). In detail, the correlations between NH Iva_1_ (*δ*_H_ 8.48)/C1 IV (*δ*_C_ 173.0), NH Ser_2_ (*δ*_H_ 8.10)/C1 Iva_1_ (*δ*_C_ 176.5), NH Val_3_ (*δ*_H_ 7.70)/C1 Ser_2_ (*δ*_C_ 171.8), NH Leu_4_ (*δ*_H_ 7.37)/C1 Val_3_ (*δ*_C_ 171.4), NH Aib_5_ (*δ*_H_ 7.68)/C1 Leu_4_ (*δ*_C_ 173.5), H-2 Hyp_6_ (*δ*_H_ 4.36)/C1 Aib_5_ (*δ*_C_ 173.3), NH Leu_7_ (*δ*_H_ 7.80)/C1 Hyp_6_ (*δ*_C_ 173.2), NH Leu_8_ (*δ*_H_ 7.37)/C1 Leu_7_ (*δ*_C_ 172.1), NH Gln_9_ (*δ*_H_ 7.43)/C1 Leu_8_ (*δ*_C_ 172.3), NH Ser_10_ (*δ*_H_ 7.73)/C1 Gln_9_ (*δ*_C_ 171.1), NH Leuol_11_ (*δ*_H_ 7.56)/C1 Ser_10_ (*δ*_C_ 168.0), CH_2_ Leuol_11_ (*δ*_H_ 3.85, 3.95)/C1 IV (*δ*_C_ 172.1) confirmed the linear sequence.

The absolute configuration of the Iva residue was inferred from the chemical shifts of its ethyl moiety. In agreement with literature data, where (*R*)-Iva residues are characterized by more shielded γ-CH_3_ protons (*δ*_H_ < 0.89 ppm) and larger Δ*δ* values (>0.28 ppm) between the β-methylene protons [34], the experimental data support this assignment. In particular, the γ-CH_3_ signal (*δ*_H_ 0.77, <0.89 ppm) and the β-CH_2_ signal (*δ*_C_ 26.5, <30 ppm), together with the Δ*δ*_Hβ_–_Hα_ value (0.26 ppm) (Table 1), are consistent with an *R* configuration for the Iva residue.

The absolute stereochemistry of all the amino acid residues was determined by acid hydrolysis of roseabol B (**1**) (6 N HCl, 110 °C, 12 h) followed by advanced Marfey’s analysis [35–37]. The acid hydrolysate was derivatized with 1-fluoro-2,4-dinitrophenyl-5-l-alaninamide (l-FDAA), and LC–MS comparison of the derivatives from the parent compound with those of appropriate standards established the presence of (2*S*,4*R*)-Hyp, l-Ser, l-Val, d-Iva, l-Leuol, and l-Leu. Notably, l-Ser(Ac) remained intact after hydrolysis and, despite being isobaric with l-Glu, was unequivocally distinguished by its characteristic MS/MS fragmentation pattern (Figure S3).

The lipopeptaibol framework of roseabol B is akin to that of roseabol A, a peptaibol isolated from a soil-derived *C. rosea* with a very similar aminoacyl sequence. Compared to roseabol A [13], roseabol B (**1**) differs in its amino acid composition. Specifically, Val_4_ and Ser_10_ present in roseabol A are replaced in **1** by Leu_4_ and an acetylated Ser_10_, respectively. In addition, the C-terminal Leuol_11_ is acylated in compound **1** with isovaleric acid. Despite these substitutions, the overall peptaibol framework, sequence length, structural scaffold and the stereochemistry of α-aminoacyl residues remain conserved, suggesting a common biosynthetic origin.

#### Structural characterization of roseabol C (2)

The molecular formula of roseabol C (**2**) (Fig. 1) was determined as C_59_H_107_N_11_O_15_ based on HRESIMS analysis (*m*/*z* 1210.8039 [M + H]^+^). Interpretation of 1D and 2D NMR spectra, together with the selected NMR assignments summarized in Table S6, revealed that roseabol C (**2**) shares a highly similar structure and primary sequence with roseabol B (**1**). The absence of the characteristic Hyp and acetyl signals in the ^1^H NMR spectrum is consistent with a mass difference of 155 uma, supporting the absence of Hyp and the presence of a non-acetylated serine residue compared to compound **1**. Due to extensive signal overlap, particularly in the high-field region corresponding to the three Leu residues and the two isovaleryl units, only selected and unambiguously assigned NH, Hα and Cα resonances are reported in Table S6, whereas the remaining signals could not be unequivocally assigned. HMBC correlations, together with key MS^2^ analyses, enabled the complete amino acid sequence of roseabol C (**2**) to be established as IV-Iva_1_-Ser_2_-Val_3_-Leu_4_-Aib_5_-Leu_6_-Leu_7_-Gln_8_-Ser_9_-Leuol_10_-IV. The absolute stereochemistry of roseabol C (**2**) was assumed to be the same of roseabol B (**1**), based on their common biogenetic origin as detailed in Sect. 2.7.

#### Circular dichroism analysis of roseabols B (1) and C (2)

**Fig. 5:**
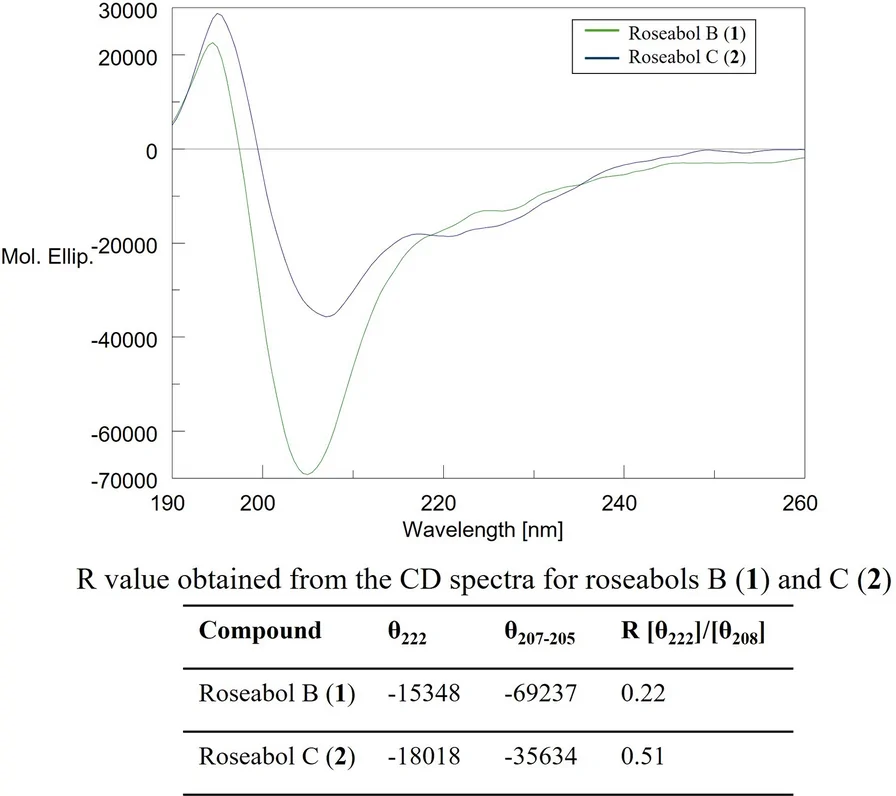
Circular dichroism (CD) profiles of roseabol B (**1**, line green) and C (**2**, line blue) in methanol

To complement their structural characterization and compare their conformational preferences in solution, the CD spectra of roseabols B (**1**) and C (**2**) were recorded in methanol (Fig. 5). The shapes of the CD pattern showed a −ive maxima at 205 and 207 nm respectively, and a −ive shoulder at 222 nm. The ellipticity ratio *R* [*θ*_*T*_^222^]/[*θ*_*T*_^205^] was found to be 0.22 for roseabol B and *R* [*θ*_*T*_^222^]/[*θ*_*T*_^207^] was found to be 0.51 for roseabol C. This clearly indicated that the compound **1** folds in a 3_10_-helix in methanol as the value for R was less than 0.50, which is known for the high propensity of 3_10_-helix while ratio R value for compound **2** points towards a prevalence of α-helix over 3_10_-helix [38, 39].

### Biosynthesis of roseabols from *C. rosea* IG119

**Fig. 6:**
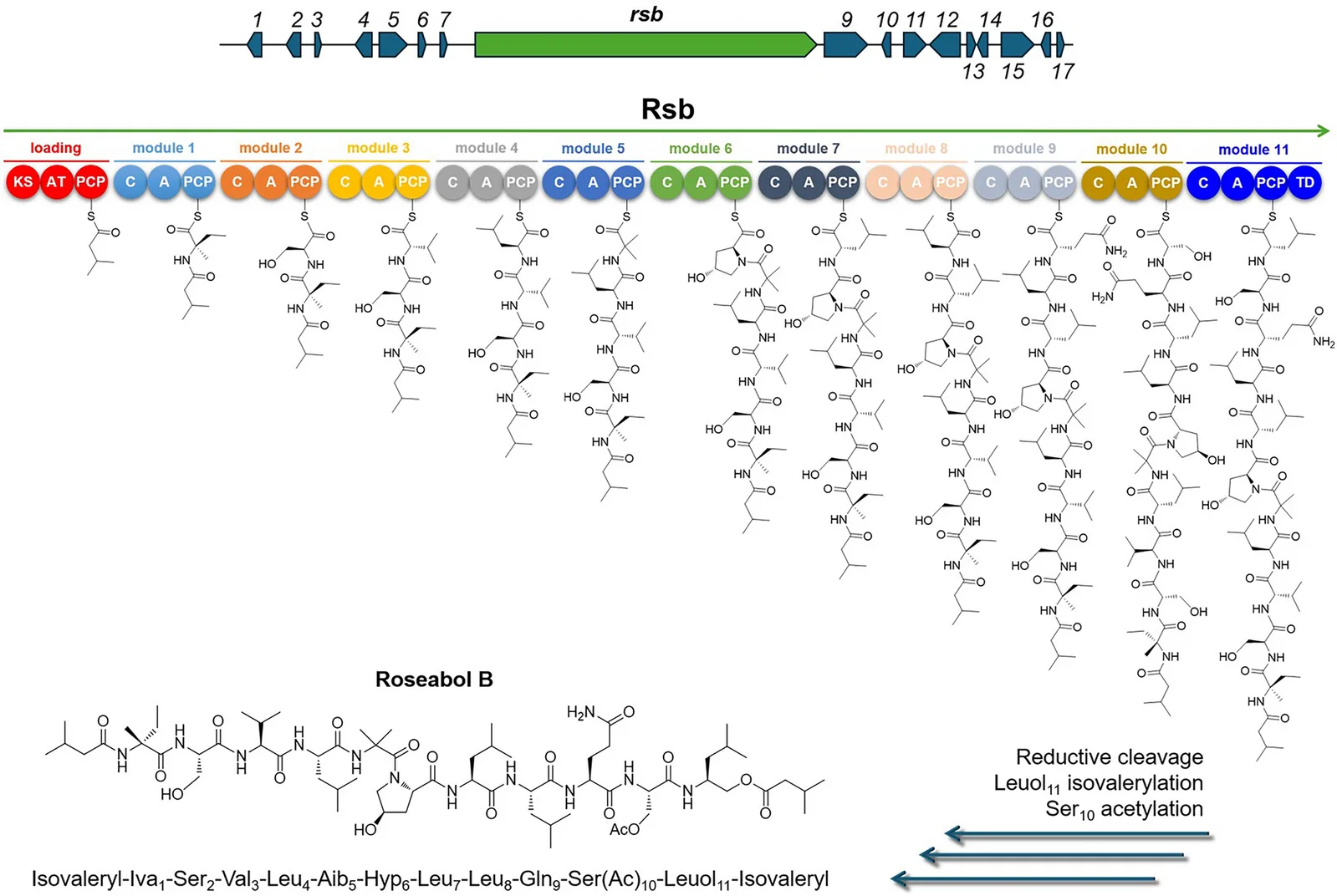
Putative biosynthesis of roseabol B (**1**)

To elucidate the biosynthetic pathway of roseabols from *C. rosea* IG119, the fungal genome was sequenced de novo and mined using antiSMASH [40] for NRPS biosynthetic gene clusters (BGCs), which are known to assemble peptaibols in other fungal species [41]. AntiSMASH identified 38 NRPS-related pathways, comprising 28 pure NRPS and 10 NRPS/PKS (polyketide synthase) hybrid BGCs. Roseabol biosynthesis was predicted to be governed by a 12-module NRPS/PKS assembly line—designated as *rsb* (Fig. 6; Table S7)—based on its modular architecture and the collinearity between the predicted specificity of 9 out of 11 adenylation (A) domains (modules 1–5 and 8–11, Table S8) and the primary amino acid sequence of the 11-mer peptaibols elucidated in Sects. 2.4 and 2.6.1 via NMR and tandem mass spectrometry.

The putative peptaibol BGC spans 40,059 bp, initiated by a loading PKS module (KS-AT-PCP) that catalyzes the N-acylation of Iva/Aib_1_. The nascent peptide chain is sequentially extended by modules 2–11, each featuring a C-A-PCP tridomain architecture responsible for adding a single amino acid, as predicted in silico using PARAS (Table S8) [42]. The terminal module recruits Leu_11_ and concludes with a thioester reductase (TD) domain, which facilitates reductive off-loading to yield a primary alcohol (Fig. 6). This is consistent with the terminal leucinol residue identified in all roseabol variants from *C. rosea* IG119.

Notably, the detection of 10-mer peptaibol congeners indicates that the Rsb synthetase can deviate from the canonical collinearity rule. Consistent with specific fungal and bacterial NRPS systems [30, 43, 44], a programmed module 6 skipping event likely occurs during biosynthesis to generate 10-mer peptaibols, which lack the Hyp/Pro_6_ residue found in the 11-mer congeners.

The genome-predicted configuration was congruent with the stereochemistry inferred by Marfey’s method for roseabol B. As the Rsb synthetase lacks epimerization domains, it primarily incorporates l-amino acids. The presence of d-Iva is consistent with the direct selection of the d-configured non-proteinogenic amino acid by the first NRPS module. Accordingly, the *C. rosea* IG119 genome contains homologues of TqaL, TqaF, and TqaM, which generate L/d-Iva and Aib from l-Leu, l-*allo*-Ile, and l-Val via hydrolysis and oxidative decarboxylation of an aziridine intermediate [45, 46]. Finally, while the relaxed substrate selectivity of the Rsb A domains, module skipping and substrate availability explain the chemical diversity of roseabol variants, the biosynthetic basis for the acylation of Ser_2_, Hyp_6_, Ser_10_, and Leuol_11_ observed in several congeners remains to be elucidated.

As well, although antiSMASH analysis of the fungal genome identified several multimodular NRPS biosynthetic BGCs, none could be definitively correlated with integramide biosynthesis in silico.

### Antimicrobial and antibiofilm activity of roseabols B (1) and C (2)

Since the chloroform extract had previously exhibited significant antibacterial activity, roseabols B (**1**) and C (**2**) were evaluated against the same microbial panel of bacterial and fungal pathogens. Neither compound showed inhibitory activity against planktonic microbial cells at the highest concentration tested (50 μg/mL), indicating MIC values above this concentration. The two compounds were subsequently evaluated for their ability to inhibit biofilm formation (Table 2).

**Table 2:** Inhibition of biofilm formation expressed as percentage of inhibition (%)

	50 µg/mL	25 µg/mL	12.5 µg/mL	6.2 µg/mL
Roseabol B (**1**)				
*S. aureus* ATCC 25923	n.s	n.s	n.s	n.s
*P.aeruginosa* ATCC 15442	n.s	n.s	n.s	n.s
*E. faecalis* ATCC 29212	n.s	n.s	n.s	n.s
*E.coli* ATCC 25922	34	26	n.s	n.s
*C.albicans* ATCC 10231	69	65	64	47
Roseabol C (**2**)				
*S. aureus* ATCC 25923	n.s	n.s	n.s	n.s
*P.aeruginosa* ATCC 15442	n.s	n.s	n.s	n.s
*E. faecalis* ATCC 29212	n.s	n.s	n.s	n.s
*E.coli* ATCC 25922	62	50	37	16
*C.albicans* ATCC 10231	42	41	38	36

n.s. = not significant, less than 10% inhibition

Interestingly, both roseabols displayed selective antibiofilm activity against specific microbial targets. Roseabol B (**1**) selectively inhibited biofilm formation by *C. albicans* ATCC 10231, showing a BIC₅₀ value of 7.25 μg/mL, while roseabol C (**2**) was particularly active against *E. coli* ATCC 25922, displaying a BIC₅₀ value of 25 μg/mL (Table 3) and achieving up to 50% inhibition of biofilm formation. The relatively short size of roseabols B (**1**) and C (**2**), 11-mer and 10-mer peptaibols respectively, may contribute to their limited effect on microbial agents [47]. Conversely, their selective inhibition of biofilm formation suggests a possible interference with processes involved in biofilm establishment rather than direct effects on microbial viability. Following emericellipsin A [48], roseabols B and C represent the second group of short peptaibols reported to exhibit antibiofilm activity. While large peptaibols (>15-mer) can form stable helical structures within the membrane and create ion channels—thereby causing membrane disruption—the putative antibacterial mechanism of short peptaibols may involve cell membranes and/or other intracellular targets [49]. Nevertheless, roseabols A and B have been demonstrated to adopt helical structures in solution. This is consistent with emericellipsin A and leucinostatin A, for which such a conformational propensity was deemed crucial for promoting membrane destabilization [49, 50]. This structural feature, coupled with their highly hydrophobic nature, could contribute to the observed roseabol antibiolfim activity. Although further studies are required to fully elucidate their mechanism of action, these results identify roseabols B (**1**) and C (**2**) as promising peptaibol-derived scaffolds for the development of novel antibiofilm agents.

**Table 3:** Inhibition of biofilm formation expressed as BIC_50_ (μg/mL)

Roseabol B (**1**)	*C. albicans* ATCC 10231 7.25 µg/mL
Roseabol C (**2**)	*E. coli* ATCC 25922 25 µg/mL

## Experimental

### Strains and culture conditions

The fungal strain IG119 was obtained from the culture collection of the “Laboratory of Ecology of Marine Fungi” (Department of Ecological and Biological Sciences, University of Tuscia, Viterbo, Italy). The strain isolated from the marine phanerogam *Posidonia oceanica* was identified as *C. rosea* in a previous work through a polyphasic approach, combining morphological, physiological and molecular methods [19] and cryogenically maintained at −40 °C. The strain has been revitalized and sub-cultured on Potato Dextrose Agar Seawater (PDAs: 39 g PDA dissolved in 1 L of filtered seawater) supplemented with antibiotics (streptomycin sulfate, 0.2 g/L; penicillin G 0.07 g/L; chloramphenicol, 0.05 g/L) at 23 °C. All media and components were purchased from Sigma-Aldrich (St. Louis, MO, USA), unless otherwise stated.

### Fermentation and extraction procedures

Strain IG119 was cultivated on Malt Extract Agar seawater (MEAs: 50 g MEA dissolved in 1 L of filtered seawater); eighty Petri dishes (9 cm diameter), were prepared. Fifty plates were inoculated at three points with 2 µL per point of a saline conidial suspension (0.5–1 × 10^5^ cell/mL) and incubated at 23 °C for 30 days. Subsequently, the fungal cultures were cut into small pieces (0.5 × 0. mm^2^) and extracted overnight with MeOH. The mycelium was then removed by filtration, and the organic phase was concentrated to dryness using a rotary evaporator (STRIKE300 Steroglass, Italy). The thirty uninoculated plates for each medium were similarly processed, and the extract was used as control.

The methanolic extracts of IG119 from MEAs media (17.0 g) was further subjected to a preliminary repartitioning procedure according to Kupchan [51]. The crude extract was dissolved in MeOH:H_2_O (7:3, *v*/*v*) and partitioned with CHCl_3_. After removal of methanol, the aqueous phase was further partitioned with *n*-BuOH. This procedure furnished three extracts: the CHCl_3_ extracts (693 mg), the *n*-BuOH extract (326 mg), and the aqueous residue.

### Determination of minimum inhibitory concentrations (MICs)

The following reference microbial strains were used: the Gram-positive bacteria *Staphylococcus aureus* ATCC 25923 and *Enterococcus faecalis* ATCC 29212; the Gram-negative bacteria *Pseudomonas aeruginosa* ATCC 15442 and *Escherichia coli* ATCC 25922; and the yeast *Candida albicans* ATCC 10231.

MICs were assessed by microdilution method. A series of concentrations of each marine fungus extracts ranging among 2.5 and 0.07 mg/mL prepared by twofold dilution were prepared in a 96-wells plate and tested in the medium Mueller–Hinton broth (Sigma Aldrich) for bacterial strains or in Sabauraud broth for the tested yeast. A test microbial suspension was obtained starting from a culture grown at 37 °C for 24 h on Tryptic Soy Agar (TSA) for bacterial strains, or Sabouraud agar for the yeast and adjusted to McFarland standard 0.5 (corresponding to 1.5 × 10^8^ CFU/mL) with 0.9% NaCl and 10 μL of each microbial suspension were added to all the wells of a 96-well microtiter plate, containing 100 µL of culture medium. A positive control to check microbial growth, consisting of tested microorganism in the medium without sample, and a negative control to check the medium sterility, represented by the medium without inoculum, were also included in the 96-wells plate. Moreover, a substance control to evaluate the absorbance of extracts, consisting only of the sample solution without microbial inoculum, was added. A DMSO control (tested microorganism in the medium with 2% *v*/*v* or less of DMSO in according to tested concentrations of samples), to exclude biological activity by diluent, was also added. The 96-well plates were incubated at 37 °C for 24 h and MICs were read by a microplate spectrophotometer (GloMax^®^-Multi Detection System, Promega) as the lowest concentration of extract whose OD, read at 570 nm, was comparable with the negative control wells (broth without microbial inoculum).

### Determination of minimum bactericidal (MBCs) and fungicidal concentrations (MFCs)

The minimum bactericidal or fungicidal concentration were also evaluated by subculturing 100 µL from each sample wells and form the positive control of MIC determination, onto substance-free Tryptone Soy agar (TSA) or SB (SABOURAUD + agar) plates without compounds. The plates were incubated at 37 °C for 24 h. The MBC and MFC were defined as the lowest concentration of compound that enable the bacterial growth up to a maximum of three colonies.

### Inhibition of biofilm formation assay

Microbial strains were incubated in Tryptic Soy Broth (TSB) containing 2% (w/v) glucose or in Sabouraud broth, in the case of yeast strains, at 37 °C for 24 h. After incubation, 2.5 μL of each microbial suspension were placed into the wells of a sterile flat-bottom 96-well microtiter plate containing 200 μL of medium. Aliquots at sub-MIC concentration of each compound, ranging from 6.25 to 50 μg/mL, were added directly to the wells. A positive control to check microbial growth (consisting of tested microorganisms in the medium, without compounds) and a negative control to check the medium sterility (represented by the medium without microbial suspension) were also included in the 96-well plate. The microplates were incubated at 37 °C for 24 h to allow biofilm formation. After biofilm growth, wells were washed twice with sterile NaCl 0.9%, and sessile biomass stained with 100 μL of 0.1% crystal violet solution for 30 min at 37 °C. Then, the plate was washed twice 200 μL of ethanol was added into each stained well and incubated for 10 min at room temperature. OD was read at a wavelength of 540 nm using a plate reader (Glomax Multidetection System TM297). The percentage of biofilm inhibition formation was determined through the following formula:

$$\% \,{\mathrm{Inhibition}} = [({\mathrm{OD}}\,{\mathrm{growth}} - {\mathrm{OD}}\,{\mathrm{sample}})/{\mathrm{OD}}\,{\mathrm{growth}}\,{\mathrm{control}}] \times 100.$$

BIC_50_, that is, the concentration at which the percentage of inhibition of biofilm formation is equal to 50%, was obtained by comparing the ODs of control wells with that of the sample wells at different concentrations, and the value was calculated using AAT Bioquest, Inc. Quest Graph™ IC_50_ Calculator (v.1) retrieved from https://www.aatbio.com/tools/ic50-calculator-v1. Each assay was performed in triplicate and independently repeated at least three times [52].

### OSMAC strategy-based fermentation and optimization of peptaibols production

To optimize peptaibol biosynthesis, strain IG119 was cultivated under different media, salinity and culture conditions (Table 4). The experimental design included eighteen solid culture conditions obtained by combining three media (PDA, MEA and Czapek Agar 49 g/L CZA, Difco Laboratories, Detroit, Mich, USA), three salinities (0, 40 and 80‰ sea salt), and the presence or absence of Corn Steep Liquor (CSL 1 g/L) as possible elicitor. In addition, six submerged culture conditions were tested using the same media without agar (PDB, MEB, CZB) at 40‰ salinity under both static and shaken incubation.

**Table 4:** OSMAC approach

Culture type	Medium	Salinity	CSL suppl	Incubation condition	No. of conditions
Submerged cultures	PDB	40 g/L	–	Static/shaken	2
	MEB	40 g/L	–	Static/shaken	2
	CZB	40 g/L	–	Static/shaken	2
Solid cultures	PDA	0, 40, 80 g/L	±CSL (1 g/L)	Static	6
	MEA	0, 40, 80 g/L	±CSL (1 g/L)	Static	6
	CZA	0, 40, 80 g/L	±CSL (1 g/L)	Static	6
				Total	24

For each experiment carried out on solid media six plates were inoculated as described above and incubated at 23 °C for 30 days. For submerged conditions, four 250 mL Erlenmeyer flasks containing 50 mL of medium were inoculated with 2 mg/mL (dry weight) of mycelium obtained from liquid cultures set up with the same medium. Cultures were incubated at 23 °C for 30 days in a thermostatic chamber (static condition) and in an orbital shaker at 150 rpm (shaken cultures).

Crude extracts (CEs) from solid cultures were obtained as described above using ethyl acetate (EtOAc) as solvent. For submerged cultures, CEs were obtained as follows: mycelial biomass was separated from the broth by filtration, and the broth was extracted overnight by liquid–liquid (1:1) extraction using EtOAc. The organic phase was then separated and concentrated by rotary evaporation. For each condition, uninoculated samples (2 plates and 1 flask for each condition) were set up and processed as the inoculated samples; the extracts were used as control.

### Large scale cultivation, extraction and isolation procedures

Eight hundred plates containing MEA supplemented with CSL (1 g/L) were inoculated. Crude extracts obtained from EtOAc extractions were obtained as described above. Removal of the solvent under reduced pressure afforded 3.8 g of crude extract which was subsequently partitioned between MeOH/H_2_O (9:1, *v*/*v*) and n-hexane. This liquid–liquid partitioning yielded 2.3 g of methanol extract and 1.0 g of hexane extract. A total of 760 mg of the hexane extract was subjected to flash chromatography (Biotage^®^ Selekt system), using a gradient from 100% chloroform to 100% methanol, yielding 11 fractions. An aliquot of fraction F2 (80 mg) was further purified by semi-preparative HPLC (Waters model 510 pump equipped with Waters Rheodine injector and a differential refractometer, model 401) on a C18 Luna column (250 × 10.0, Phenomenex), using MeOH/H_2_O (99:1, *v*/*v*) as the mobile phase, affording 5α,8α-epidioxy-22*E*-ergosta-6,22-dien-3β-ol (28 mg, t_R_ = 11.3 min) and ergosterol (8.6 mg, t_R_ = 22 min). 2.3 g of methanol extract was subjected to reverse-phase RP-18 flash chromatography (Biotage^®^ Selekt System), using a gradient from 100% H_2_O to 100% MeOH, yielding twelve fractions. Fractions 9–11 were further purified by DAD-HPLC (Agilent 1260 Infinity II). Separation was achieved employing a Synergi 4.0 µm Hydro-RP 80 Ǻ C18 (250 × 4.6, Phenomenex) with H_2_O 0.1% formic acid as eluent A and ACN 0.1% formic acid as eluent B. The gradient, with a 1.0 mL/min flow rate, was as follows: 60% B for 5 min, 60−77% B from 5 to 7 min, 77−83% B from 7 to 40 min, 100% B from 40 to 45 min, 100% B from 45 to 50 min. The column was then equilibrated at 60% B for 5 min, with a total chromatographic run of 55 min. The detection of peptaibols took place in DAD at 220 nm.

NMR spectra were obtained on Bruker Avance NEO 600 and 700 spectrometers (Bruker Company Billerica, MA, USA) equipped with an RT-DR-BF/1H-5 mm-OZ SmartProbe (^1^H at 600 MHz and ^13^C at 151 MHz; ^1^H at 700 MHz and ^13^C at 176 MHz) and recorded in DMSO-d_6_ (*δ*_H_ 2.5 and *δ*_C_ 39.5 ppm) and acetone-d_6_ (*δ*_H_ 2.05 and *δ*_C_ 29.9 and 206.0 ppm). ^13^C–^1^H connectivities were determined using HSQC, HMBC and 2D HSQC-TOCSY experiments, while ^1^H–^1^H connectivities were determined through COSY, TOCSY and NOESY experiments.

Optical rotations were measured on a Perkin-Elmer 141 polarimeter using a sodium lamp operating at 589 nm in MeOH solutions. CD spectra were recorded in the far-UV region on a Jasco J-815 spectropolarimeter (JASCO, Tokyo, Japan) at 25 ^◦^C using a 0.1 cm path-length quartz cuvette.

Roseabol B (**1**) white amorphous solid; t_R_ 30 min; $\alpha_{25}^D$ −3.96 (*c* 0.37, methanol); ^1^H and ^13^C NMR data in DMSO-d_6_ see Table 1 and in acetone-*d*_6_ see Table S5, HR-ESI MS data *m*/*z* 1365.8629 [M + H]^+^ (calcd for C_66_H_117_N_12_O_18_, 1365.8603).

Roseabol C (**2**). white amorphous solid; t_R_ 22.2 min $\alpha_{25}^D$ −13.0 (*c* 0.11, methanol); selected NH, Hα and Cα NMR assignments in DMSO-*d*_6_ are reported in Table S6; HR-ESI MS data *m*/*z* 1210.8039 [M + H]^+^ (calcd for C_59_H_108_N_11_O_15_, 1210.8021).

### LC–HRMS/MS analysis and feature-based molecular networking

For LC–HRMS/MS analysis, the extracts obtained from the small-scale cultivation of *C. rosea* IG119 on ME agar (CHCl_3_, BuOH, and H_2_O) and from the OSMAC experiments, were dissolved in LC–MS grade MeOH to a final concentration of 1 mg/mL. Analyses were performed using a Thermo Scientific Q Exactive Focus Orbitrap mass spectrometer coupled to a Thermo Ultimate 3000 HPLC system. Chromatographic separation was achieved on a Hypersil C18 column (100 × 4.6 mm, 3 μm) maintained at 25 °C. The mobile phase consisted of H_2_O (A) and CH_3_CN (B), both acidified with 0.1% formic acid. The following gradient was applied at a constant flow rate of 400 µL/min: 0% B for 1 min, a linear increase from 0 to 100% B over 30 min, followed by an isocratic wash at 100% B for 10 min.

Mass spectra were acquired in positive electrospray ionization (ESI+) mode with a mass accuracy of ≤3 ppm. Ion source parameters were set as follows: spray voltage, 4.8 kV; capillary temperature, 285 °C; sheath gas flow rate, 32 arbitrary units (N_2_); and auxiliary gas flow rate, 15 arbitrary units (N_2_). MS/MS data were acquired in data-dependent acquisition (DDA) mode, targeting the three most intense precursor ions from each full scan for fragmentation. The full scan range was set to *m*/*z* 300–2000. High-resolution MS/MS spectra were generated via higher-energy collisional dissociation (HCD) using an isolation width of 2.0 m/*z*, a normalized collision energy (NCE) of 15 units, and automated injection time control.

The LC–HRMS/MS analysis of the EtOAc extract from the large-scale cultivation of *C. rosea* IG119 (Figure S2) on ME agar (0% salinity, supplemented with CSL), followed the same general workflow but with an optimized gradient for peptaibol separation: a linear increase from 70 to 80% B over 3 min, an isocratic step at 80% B for 1 min, a linear gradient from 80 to 100% B over 21 min, and a final 10-min isocratic wash at 100% B. For this analysis, MS data were acquired using a full scan range of *m*/*z* 900–2500.

Raw files from LC–HRMS/MS analyses were processed using MZmine (v. 2.53), following the parameters detailed in Table S9, to generate a.mgf file, as previously reported [53]. This file was then uploaded to the GNPS2 Analysis Hub (https://gnps2.org) for FBMN. The resulting networks were visualized using Cytoscape software (v. 3.8.2). Comprehensive data, including molecular networks, FBMN parameters, and mass spectra of individual compounds, are publicly available at the following GNPS2 links: https://gnps2.org/status?task=d8207dde29db4147b641c1aa1d169cae (Figure S1), https://gnps2.org/status?task=42cb8bb32c754cba93d20be5490abd61 (Fig. 1), and https://gnps2.org/status?task=213f8a0100ca4cbd911b38fd10d55213 (Figure S2).

### Advanced Marfey’s analysis

For the advanced Marfey’s analysis, roseabols hydrolysis was performed as previously reported [54]. Marfey’s derivatives of roseabols B (**1**) and C (**2**) were analyzed by HPLC–ESI–HRMS using the instrumentation described in Sect. 3.8. Absolute configurations were assigned by comparing their retention times with those of authentic standard derivatives. Chromatographic separation was performed on a Hypersil C18 column at room temperature with a flow rate of 400 μL/min. The mobile phase consisted of H_2_O (A) and CH_3_CN (B), both containing 0.1% HCOOH. The gradient program was set as follows: 5% B for 3 min, a linear gradient from 5 to 70% B over 30 min, and an isocratic wash at 100% B for 7 min. Considering that Gln is transformed into Glu during hydrolysis [55], the l- and d-Glu standard derivatives and l-FDAA derivatives obtained from **1** and **2**, were analyzed by HPLC–ESI–HRMS to assign Gln configuration, using a modified gradient: 5% B for 3 min, a linear gradient from 5 to 50% B over 30 min, and a final wash at 100% B for 7 min.

### Sequencing and bioinformatics

High-molecular-weight DNA was extracted from 7-day-old cultures grown on Malt Extract Broth using the DNeasy Plant Pro Kit (Qiagen, Hilden, Germany), following the manufacturer’s instructions. The extracted DNA was checked for integrity by capillary electrophoresis (HS Genomic DNA 50 kb Kit; Fragment Analyzer 5200) and DNA concentration was measured using Qubit dsDNA BR Assay Kit (Invitrogen).

A total of 0.4 µg of genomic DNA was used for Nanopore library preparation using the Native Barcoding Kit 96 V14 (SQK-NBD114.96, Oxford, United Kingdom). Sequencing was performed using a PromethION 2 Solo device (Oxford Nanopore Technologies) and base calling was performed using dorado v.1.1.0 with the Super Accurate (SUP) model.

Genome assembly was performed using the nf-core/genomeassembler pipeline (v1.1.0) (https://nf-co.re/genomeassembler/1.1.0) [56], executed through the Nextflow workflow manager [57]. Long read adapter trimming was performed with Porechop version 0.2.4 [58] and high-quality long reads were assembled using Flye version 2.9 [59]. The assembled genome was further polished using Medaka version 1.7.1, thus obtaining 151 contigs.

The genome assembly quality was assessed using QUAST v. 5.2.0 [60] and Benchmarking Universal Single-Copy Orthologs (BUSCO) v 5.8.3 and completeness was evaluated using the fungi_odb10 database [61]. Default parameters were used for all tools.

Following gene prediction with AUGUSTUS [62], BGCs within the fungal genome were identified using antiSMASH 8.0 [40]. The roseabol gene cluster was deposited in GenBank under accession number PZ405919.

## Conclusion

This study demonstrates that a metabolomics-driven approach combining OSMAC cultivation, molecular networking, and targeted isolation is effective in exploring the chemical diversity of the marine fungus *Clonostachys rosea* IG119. This strategy enabled the identification of a highly complex and microheterogeneous peptaibol profile and the isolation of two new compounds, roseabols B (**1**) and C (**2**), whose structures were elucidated by HRMS/MS, NMR, and Marfey’s analysis. The two isolated compounds showed an antibiofilm activity towards *C. albicans* and *E. coli*, highlighting their potential as promising leads for the development of antivirulence strategies aimed at controlling biofilm-associated infections. The observed structural variability reflects the flexibility of the NRPS biosynthetic machinery, expanding the known peptaibol landscape and confirming marine fungi as valuable sources of novel bioactive metabolites.

## Supplementary Information

Additional file 1.

## Data Availability

The experimental data supporting this work is accessible within the article and its additional file.
