# Supplementary material for: Uncovering new antibiofilm roseabols from marine Clonostachys rosea through structural and multiomics approaches
Source: Nat Prod Bioprospect. 2026 Nov 11;16(1):104. doi: 10.1007/s13659-026-00659-3 (PMC13755224; doi:10.1007/s13659-026-00659-3)

Supplementary Material

Uncovering New Antibiofilm Roseabols from Marine *Clonostachys rosea* through Structural and Multiomics Approaches

Gerardo Della Sala[1†], Carmen Festa[2†], Mattia Cammarota[2], Marcella Pasqualetti[3], Martina Braconcini[3], Donatella de Pascale[1], Valentina Catania[4], Donatella Ferraro[5], Domenico Schillaci[5], Simona De Marino[2*], Massimiliano Fenice[3], Maria Valeria D'Auria[2]

[1]Department of Ecosustainable Marine Biotechnology, Stazione Zoologica Anton Dohrn, Naples, Italy;
[2]Department of Pharmacy, University of Naples "Federico II", Naples, Italy;
[3]Department of Biological and Ecological Sciences, University of Tuscia, Viterbo, Italy;
[4]Department of Earth and Marine Sciences (DiSTeM), University of Palermo, Palermo, Italy;
[5]Department of Biological, Chemical and Pharmaceutical Sciences and Technologies (STEBICEF), University of Palermo, Palermo, Italy.

[†]These authors have contributed equally to this work.
***Correspondence**: Simona De Marino simona.demarino@unina.it

Table of Contents

Table S1. Antimicrobial activity of $CHCl_3$ and *n*-BuOH extracts of *C. rosea* p.4

Table S2. Structures of integramides from *C. rosea* p.4

Table S3. Structures of 11-mer roseabols from *C. rosea* p.5

Table S4. Structures of 10-mer roseabols from *C. rosea* p.8

Table S5. ^{1}H and ^{13}C NMR data (600 and 150 MHz, acetone-d_6) of roseabol B (**1**) p.11

Table S6. ^{1}H and ^{13}C NMR data (600 and 150 MHz, DMSO-d_6) of roseabol C (**2**) p.12

Table S7. Putative genes flanking the *rsb* gene cluster p.13

Table S8. PARAS substrate prediction and binding pocket signatures of adenylation domains from the Rsb synthetase p.14

Table S9. MZmine parameters for MS raw data processing p.15

Figure S1. Molecular network of $CHCl_3$, *n*-BuOH, and H_2O extracts from *C. rosea* p.16

Figure S2. Molecular network of the EtOAc extract p.16

Figure S3. Advanced Marfey's analysis of roseabol B (**1**) p.17

Figure S4. COSY/TOCSY correlations (in bold) and MS^2 fragmentations of roseabol C (**2**) p.17

Figure S5. ^{1}H NMR (700 MHz, DMSO-d_6) of roseabol B (**1**) p.18

Figure S6. Expanded ^{1}H NMR spectrum (700 MHz, DMSO-d_6) of the NH region of roseabol B (**1**) p.18

Figure S7. ^{13}C NMR (176 MHz, DMSO-d_6) of roseabol B (**1**) p.19

Figure S8. Expanded ^{13}C NMR spectrum (176 MHz, DMSO-d_6) of the carbonyl region of roseabol B (**1**) p.19

Figure S9. HSQC (700 MHz, DMSO-d_6) of roseabol B (**1**) p.20

Figure S10. HMBC (700 MHz, DMSO-d_6) of roseabol B (**1**) p.20

Figure S11. TOCSY (700 MHz, DMSO-d_6) of roseabol B (**1**) p.21

Figure S12. COSY (700 MHz, DMSO-d_6) of roseabol B (**1**) p.21

Figure S13. 2D HSQC-TOCSY (700 MHz, DMSO-d_6) of roseabol B (**1**) p.22

Figure S14. NOESY (700 MHz, DMSO-d_6) of roseabol B (**1**) p.22

Figure S15. ^{1}H NMR (600 MHz, acetone-d_6) of roseabol B (**1**) p.23

Figure S16. ^{13}C NMR (151 MHz, acetone-d_6) of roseabol B (**1**) p.23

Figure S17. HSQC (600 MHz, acetone-d_6) of roseabol B (**1**) p.24

Figure S18. HMBC (600 MHz, acetone-d_6) of roseabol B (**1**) p.24

Figure S19. TOCSY (600 MHz, acetone-d_6) of roseabol B (**1**) p.25

Figure S20. COSY (600 MHz, acetone-d_6) of roseabol B (**1**) p.25

Figure S21. 2D HSQC-TOCSY (600 MHz, acetone-d_6) of roseabol B (**1**) p26

Figure S22. ^{1}H NMR (600 MHz, DMSO-d_6) of roseabol C (**2**) p.27

Figure S23. COSY (600 MHz, DMSO-d_6) of roseabol C (**2**) p.28

Figure S24. TOCSY (600 MHz, DMSO-d_6) of roseabol C (**2**) p.28

Figure S25. HSQC (600 MHz, DMSO-d_6) of roseabol C (**2**) p.29

Figure S26. 2D HSQC-TOCSY (600 MHz, DMSO-d_6) of roseabol C (**2**) p.29

Figure S27. HMBC (600 MHz, DMSO-d_6) of roseabol C (**2**) p.30

Table S1. Antimicrobial activity of $CHCl_3$ and *n*-BuOH extracts of *Clonostachys rosea* IG119 cultured on MEA medium

Extract	*Staphylococcus aureus* ATCC 25923	*Pseudomonas aeruginosa* ATCC 1544	*Escherichia coli* ATCC 25922	*Enterococcus faecalis* ATCC 29212
$CHCl_3$	0.3 mg/ml	1.2 mg/ml	0.3 mg/ml	>2.5 mg/ml
n-BuOH	>2.5 mg/ml	2.5 mg/ml	>2.5 mg/ml	>2.5 mg/ml

Table S2. Structures of integramides from *Clonostachys rosea* IG119

	$[M+H]^+$	*m/z*	Δ (ppm)	R_t (min)	aa sequence
Integramide D (**6**)	$C_{76}H_{131}N_{16}O_{21}$	1603.9694	1.5	8.9	Ac-Aib-Hyp-Ile-Aib-Leu-Aib-Aib-Aib-Hyp-Leu-Iva-Aib-Hyp-Iva-Iva-Gly
Integramide E (**7**)	$C_{77}H_{133}N_{16}O_{21}$	1617.9854	1.8	10.3	Ac-Iva-Hyp-Ile-Aib-Leu-Aib-Aib-Aib-Hyp-Leu-Iva-Aib-Hyp-Iva-Iva-Gly
Integramide A (**3**)	$C_{78}H_{135}N_{16}O_{21}$	1632.0019	2.3	11.3	Ac-Iva-Hyp-Ile-Iva-Leu-Aib-Aib-Aib-Hyp-Leu-Iva-Aib-Hyp-Iva-Iva-Gly
Integramide C (**5**)	$C_{78}H_{135}N_{16}O_{21}$	1632.0001	1.2	12.0	Ac-Iva-Hyp-Ile-Aib-Leu-Aib-Aib-Val/Iva-Hyp-Leu-Iva-Aib-Hyp-Iva-Iva-Gly
Integramide B (**4**)	$C_{79}H_{137}N_{16}O_{21}$	1646.0180	2.5	13.3	Ac-Iva-Hyp-Ile-Iva-Leu-Aib-Aib-Iva-Hyp-Leu-Iva-Aib-Hyp-Iva-Iva-Gly

Table S3. Structures of 11-mer roseabols from *Clonostachys rosea* IG119. [a]Truncated 11-mer roseabols variants

	$[M+H]^+$	*m/z*	Δ (ppm)	R_t (min)	aa sequence
**8**	$C_{65}H_{115}N_{12}O_{19}S$	1399.8127	0.7	4.0	Isovaleryl-Iva-Ser-Val-MetO-Aib-Hyp-Leu-Leu-Gln-Ser(Ac)-Leuol-Isovaleryl
**9**	$C_{49}H_{86}N_9O_{15}$	1040.6250	1.2	5.0	Isovaleryl-Aib-Ser-Val-Leu-Aib-Hyp-Leu-Leu-Glu[a]
**10**	$C_{49}H_{86}N_9O_{14}$	1024.6310	2.1	5.2	Isovaleryl-Aib-Ser-Val-Leu-Aib-Pro-Leu-Leu-Glu[a]
**11**	$C_{49}H_{89}N_{10}O_{14}$	1053.6568	1.3	5.4	Isovaleryl-Iva-Ser-Val-Leu-Aib-Hyp-Leu-Leu-Gln[a]
**12**	$C_{50}H_{88}N_9O_{15}$	1054.6400	0.6	5.4	Isovaleryl-Iva-Ser-Val-Leu-Aib-Hyp-Leu-Leu-Glu[a]
**13**	$C_{51}H_{90}N_9O_{15}$	1068.6552	0.1	5.5	Isovaleryl-Iva-Ser-Val-Leu-Iva/Val-Hyp-Leu-Leu-Glu[a]
**14**	$C_{44}H_{79}N_8O_{12}$	911.5816	0.5	5.6	Isovaleryl-Aib-Ser-Val-Leu-Aib-Hyp-Leu-Leu[a]
**15**	$C_{51}H_{90}N_9O_{15}$	1068.6562	1.1	6.1	Isovaleryl-Iva-Ser-Val-Leu-Aib-Hyp-Leu-Leu-Glu-OMe[a]
**16**	$C_{45}H_{81}N_8O_{12}$	925.5972	0.4	6.1	Isovaleryl-Iva-Ser-Val-Leu-Aib-Hyp-Leu-Leu[a]
**17**	$C_{50}H_{90}N_{11}O_{13}$	1052.6712	0.2	6.1	Isovaleryl-Iva-Ser-Val-Leu-Aib-Hyp-Leu-Leu-Gln-NH_2[a]
**18**	$C_{45}H_{82}N_9O_{11}$	924.6150	2.3	6.6	Isovaleryl-Iva-Ser-Val-Leu-Aib-Hyp-Leu-Leu-NH_2[a]
**19**	$C_{47}H_{83}N_8O_{13}$	967.6083	0.9	6.9	Isovaleryl-Iva-Ser(Ac)-Val-Leu-Aib-Hyp-Leu-Leu[a]
**20**	$C_{58}H_{105}N_{12}O_{16}$	1225.7778	1.0	8.4	Isovaleryl-Aib-Ser-Val-Leu-Aib-Hyp-Leu-Leu-Gln-Ser-Leuol
**21**	$C_{60}H_{107}N_{12}O_{17}$	1267.7889	1.4	9.0	Ac-Aib-Ser-Val-Leu-Aib-Hyp-Leu-Leu-Gln-Ser-Leuol-Isovaleryl
**22**	$C_{58}H_{105}N_{12}O_{15}$	1209.7833	1.3	9.1	Isovaleryl-Aib-Ala-Val-Leu-Aib-Hyp-Leu-Leu-Gln-Ser-Leuol
**23**	$C_{59}H_{107}N_{12}O_{16}$	1239.7940	1.4	9.3	Isovaleryl-Iva-Ser-Val-Leu-Aib-Hyp-Leu-Leu-Gln-Ser-Leuol
**24**	$C_{62}H_{111}N_{12}O_{17}$	1295.8210	2.0	9.6	Propionyl-Iva-Ser-Val-Leu-Aib-Hyp-Leu-Leu-Gln-Ser-Leuol-Isovaleryl
**25**	$C_{61}H_{109}N_{12}O_{17}$	1281.8054	2.0	9.7	Ac-Iva-Ser-Val-Leu-Aib-Hyp-Leu-Leu-Gln-Ser-Leuol-Isovaleryl
**26**	$C_{60}H_{107}N_{12}O_{17}$	1267.7893	1.7	9.8	Isovaleryl-Aib-Ser-Val-Leu-Aib-Hyp-Leu-Leu-Gln-Ser-Leuol-Ac
**27**	$C_{60}H_{107}N_{12}O_{17}$	1267.7893	1.7	9.8	Isovaleryl-Aib-Ser-Val-Leu-Aib-Hyp-Leu-Leu-Gln-Ser(Ac)-Leuol
**28**	$C_{61}H_{109}N_{12}O_{17}$	1281.8053	1.9	10.3	Isovaleryl-Iva-Ser-Val-Leu-Aib-Hyp(Ac)-Leu-Leu-Gln-Ser-Leuol
**29**	$C_{61}H_{109}N_{12}O_{17}$	1281.8061	2.6	10.9	Isovaleryl-Iva-Ser-Val-Leu-Aib-Hyp-Leu-Leu-Gln-Ser-Leuol-Ac
**30**	$C_{63}H_{111}N_{12}O_{18}$	1323.8150	1.2	11.0	Ac-Iva-Ser-Val-Leu-Aib-Hyp-Leu-Leu-Gln-Ser(Ac)-Leuol-Isovaleryl
**31**	$C_{62}H_{111}N_{12}O_{17}$	1295.8217	2.5	11.4	Isovaleryl-Iva-Ser-Val-Leu-Iva/Val-Hyp-Leu-Leu-Gln-Ser-Leuol-Ac

**32**	$C_{61}H_{109}N_{12}O_{16}$	1265.8116	2.9	11.9	Isovaleryl-Iva-Ser-Val-Leu-Aib-Pro-Leu-Leu-Gln-Ser(Ac)-Leuol
**33**	$C_{62}H_{111}N_{12}O_{17}$	1295.8199	1.1	12.0	Isovaleryl-Iva-Ser-Leu/Ile-Leu-Aib-Hyp-Leu-Leu-Gln-Ser(Ac)-Leuol
**34**	$C_{62}H_{111}N_{12}O_{17}$	1295.8199	1.1	12.0	Isovaleryl-Iva-Ser-Val-Aib-Aib-Hyp-Leu-Leu-Gln-Ser-Leuol-Isovaleryl
**35**	$C_{63}H_{111}N_{12}O_{18}$	1323.8163	2.2	12.3	Isovaleryl-Iva-Ser-Val-Leu-Aib-Hyp-Leu-Leu-Gln-Ser(Ac)-Leuol-Ac
**36**	$C_{61}H_{109}N_{12}O_{16}$	1265.8116	2.9	12.7	Isovaleryl-Iva-Ala-Val-Leu-Aib-Hyp-Leu-Leu-Gln-Ser-Leuol-Ac
**37**	$C_{68}H_{119}N_{12}O_{20}$	1423.8690	2.2	13.7	Isovaleryl-Iva-Ser(Succinyl)-Val-Leu-Aib-Hyp-Leu-Leu-Gln-Ser-Leuol-Isovaleryl
**38**	$C_{69}H_{119}N_{12}O_{21}$	1451.8594	-0.9	13.9	Isovaleryl-Aib-Ser(Succinyl)-Val-Leu-Aib-Hyp-Leu-Leu-Gln-Ser(Ac)-Leuol-Isovaleryl
**39**	$C_{63}H_{113}N_{12}O_{17}$	1309.8351	0.8	14.0	Isovaleryl-Aib-Ser-Val-Leu-Aib-Hyp-Leu-Leu-Gln-Ser-Leuol-Isovaleryl
**40**	$C_{63}H_{113}N_{12}O_{16}$	1293.8412	1.5	15.0	Isovaleryl-Aib-Ser-Val-Leu-Aib-Pro-Leu-Leu-Gln-Ser-Leuol-Isovaleryl
**41**	$C_{70}H_{121}N_{12}O_{21}$	1465.8794	2.1	15.3	Isovaleryl-Iva-Ser(Succinyl)-Val-Leu-Aib-Hyp-Leu-Leu-Gln-Ser(Ac)-Leuol-Isovaleryl
**42**	$C_{65}H_{115}N_{12}O_{18}$	1351.8462	0.7	15.4	Isovaleryl-Aib-Ser-Val-Leu-Aib-Hyp-Leu-Leu-Gln-Ser(Ac)-Leuol-Isovaleryl
**43**	$C_{64}H_{115}N_{12}O_{17}$	1323.8518	1.6	15.4	Isovaleryl-Iva-Ser-Val-Leu-Aib-Hyp-Leu-Leu-Gln-Ser-Leuol-Isovaleryl
**44**	$C_{65}H_{115}N_{12}O_{17}$	1335.8518	1.5	15.8	Isovaleryl-Aib-Ser-Val-Leu-Aib-Pro-Leu-Leu-Gln-Ser(Ac)-Leuol-Isovaleryl
**45**	$C_{63}H_{113}N_{12}O_{16}$	1293.8403	0.9	16.0	Isovaleryl-Aib-Ser-Val-Leu-Aib-Hyp-Leu-Leu-Gln-Ala-Leuol-Isovaleryl
**46**	$C_{65}H_{117}N_{12}O_{17}$	1337.8663	0.6	16.1	Isovaleryl-Iva-Ser-Val-Leu-Iva/Val-Hyp-Leu-Leu-Gln-Ser-Leuol-Isovaleryl
**47**	$C_{67}H_{117}N_{12}O_{19}$	1393.8585	2.3	16.3	Isovaleryl-Aib-Ser(Ac)-Val-Leu-Aib-Hyp-Leu-Leu-Gln-Ser(Ac)-Leuol-Isovaleryl
**48**	$C_{64}H_{115}N_{12}O_{16}$	1307.8583	2.6	16.4	Isovaleryl-Iva-Ser-Val-Leu-Aib-Pro-Leu-Leu-Gln-Ser-Leuol-Isovaleryl
**49**	$C_{65}H_{115}N_{12}O_{17}$	1335.8530	2.4	16.5	Isovaleryl-Aib-Ala-Val-Leu-Aib-Hyp-Leu-Leu-Gln-Ser(Ac)-Leuol-Isovaleryl
**50**	$C_{65}H_{117}N_{12}O_{17}$	1337.8677	1.7	16.8	Isovaleryl-Iva-Ser-Leu/Ile-Leu-Aib-Hyp-Leu-Leu-Gln-Ser-Leuol-Isovaleryl
**Roseabol B (1)**	$\mathbf{C_{66}H_{117}N_{12}O_{18}}$	**1365.8629**	**1.8**	**16.9**	**Isovaleryl-Iva-Ser-Val-Leu-Aib-Hyp-Leu-Leu-Gln-Ser(Ac)-Leuol-Isovaleryl**
**51**	$C_{66}H_{117}N_{12}O_{17}$	1349.8684	2.2	17.2	Isovaleryl-Iva-Ser-Val-Leu-Aib-Pro-Leu-Leu-Gln-Ser(Ac)-Leuol-Isovaleryl
**52**	$C_{67}H_{119}N_{12}O_{18}$	1379.8775	1.1	17.4	Isovaleryl-Iva-Ser-Val-Leu-Iva/Val-Hyp-Leu-Leu-Gln-Ser(Ac)-Leuol-Isovaleryl
**53**	$C_{64}H_{115}N_{12}O_{16}$	1307.8588	3.0	17.6	Isovaleryl-Iva-Ser-Val-Leu-Aib-Hyp-Leu-Leu-Gln-Ala-Leuol-Isovaleryl
**54**	$C_{67}H_{119}N_{12}O_{17}$	1363.8849	2.8	17.6	Isovaleryl-Iva-Ser-Val-Leu-Iva/Val-Pro-Leu-Leu-Gln-Ser(Ac)-Leuol-Isovaleryl
**55**	$C_{68}H_{119}N_{12}O_{19}$	1407.8733	1.7	17.8	Isovaleryl-Iva-Ser(Ac)-Val-Leu-Aib-Hyp-Leu-Leu-Gln-Ser(Ac)-Leuol-Isovaleryl
**56**	$C_{66}H_{117}N_{12}O_{17}$	1349.8693	2.8	18.1	Isovaleryl-Iva-Ala-Val-Leu-Aib-Hyp-Leu-Leu-Gln-Ser(Ac)-Leuol-Isovaleryl

**57**	$C_{67}H_{119}N_{12}O_{18}$	1379.8763	0.3	18.2	Isovaleryl-Iva-Ser-Ile/Leu-Leu-Aib-Hyp-Leu-Leu-Gln-Ser(Ac)-Leuol-Isovaleryl
**58**	$C_{65}H_{117}N_{12}O_{16}$	1321.8745	3.0	18.2	Isovaleryl-Iva-Ala-Leu/Ile-Leu-Aib-Hyp-Leu-Leu-Gln-Ser-Leuol-Isovaleryl
**59**	$C_{65}H_{117}N_{12}O_{16}$	1321.8745	3.0	18.2	Isovaleryl-Iva-Ser- Leu/Ile -Leu-Aib-Pro-Leu-Leu-Gln-Ser-Leuol-Isovaleryl
**60**	$C_{67}H_{119}N_{12}O_{17}$	1363.8838	2.0	18.5	Isovaleryl-Iva-Ala-Val-Leu-Iva/Val-Hyp-Leu-Leu-Gln-Ser(Ac)-Leuol-Isovaleryl
**61**	$C_{66}H_{117}N_{12}O_{16}$	1333.8719	1.1	18.6	Isovaleryl-Iva-Ala-Val-Leu-Aib-Pro-Leu-Leu-Gln-Ser(Ac)-Leuol-Isovaleryl
**62**	$C_{64}H_{115}N_{12}O_{15}$	1291.8637	2.9	18.9	Isovaleryl-Iva-Ser-Val-Leu-Aib-Pro-Leu-Leu-Gln-Ala-Leuol-Isovaleryl
**63**	$C_{69}H_{121}N_{12}O_{19}$	1421.8894	2.0	19.1	Isovaleryl-Iva-Ser(Ac)-Ile/Leu-Leu-Aib-Hyp-Leu-Leu-Gln-Ser(Ac)-Leuol-Isovaleryl
**64**	$C_{65}H_{117}N_{12}O_{16}$	1321.8729	1.8	19.2	Isovaleryl-Iva-Ser-Leu/Ile-Leu-Aib-Hyp-Leu-Leu-Gln-Ala-Leuol-Isovaleryl
**65**	$C_{67}H_{119}N_{12}O_{17}$	1363.8848	2.8	19.3	Isovaleryl-Iva-Ala-Ile/Leu-Leu-Aib-Hyp-Leu-Leu-Gln-Ser(Ac)-Leuol-Isovaleryl
**66**	$C_{68}H_{121}N_{12}O_{18}$	1393.8940	1.7	20.3	Isovaleryl-Aib-Ser-Val-Leu-Aib-Hyp-Leu-Leu-Gln-Ser(Isovaleryl)-Leuol-Isovaleryl
**67**	$C_{68}H_{121}N_{12}O_{17}$	1377.9005	2.8	20.7	Isovaleryl-Aib-Ser-Val-Leu-Aib-Pro-Leu-Leu-Gln-Ser(Isovaleryl)-Leuol-Isovaleryl
**68**	$C_{70}H_{123}N_{12}O_{19}$	1435.9026	0.3	21.0	Isovaleryl-Aib-Ser(Ac)-Val-Leu-Aib-Hyp-Leu-Leu-Gln-Ser(Isovaleryl)-Leuol-Isovaleryl
**69**	$C_{68}H_{121}N_{12}O_{17}$	1377.9005	2.8	21.4	Isovaleryl-Aib-Ala-Val-Leu-Aib-Hyp-Leu-Leu-Gln-Ser(Isovaleryl)-Leuol-Isovaleryl
**70**	$C_{69}H_{123}N_{12}O_{18}$	1407.9085	0.9	22.1	Isovaleryl-Iva-Ser-Val-Leu-Aib-Hyp-Leu-Leu-Gln-Ser(Isovaleryl)-Leuol-Isovaleryl
**71**	$C_{69}H_{123}N_{12}O_{17}$	1391.9154	2.2	22.3	Isovaleryl-Iva-Ser-Val-Leu-Aib-Pro-Leu-Leu-Gln-Ser(Isovaleryl)-Leuol-Isovaleryl
**72**	$C_{70}H_{125}N_{12}O_{18}$	1421.9248	1.3	22.5	Isovaleryl-Iva-Ser-Val-Leu-Iva/Val-Hyp-Leu-Leu-Gln-Ser(Isovaleryl)-Leuol-Isovaleryl
**73**	$C_{70}H_{125}N_{12}O_{18}$	1421.9245	0.7	22.5	Isovaleryl-Iva-Ser-Val-Leu-Iva/Val-Hyp-Leu-Leu-Glu-Lys(Ac)-Leuol-Isovaleryl
**74**	$C_{70}H_{125}N_{12}O_{17}$	1405.9316	2.6	22.5	Isovaleryl-Iva-Ser-Val-Leu-Iva/Val-Pro-Leu-Leu-Gln- Ser(Isovaleryl)-Leuol-Isovaleryl
**75**	$C_{71}H_{125}N_{12}O_{19}$	1449.9221	2.9	22.7	Isovaleryl-Iva-Ser(Ac)-Val-Leu-Aib-Hyp-Leu-Leu-Gln- Ser(Isovaleryl)-Leuol-Isovaleryl
**76**	$C_{69}H_{123}N_{12}O_{17}$	1391.9153	2.1	23.1	Isovaleryl-Iva-Ala-Val-Leu-Aib-Hyp-Leu-Leu-Gln-Ser(Isovaleryl)-Leuol-Isovaleryl
**77**	$C_{70}H_{125}N_{12}O_{18}$	1421.9264	2.4	23.5	Isovaleryl-Iva-Ser-Leu/Ile-Leu-Aib-Hyp-Leu-Leu-Gln-Ser(Isovaleryl)-Leuol-Isovaleryl
**78**	$C_{70}H_{125}N_{12}O_{17}$	1405.9324	3.1	23.7	Isovaleryl-Iva-Ala-Val-Leu-Iva/Val-Hyp-Leu-Leu-Gln- Ser(Isovaleryl)-Leuol-Isovaleryl

Table S4. Structures of 10-mer roseabols from *Clonostachys rosea* IG119. [a]Truncated 10-mer roseabols variants.

	$[M+H]^+$	*m/z*	Δ (ppm)	R_t (min)	aa sequence
**79**	$C_{48}H_{79}N_8O_{13}$	927.5769	0.9	4.2	Isovaleryl-Aib-Ser-Val-Leu-Aib-Leu-Leu-Glu[a]
**80**	$C_{45}H_{82}N_9O_{12}$	940.6079	0.2	4.3	Isovaleryl-Iva-Ser-Val-Leu-Aib-Leu-Leu-Gln[a]
**81**	$C_{45}H_{81}N_8O_{13}$	941.5936	2.0	4.5	Isovaleryl-Iva-Ser-Val-Leu-Aib-Leu-Leu-Glu[a]
**82**	$C_{53}H_{98}N_{11}O_{14}$	1112.7288	-0.1	7.4	Isovaleryl-Aib-Ser-Val-Leu-Aib-Leu-Leu-Gln-Ser-Leuol
**83**	$C_{54}H_{100}N_{11}O_{14}$	1126.7435	-1.4	7.9	Isovaleryl-Iva-Ser-Val-Leu-Aib-Leu-Leu-Gln-Ser-Leuol
**84**	$C_{55}H_{100}N_{11}O_{15}$	1154.7397	0.2	7.9	Isovaleryl-Aib-Ser-Val-Leu-Aib-Leu-Leu-Gln-Ser(Ac)-Leuol
**85**	$C_{55}H_{100}N_{11}O_{15}$	1154.7390	-0.5	8.0	Isovaleryl-Aib-Ser-Val-Leu-Aib-Leu-Leu-Gln-Ser(Ac)-Leuol
**86**	$C_{55}H_{102}N_{11}O_{14}$	1140.7620	1.6	8.1	Isovaleryl-Iva-Ser-Val-Leu-Iva/Val-Leu-Leu-Gln-Ser-Leuol
**87**	$C_{53}H_{98}N_{11}O_{13}$	1096.7357	1.5	8.3	Isovaleryl-Aib-Ser-Val-Leu-Aib-Leu-Leu-Gln-Ala-Leuol
**88**	$C_{55}H_{100}N_{11}O_{15}$	1154.7406	1.0	8.5	Isovaleryl-Aib-Ser-Val-Leu-Aib-Leu-Leu-Gln-Ser-Leuol-Ac
**89**	$C_{56}H_{102}N_{11}O_{15}$	1168.7560	0.7	8.6	Isovaleryl-Iva-Ser-Val-Leu-Aib-Leu-Leu-Gln-Ser(Ac)-Leuol
**90**	$C_{55}H_{102}N_{11}O_{14}$	1140.7618	1.4	8.8	Isovaleryl-Iva-Ser-Leu/Ile-Leu-Aib-Leu-Leu-Gln-Ser-Leuol
**91**	$C_{57}H_{104}N_{11}O_{15}$	1182.7732	2.0	8.8	Isovaleryl-Iva-Ser-Val-Leu-Iva/Val-Leu-Leu-Gln-Ser(Ac)-Leuol
**92**	$C_{56}H_{102}N_{11}O_{15}$	1168.7562	0.9	9.0	Isovaleryl-Iva-Ser-Val-Leu-Aib-Leu-Leu-Gln-Ser-Leuol-Ac
**93**	$C_{54}H_{100}N_{11}O_{13}$	1110.7516	1.8	9.0	Isovaleryl-Iva-Ser-Val-Leu-Aib-Leu-Leu-Gln-Ala-Leuol
**94**	$C_{54}H_{100}N_{11}O_{13}$	1110.7516	1.8	9.0	Isovaleryl-Iva-Ala-Val-Leu-Aib-Leu-Leu-Gln-Ser-Leuol
**95**	$C_{55}H_{100}N_{11}O_{14}$	1138.7465	1.7	9.0	Isovaleryl-Aib-Ser-Val-Leu-Aib-Leu-Leu-Gln-Ala-Leuol-Ac
**96**	$C_{57}H_{102}N_{11}O_{16}$	1196.7513	1.0	9.2	Isovaleryl-Aib-Ser-Val-Leu-Aib-Leu-Leu-Gln-Ser(Ac)-Leuol-Ac
**97**	$C_{57}H_{104}N_{11}O_{15}$	1182.7725	1.5	9.3	Isovaleryl-Iva-Ser-Ala-Leu-Aib-Leu-Leu-Gln-Ser-Leuol-Isovaleryl
**98**	$C_{57}H_{104}N_{11}O_{15}$	1182.7742	2.9	9.6	Isovaleryl-Iva-Ser-Leu/Ile-Leu-Aib-Leu-Leu-Gln-Ser(Ac)-Leuol
**99**	$C_{56}H_{101}N_{10}O_{16}$	1169.7417	2.2	9.6	Isovaleryl-Iva-Ser-Val-Leu-Aib-Leu-Leu-Glu-Ser-Leuol-Ac
**100**	$C_{56}H_{102}N_{10}O_{14}$	1152.7632	2.6	9.7	Isovaleryl-Iva-Ser-Val-Leu-Aib-Leu-Leu-Gln-Ala-Leuol-Ac
**101**	$C_{58}H_{104}N_{11}O_{16}$	1210.7680	1.9	9.9	Isovaleryl-Iva-Ser-Val-Leu-Aib-Leu-Leu-Gln-Ser(Ac)-Leuol-Ac
**102**	$C_{53}H_{98}N_{11}O_{13}$	1096.7364	2.2	10.1	Isovaleryl-Aib-Ala-Val-Leu-Aib-Leu-Leu-Gln-Ser-Leuol

**103**	$C_{57}H_{104}N_{11}O_{15}$	1182.7738	2.5	10.1	Isovaleryl-Iva-Ser-Leu/Ile-Leu-Aib-Leu-Leu-Gln-Ser-Leuol-Ac
**104**	$C_{57}H_{104}N_{11}O_{15}$	1182.7735	2.3	10.4	Isovaleryl-Aib-Ser-Aib-Leu-Aib-Leu-Leu-Gln-Ser-Leuol-Isovaleryl
**105**	$C_{55}H_{100}N_{11}O_{14}$	1138.7474	2.4	10.8	Isovaleryl-Aib-Ala-Val-Leu-Aib-Leu-Leu-Gln-Ser(Ac)-Leuol
**106**	$C_{59}H_{106}N_{11}O_{16}$	1224.7834	1.7	11.0	Isovaleryl-Iva-Ser-Leu/Ile-Leu-Aib-Leu-Leu-Gln-Ser(Ac)-Leuol-Ac
**107**	$C_{59}H_{106}N_{11}O_{16}$	1224.7834	1.8	11.3	Isovaleryl-Aib-Ser-Aib-Leu-Aib-Leu-Leu-Gln-Ser(Ac)-Leuol-Isovaleryl
**108**	$C_{55}H_{100}N_{11}O_{14}$	1138.7477	2.7	11.4	Isovaleryl-Aib-Ala-Val-Leu-Aib-Leu-Leu-Gln-Ser-Leuol-Ac
**109**	$C_{56}H_{102}N_{10}O_{14}$	1152.7632	2.6	11.8	Isovaleryl-Iva-Ala-Val-Leu-Aib-Leu-Leu-Gln-Ser(Ac)-Leuol
**110**	$C_{63}H_{112}N_{11}O_{18}$	1310.8210	1.8	11.8	Isovaleryl-Iva-Ser(Succinyl)-Val-Leu-Aib-Leu-Leu-Gln-Ser-Leuol-Isovaleryl
**111**	$C_{58}H_{106}N_{11}O_{15}$	1196.7885	1.7	12.1	Isovaleryl-Aib-Ser-Val-Leu-Aib-Leu-Leu-Gln-Ser-Leuol-Isovaleryl
**112**	$C_{56}H_{102}N_{10}O_{14}$	1152.7635	2.8	12.3	Isovaleryl-Iva-Ala-Val-Leu-Aib-Leu-Leu-Gln-Ser-Leuol-Ac
**113**	$C_{58}H_{106}N_{11}O_{14}$	1180.7948	2.8	12.8	Isovaleryl-Aib-Ser-Val-Leu-Aib-Leu-Leu-Gln-Ala-Leuol-Isovaleryl
**Roseabol C (2)**	$\mathbf{C_{59}H_{108}N_{11}O_{15}}$	**1210.8039**	**1.4**	**13.0**	**Isovaleryl-Iva-Ser-Val-Leu-Aib-Leu-Leu-Gln-Ser-Leuol-Isovaleryl**
**114**	$C_{60}H_{108}N_{11}O_{16}$	1238.7989	1.5	13.1	Isovaleryl-Aib-Ser-Val-Leu-Aib-Leu-Leu-Gln-Ser(Ac)-Leuol-Isovaleryl
**115**	$C_{60}H_{110}N_{11}O_{15}$	1224.8207	2.4	13.1	Isovaleryl-Iva-Ser-Val-Leu-Val/Iva-Leu-Leu-Gln-Ser-Leuol-Isovaleryl
**116**	$C_{59}H_{108}N_{11}O_{14}$	1194.8088	1.4	13.7	Isovaleryl-Iva-Ser-Val-Leu-Aib-Leu-Leu-Gln-Ala-Leuol-Isovaleryl
**117**	$C_{60}H_{107}N_{10}O_{17}$	1239.7842	2.6	13.8	Isovaleryl-Aib-Ser-Val-Leu-Aib-Leu-Leu-Glu-Ser(Ac)-Leuol-Isovaleryl
**118**	$C_{61}H_{110}N_{11}O_{16}$	1252.8148	1.5	14.0	Isovaleryl-Iva-Ser-Val-Leu-Aib-Leu-Leu-Gln-Ser(Ac)-Leuol-Isovaleryl
**119**	$C_{60}H_{110}N_{11}O_{15}$	1224.8209	2.6	14.2	Isovaleryl-Iva-Ser-Ile/Leu-Leu-Aib-Leu-Leu-Gln-Ser-Leuol-Isovaleryl
**120**	$C_{60}H_{110}N_{11}O_{14}$	1208.8250	1.8	15.1	Isovaleryl-Iva-Ser-Ile/Leu-Leu-Aib-Leu-Leu-Gln-Ala-Leuol-Isovaleryl
**121**	$C_{62}H_{112}N_{11}O_{16}$	1266.8301	1.4	15.4	Isovaleryl-Iva-Ser-Ile/Leu-Leu-Aib-Leu-Leu-Gln-Ser(Ac)-Leuol-Isovaleryl
**122**	$C_{58}H_{106}N_{11}O_{14}$	1180.7953	3.2	15.9	Isovaleryl-Aib-Ala-Val-Leu-Aib-Leu-Leu-Gln-Ser-Leuol-Isovaleryl
**123**	$C_{63}H_{114}N_{11}O_{16}$	1280.8475	2.8	16.4	Isovaleryl-Aib-Ser-Val-Leu-Aib-Leu-Leu-Gln-Ser(Isovaleryl)-Leuol-Isovaleryl
**124**	$C_{60}H_{108}N_{11}O_{15}$	1222.8042	1.7	16.8	Isovaleryl-Aib-Ala-Val-Leu-Aib-Leu-Leu-Gln-Ser(Ac)-Leuol-Isovaleryl
**125**	$C_{59}H_{108}N_{11}O_{14}$	1194.8089	1.4	17.0	Isovaleryl-Iva-Ala-Val-Leu-Aib-Leu-Leu-Gln-Ser-Leuol-Isovaleryl
**126**	$C_{64}H_{116}N_{11}O_{16}$	1294.8620	1.8	17.3	Isovaleryl-Iva-Ser-Val-Leu-Aib-Leu-Leu-Gln-Ser(Isovaleryl)-Leuol-Isovaleryl
**127**	$C_{61}H_{110}N_{11}O_{15}$	1236.8196	1.5	17.8	Isovaleryl-Iva-Ala-Val-Leu-Aib-Leu-Leu-Gln-Ser(Ac)-Leuol-Isovaleryl

**128**	$C_{60}H_{109}N_{10}O_{16}$	1225.8047	2.4	18.1	Isovaleryl-Iva-Ser-Val-Leu-Aib-Leu-Leu-Glu(OMe)-Ser-Leuol-Isovaleryl
**129**	$C_{59}H_{108}N_{11}O_{13}$	1178.8137	1.2	18.2	Isovaleryl-Iva-Ala-Val-Leu-Aib-Leu-Leu-Gln-Ala-Leuol-Isovaleryl
**130**	$C_{62}H_{112}N_{11}O_{15}$	1250.8364	2.4	18.2	Isovaleryl-Iva-Ala-Val-Leu-Val/Iva-Leu-Leu-Gln-Ser(Ac)-Leuol-Isovaleryl
**131**	$C_{62}H_{112}N_{11}O_{15}$	1267.8160	2.9	18.3	Isovaleryl-Iva-Ser-Val-Leu-Aib-Leu-Leu-Glu(OMe)-Ser(Ac)-Leuol-Isovaleryl
**132**	$C_{60}H_{110}N_{11}O_{14}$	1208.8262	2.8	18.4	Isovaleryl-Iva-Ala-Leu/Ile-Leu-Aib-Leu-Leu-Gln-Ser-Leuol-Isovaleryl
**133**	$C_{65}H_{118}N_{11}O_{16}$	1308.8778	2.0	18.8	Isovaleryl-Iva-Ser-Leu/Ile-Leu-Aib-Leu-Leu-Gln-Ser(Isovaleryl)-Leuol-Isovaleryl
**134**	$C_{62}H_{112}N_{11}O_{15}$	1250.8351	1.4	19.3	Isovaleryl-Iva-Ala-Leu/Ile-Leu-Aib-Leu-Leu-Gln-Ser(Ac)-Leuol-Isovaleryl
**135**	$C_{63}H_{114}N_{11}O_{15}$	1264.8513	1.8	20.4	Isovaleryl-Aib-Ala-Val-Leu-Aib-Leu-Leu-Gln-Ser(Isovaleryl)-Leuol-Isovaleryl
**136**	$C_{66}H_{118}N_{11}O_{17}$	1336.8728	2.0	21.0	Isovaleryl-Iva-Ser(Ac)-Val-Leu-Aib-Leu-Leu-Gln-Ser(Isovaleryl)-Leuol-Isovaleryl
**137**	$C_{65}H_{117}N_{10}O_{17}$	1309.8610	1.3	21.0	Isovaleryl-Iva-Ser-Val-Leu-Aib-Leu-Leu-Glu(OMe)-Ser(Isovaleryl)-Leuol-Isovaleryl
**138**	$C_{64}H_{116}N_{11}O_{15}$	1278.8662	1.2	21.5	Isovaleryl-Iva-Ala-Val-Leu-Aib-Leu-Leu-Gln-Ser(Isovaleryl)-Leuol-Isovaleryl
**139**	$C_{62}H_{111}N_{10}O_{16}$	1251.8208	2.2	21.9	Isovaleryl-Iva-Ala-Val -Leu-Aib-Leu-Leu-Glu(OMe)-Ser(Ac)-Leuol-Isovaleryl
**140**	$C_{65}H_{118}N_{11}O_{15}$	1292.8817	1.1	22.9	Isovaleryl-Iva-Ala-Leu/Ile-Leu-Aib-Leu-Leu-Gln-Ser(Isovaleryl)-Leuol-Isovaleryl

Table S5. ^{1}H and ^{13}C NMR data (600 and 151 MHz, acetone-d$_6$) of roseabol B (**1**).

Position	δ_H[a] (*J* in Hz)	δ_C[a]	Position	δ_H[a] (*J* in Hz)	δ_C[a]
	**IV**			**Leu**	
1	-	n.a	1	-	175.9
2	2.23 m	43.6	2	4.10 ovl	55.6
3	1.93 ovl	26.2	3	1.98 ovl, 1.60 ovl	39.9
4	0.99 ovl	22.7	4	1.90 ovl	25.6
5	0.92 ovl	22.7	5	1.00 ovl	23.8
	**Iva**		6	0.90 ovl	21.3
1	-	178.3	NH	8.04 br d (6.0)	
2	-	60.3		**Leu**	
3	1.92 ovl, 2.26 ovl	27.0	1	-	175.3
4	0.91 t (7.3)	7.7	2	4.22 m	54.7
5	1.48 s	23.0	3	1.86 ovl, 1.66 ovl	40.4
NH	8.14 br s	-	4	1.88 ovl	25.5
	**Ser**		5	0.94 ovl	23.4
1	-	172.9	6	0.88 ovl	21.2
2	4.10 ovl	60.0	NH	7.73 br d (7.8)	
3	3.90 m	61.5		**Gln**	
NH	8.04 br d (6.0)	-	1	-	174.3
	**Val**		2	4.10 ovl	55.6
1	-	172.7	3	2.18 m	27.8
2	3.95 d (7.9)	61.3	4	2.32 ovl, 2.43 m	33.1
3	2.26 ovl	30.1	5	-	174.9
4	1.06 d (6.7)	19.9	2-NH	7.58 br d (5.8)	
5	1.02 d (6.6)	19.5	5-NH$_2$	n.a	
NH	7.80 br d (7.5)	-		**Ser(Ac)**	
	**Leu**		1	-	169.1
1	-	175.0	2	4.60 ovl	53.7
2	4.38 m	52.9	3	4.32 dd (11.0, 3.4), 4.46 dd (11.0, 7.7)	64.4
3	1.81 ovl, 1.68 ovl	40.7	-<u>C</u>OCH$_3$	-	170.9
4	1.61 ovl	25.2	-CO<u>CH$_3$</u>	2.09 s	20.7
5	0.92 ovl	23.7	NH	7.39 br d (7.9)	
6	0.88 ovl	20.9		**Leuol**	
NH	7.39 br d (7.9)	-	1	4.23 ovl	46.9
	**Aib**		2	1.64 ovl, 1.32 ovl	41.1
1	-	175.2	3	1.86 ovl	25.4
2	-	57.4	4	0.91 ovl	24.8
3	1.59 s	24.1	5	0.86 ovl	21.9
4	1.53 s	26.1	6	4.03 dd (10.9, 5.9), 4.00 dd (10.9, 6.3)	66.6
NH	7.90 br s		NH	6.99 br d (9.0)	
	**Hyp**			**IV**	
1	-	175.8	1	-	172.9
2	4.62 ovl	62.9	2	2.27 ovl	45.7
3	2.34 ovl, 1.91 ovl	37.8	3	2.14 m	26.5
4	4.50 m	70.8	4	0.97	22.9
5	3.82 d (12.3), 3.61 dd (12.3, 3.1)	58.2	5	0.95	22.8

[a] ^{1}H and ^{13}C assignments aided by COSY, TOCSY, HSQC, HMBC and HSQC/TOCSY experiments. N.B. All methyl resonances are in the upfield region, exhibiting significant overlap that precludes the unambiguous determination of coupling constants.

Table S6. Selected NH, Hα and Cα NMR assignments (600 MHz and 151 MHz, DMSO-d_6) of roseabol C (**2**).

Residue	NH	Hα	Cα
	δ_H	δ_H	δ_C
Iva	8.59	-	58.8
Ser	8.20	3.98	58.1
Val	7.64	3.70	61.4
Leu	7.44	4.22	50.9
Aib	7.73	-	55.8
Leu	7.54	3.91	53.5
Leu	7.73	4.03	52.6
Gln	7.65	4.12	52.7
Ser	7.44	4.18	55.5
Leu-ol	7.27	4.04	45.0

Assignments were established by analysis of COSY, TOCSY, HSQC, HMBC and HSQC-TOCSY spectra. Due to extensive signal overlap in the aliphatic region, particularly for Leu and isovaleryl residues, only selected and unambiguously assigned resonances are reported.

Table S7. Putative genes flanking the *rsb* gene cluster.

protein	amino acids	proposed function	closest homolog (protein, origin)	identities/ positives (%)	accession #
ORF1	551	transport protein	unnamed protein product [*Clonostachys chloroleuca*]	99/99	CAI6088549.1
ORF2	533	sulfatase	unnamed protein product [*Clonostachys byssicola*]	81/84	CAH0003518.1
ORF3	156		unnamed protein product [*Clonostachys rhizophaga*]	93/95	CAG9945904.1
ORF4	636		unnamed protein product [*Clonostachys byssicola*]	96/97	CAH0003512.1
ORF5	1004	ankyrin repeat domain-containing protein	unnamed protein product [*Clonostachys rhizophaga*]	98/97	CAH0036524.1
ORF6	253	SLATT domain-containing protein	unnamed protein product [*Clonostachys rosea f. rosea IK726*]	94/95	CAG9945900.1
ORF7	204		hypothetical protein V2G26_004385 [*Clonostachys chloroleuca*]	92/93	KAK7216382.1
rsb	13353	PKS-NRPS	unnamed protein product [*Clonostachys rhizophaga*]	97/97	CAH0036536.1
ORF9	1510	ankyrin repeat domain-containing protein	unnamed protein product [*Clonostachys rhizophaga*]	87/91	CAH0036540.1
ORF10	313		unnamed protein product [*Clonostachys rosea f. rosea IK726*]	95/98	CAG9945896.1
ORF11	485	protein kinase	unnamed protein product [*Clonostachys rhizophaga*]	98/98	CAH0036548.1
ORF12	986	FAD-dependent monooxygenase	unnamed protein product [*Clonostachys rosea f. rosea IK726*]	97/98	CAG9945892.1
ORF13	320	hydroxyquinol 1,2-dioxygenase	hypothetical protein V2G26_004373 [*Clonostachys chloroleuca*]	98/99	KAK7216370.1

ORF14	367	asparaginase	unnamed protein product [*Clonostachys rosea*]	93/96	VUC27373.1
ORF15	923	regulatory enzyme	unnamed protein product [*Clonostachys rhizophaga*]	98/98	CAH0036572.1
ORF16	345	NAD-dependent oxidoreductase	unnamed protein product [*Clonostachys rosea f. rosea IK726*]	94/97	CAG9945887.1
ORF17	329	alpha/beta hydrolase	esterase lipase [*Fusarium denticulatum*]	90/95	KAF5695335.1

Table S8. PARAS substrate prediction and binding pocket signatures of adenylation domains from the Rsb synthetase. Predictions not corresponding to aa residues observed in peptaibol variants are highlighted in red.

	PARAS Prediction	Binding pocket signatures (position)[a]									
		235	236	239	278	299	301	322	330	331	517
Rsb_A1	Aib	D	F	C	F	F	A	G	V	V	K
Rsb_A2	Aib/Ala[b]	D	L	G	V	V	A	A	V	I	K
Rsb_A3	Leu[b]	D	A	M	V	I	A	A	V	I	K
Rsb_A4	Leu	D	Q	M	I	L	G	A	V	A	K
Rsb_A5	Aib	D	I	G	W	A	A	G	L	V	K
Rsb_A6	Cys	D	L	F	F	G	G	I	F	W	K
Rsb_A7	Aib	D	L	T	Y	S	G	G	V	H	K
Rsb_A8	Leu	D	F	V	C	L	G	A	V	M	K
Rsb_A9	Gln	D	G	G	M	V	G	G	N	Y	K
Rsb_A10	Ala[b]/Ser	D	I	G	F	L	A	S	I	I	K
Rsb_A11	Leu	D	A	S	V	N	G	A	I	S	K

[a] as reported by Della Sala *et al.* 2020 [1]

[b] residue present in minor congeners

1. Della Sala G, Mangoni A, Costantino V, Teta R. Identification of the Biosynthetic Gene Cluster of Thermoactinoamides and Discovery of New Congeners by Integrated Genome Mining and MS-Based Molecular Networking. *Front Chem*. 2020;8:397. doi:10.3389/fchem.2020.00397.

Table S9. MZmine parameters for MS raw data processing.

A) MZmine parameters for the generation of the molecular network reported in Figure S1.

**Feature detection**	
MS level 1	1.0e5
MS level 2	1.0e3
ADAP chromatogram builder	• minimum group size in # of scans: 5<br>• group intensity threshold: 1.0e5<br>• minimum highest intensity: 1.0e5<br>• *m/z* tolerance: 0.01 *m/z* or 10 ppm
**Chromatogram deconvolution**	
local minimum search	• chromatographic threshold: 10%<br>• search minimum in RT range: 1 min<br>• minimum relative height: 30%<br>• minimum absolute height: 1.0e5<br>• minimum ratio of peak top/edge: 1.2<br>• peak duration: 0-10 min<br>• m/z range for MS^2 scan pairing: 0.01 Da<br>• RT range for MS^2 scan pairing: 0.2 min
**Alignment**	
Join aligner	• *m/z* tolerance: 0.002 *m/z* or 5 ppm<br>• retention time tolerance: 0.2 min
**Identification**	
Adduct search ([M+Na–H], [M+K–H], [M+Mg−2H], [M+NH_3], [M-Na+NH_4], [M+1, ^{13}C])	• retention time tolerance: 0.1 min<br>• *m/z* tolerance: 0.002 *m/z* or 5 ppm<br>• max relative adduct peak height: 100%

B) MZmine parameters for the generation of the molecular networks reported in Figure 1 and S2.

**Feature detection**	
MS level 1	1.0e5
MS level 2	1.0e3
ADAP chromatogram builder	• minimum group size in # of scans: 5<br>• group intensity threshold: 1.0e5<br>• minimum highest intensity: 1.0e5<br>• *m/z* tolerance: 0.01 *m/z* or 10 ppm
**Chromatogram deconvolution**	
local minimum search	• chromatographic threshold: 10%<br>• search minimum in RT range: 0.50 min<br>• minimum relative height: 30%<br>• minimum absolute height: 1.0e5<br>• minimum ratio of peak top/edge: 1.2<br>• peak duration: 0-10 min<br>• m/z range for MS^2 scan pairing: not applied<br>• RT range for MS^2 scan pairing: not applied
**Alignment**	
Join aligner	• *m/z* tolerance: 0.002 *m/z* or 5 ppm<br>• retention time tolerance: 0.2 min
**Identification**	
Adduct search ([M+Na–H], [M+K–H], [M+Mg−2H], [M+NH_3], [M-Na+NH_4], [M+1, ^{13}C])	• retention time tolerance: 0.1 min<br>• *m/z* tolerance: 0.002 *m/z* or 5 ppm<br>• max relative adduct peak height: 100%

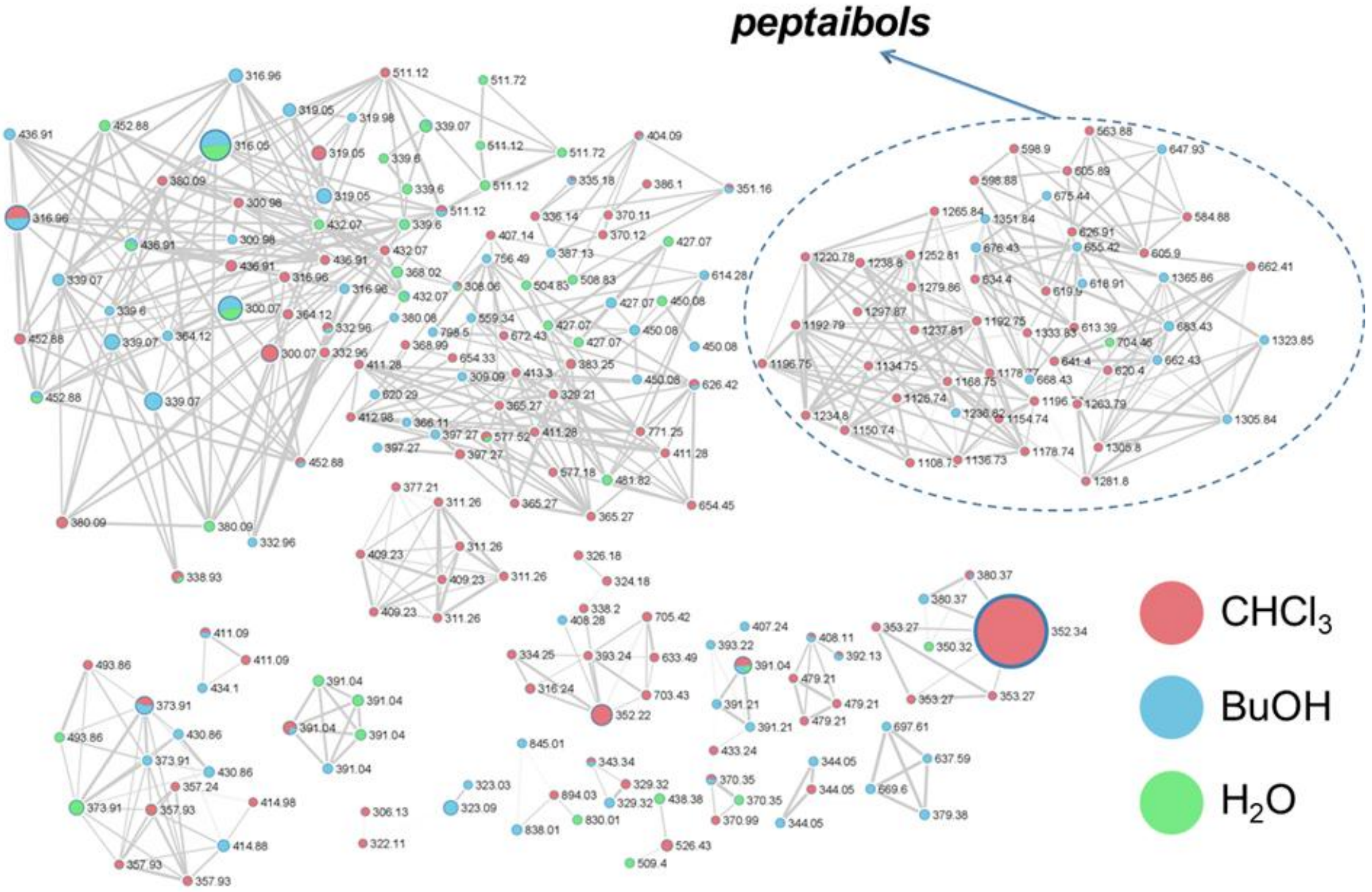

Figure S1. Molecular network of $CHCl_3$, BuOH, and H_2O extracts from *C. rosea* IG119. The network reveals that the peptaibol cluster is predominantly found in the $CHCl_3$ extract. Nodes are visualized as pie charts showing metabolite distribution in the 3 extracts. Node size is relative to the areas of the relevant peaks in the extracted-ion chromatograms from the LC-MS run; edge thickness reflects cosine score similarity, which indicates MS^2 spectra similarity.

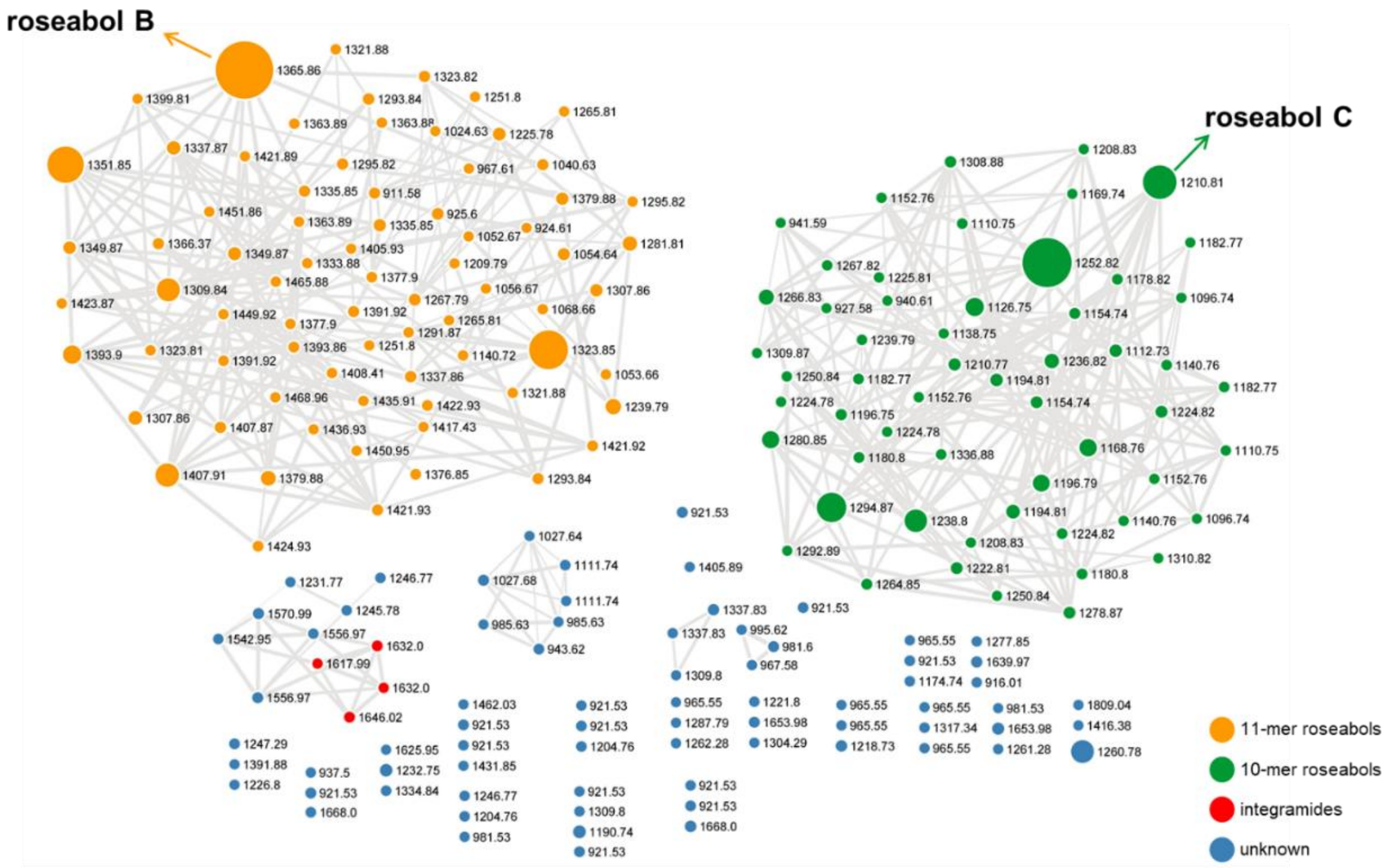

Figure S2. Molecular network of the EtOAc extract from the large-scale fermentation of *C. rosea* IG119 in ME medium (0% salinity, CSL-supplemented). Nodes are coloured according to compound classes, and their size is relative to the areas of the relevant peaks in the extracted-ion chromatograms from the LC-MS run. Edge thickness reflects cosine score similarity which indicates MS^2 spectra similarity.

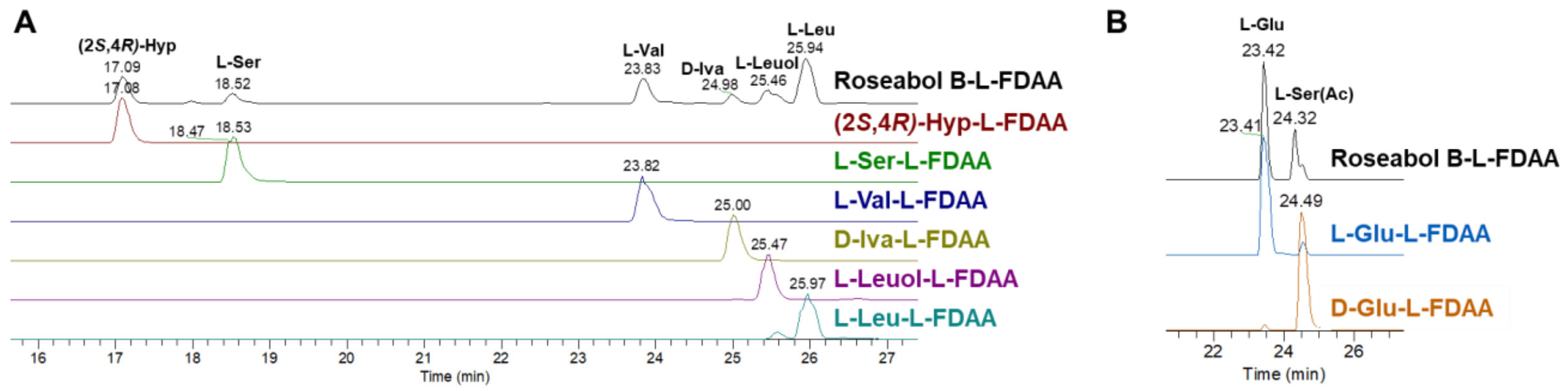

Figure S3. Advanced Marfey's analysis of roseabol B (**1**) using a C18 reversed-phase column. (A) Extracted-ion chromatograms at m/z 384.1150, 358.0993, 370.1357, 370.1721, and 384.1514 of the L-FDAA derivatives from roseabol B hydrolysis and of (2S,4R)-Hyp, L-Ser, L-Val, D-Iva, L-Leuol, and L-Leu L-FDAA derivatives. (B) Extracted-ion chromatograms at m/z 410.1099 of the L-FDAA derivatives from the hydrolysis of roseabol B and of L- and D-Glu L-FDAA derivatives. Intact L-Ser(Ac) was still present after roseabol B (**1**) hydrolysis. L-Ser(Ac) and L-Glu are isobaric amino acids and were unambiguously distinguished based on their different MS/MS spectra.

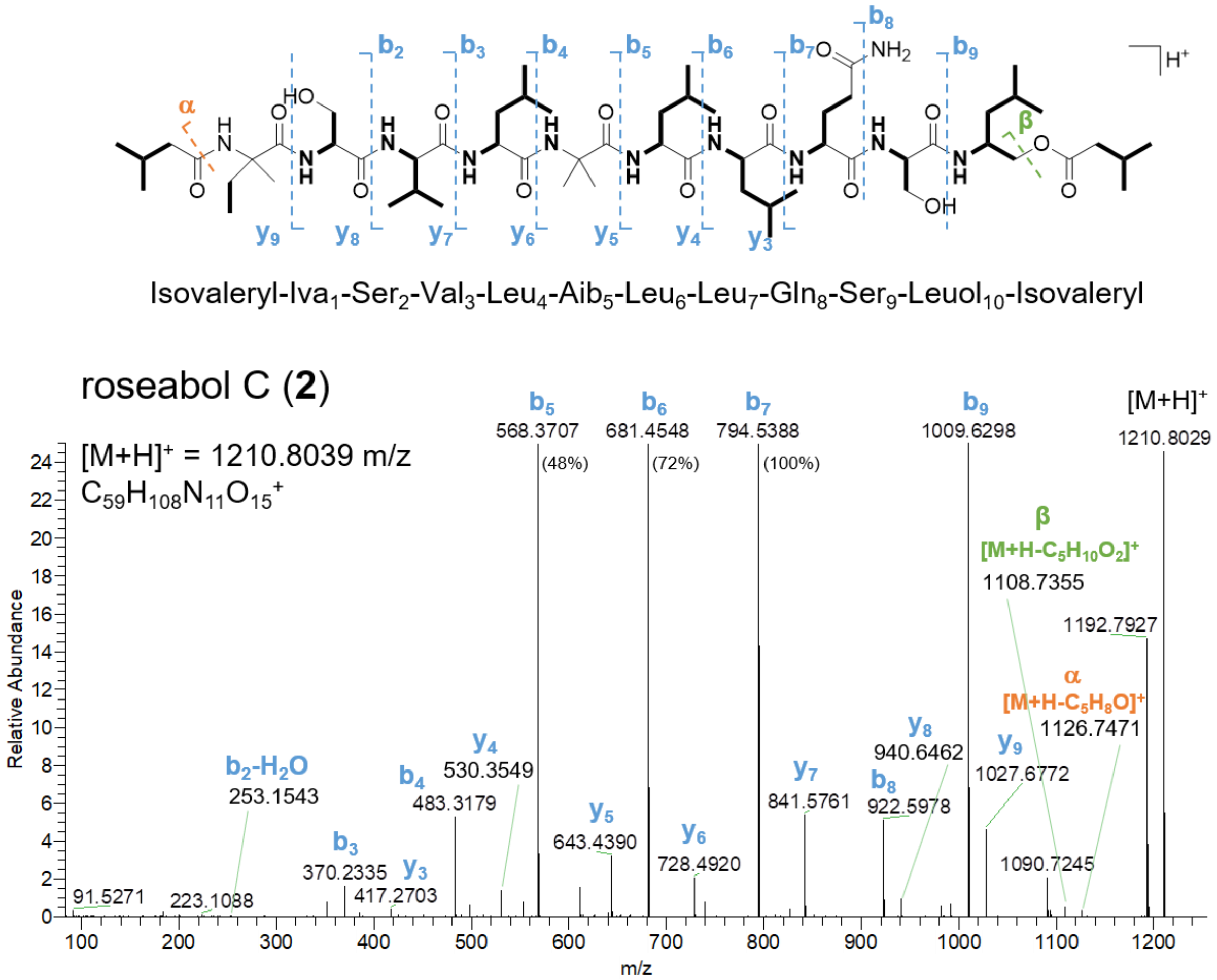

Figure S4. COSY/TOCSY connectivities (in bold) and MS[2] fragmentations of roseabol C (**2**).

Figure S5. ^{1}H NMR (700 MHz, DMSO-d$_6$) of roseabol B **(1)**

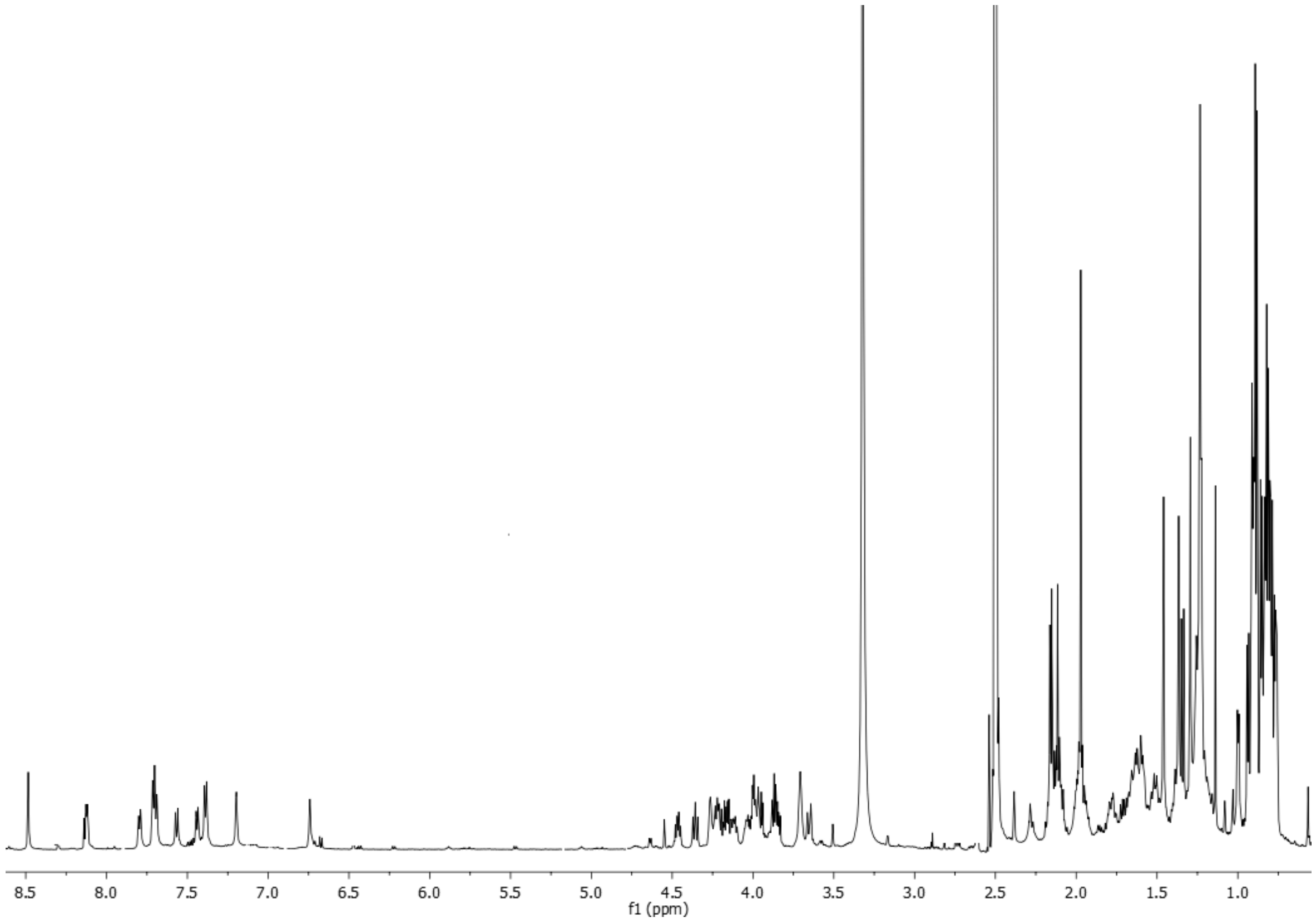

Figure S6. Expanded ^{1}H NMR (700 MHz, DMSO-d$_6$) spectrum of the NH region of roseabol B **(1)**

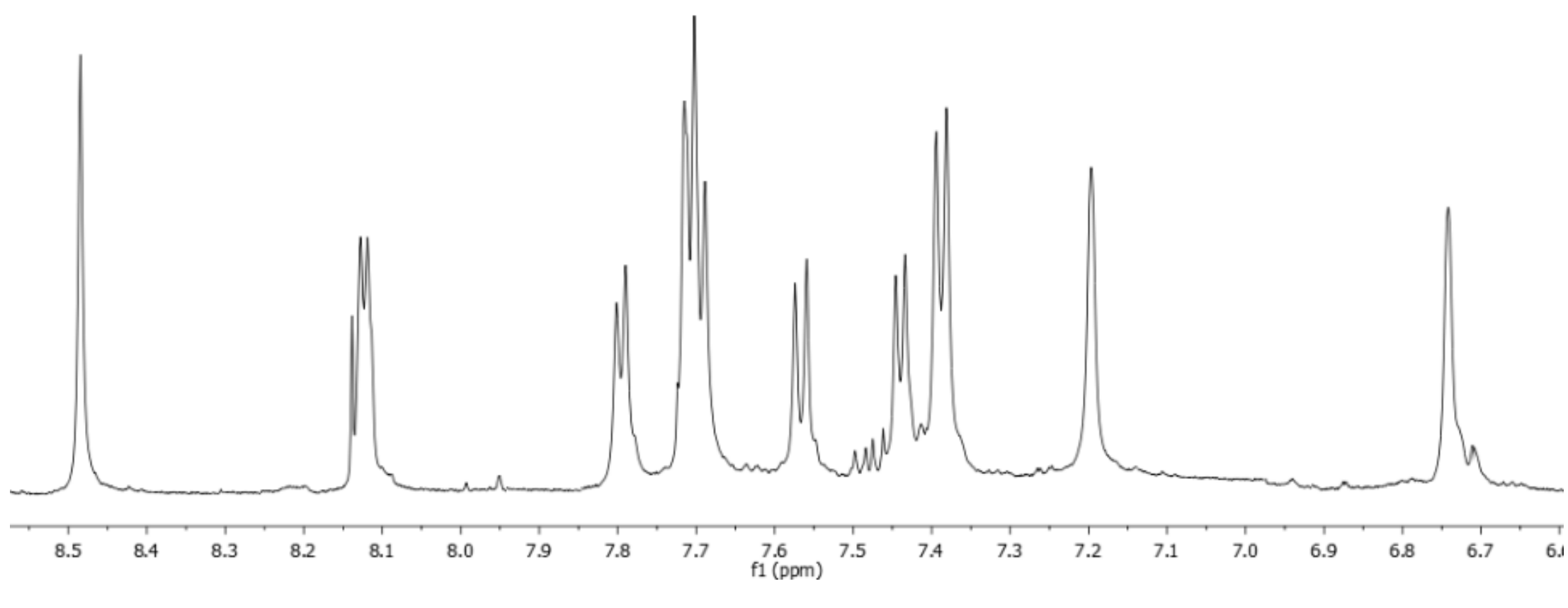

Figure S7. ^{13}C NMR (176 MHz, DMSO-d_6) of roseabol B **(1)**

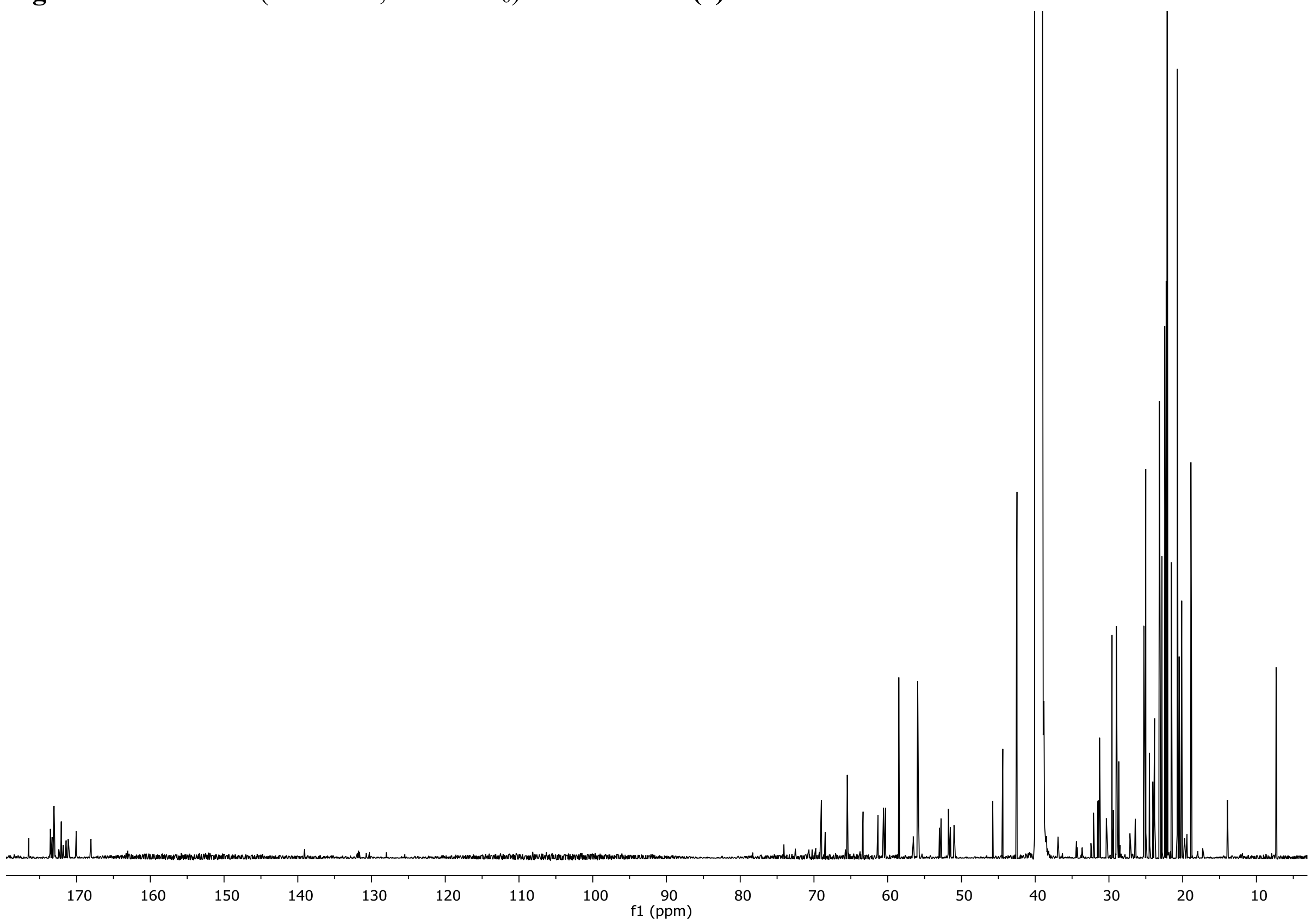

Figure S8. Expanded ^{13}C NMR (176 MHz, DMSO-d_6) spectrum of the carbonyl region of roseabol B (**1**)

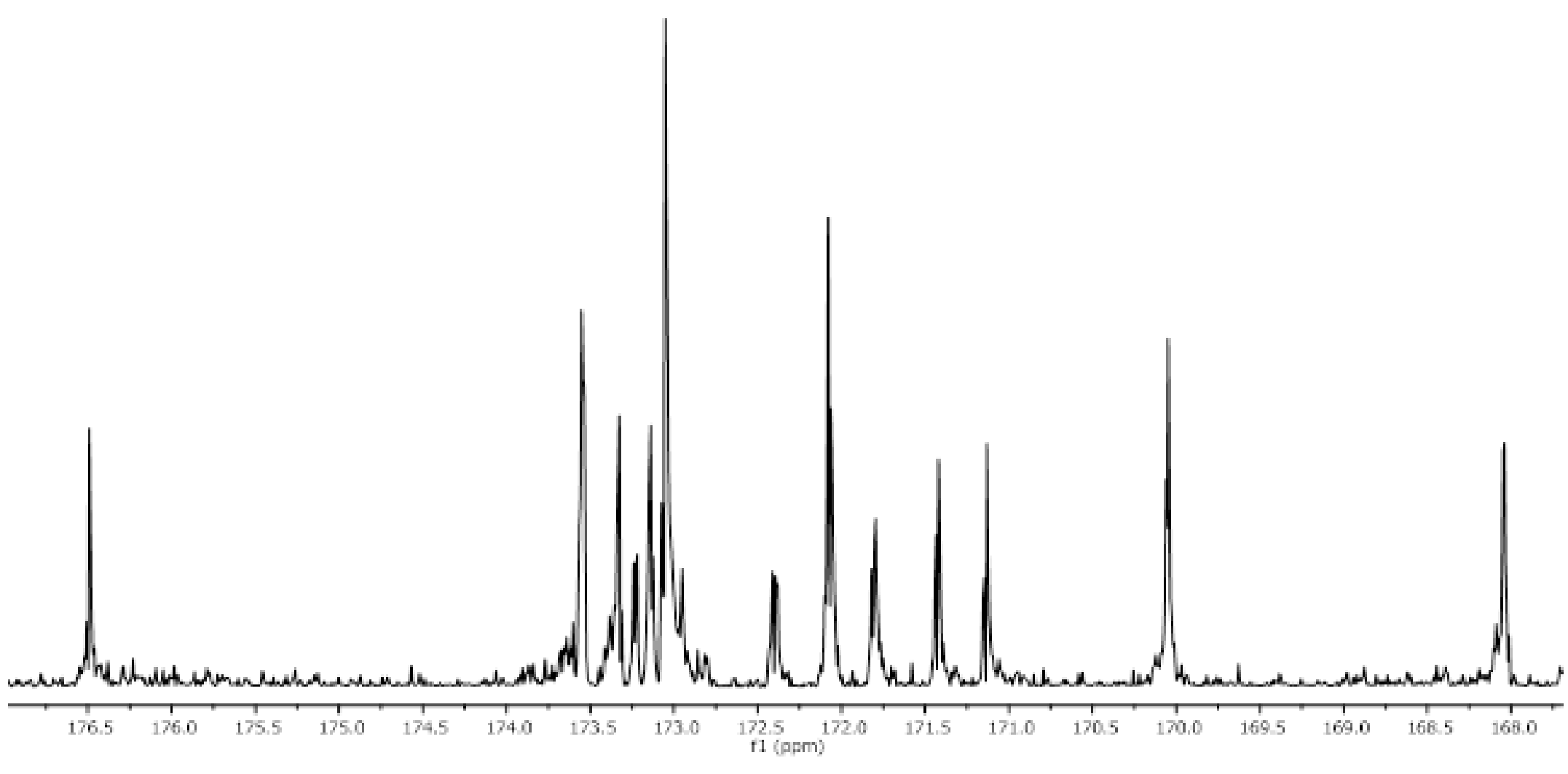

Figure S9. HSQC (700 MHz, DMSO-d_6) of roseabol B **(1)**

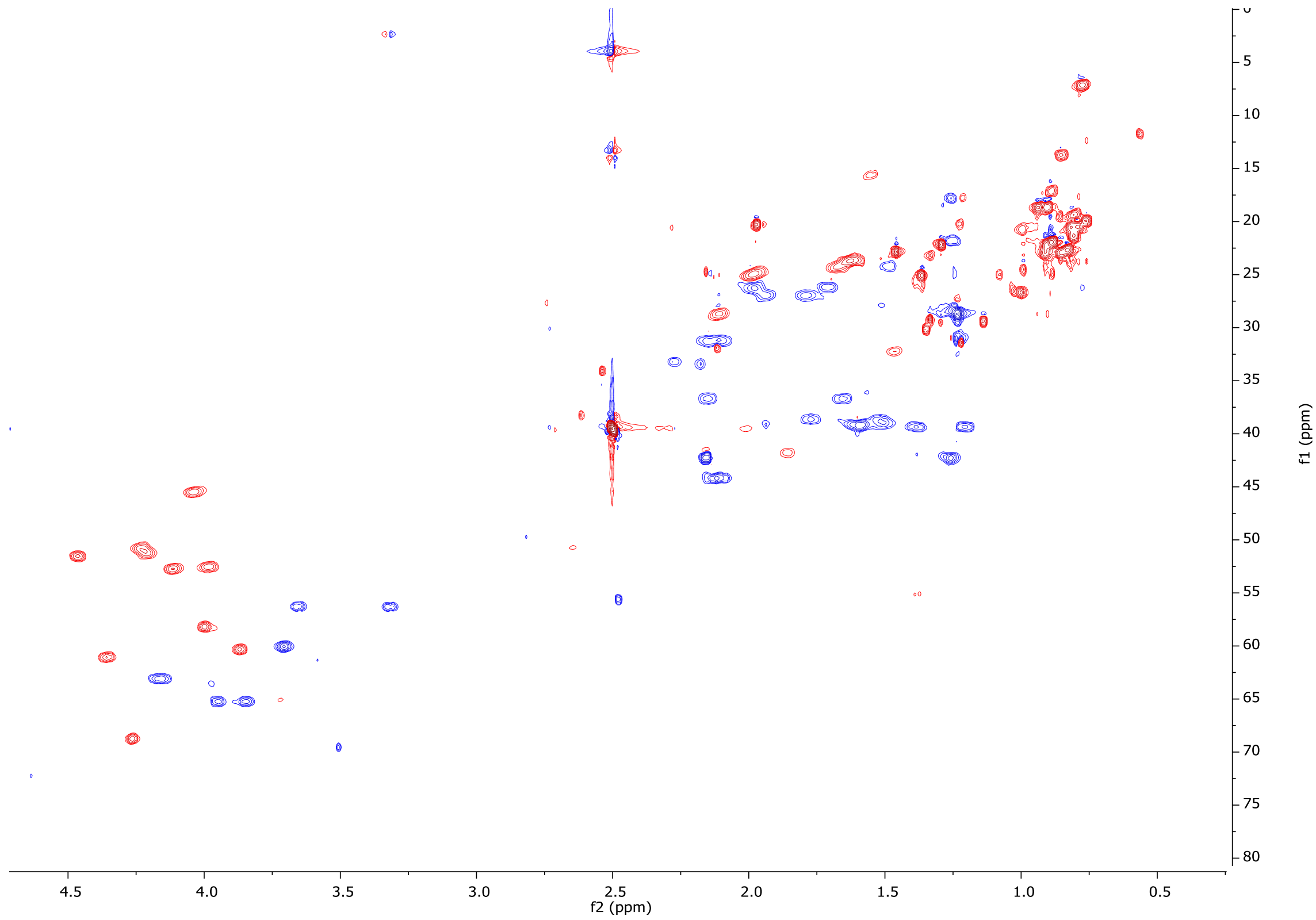

Figure S10. HMBC (700 MHz, DMSO-d_6) of roseabol B **(1)**

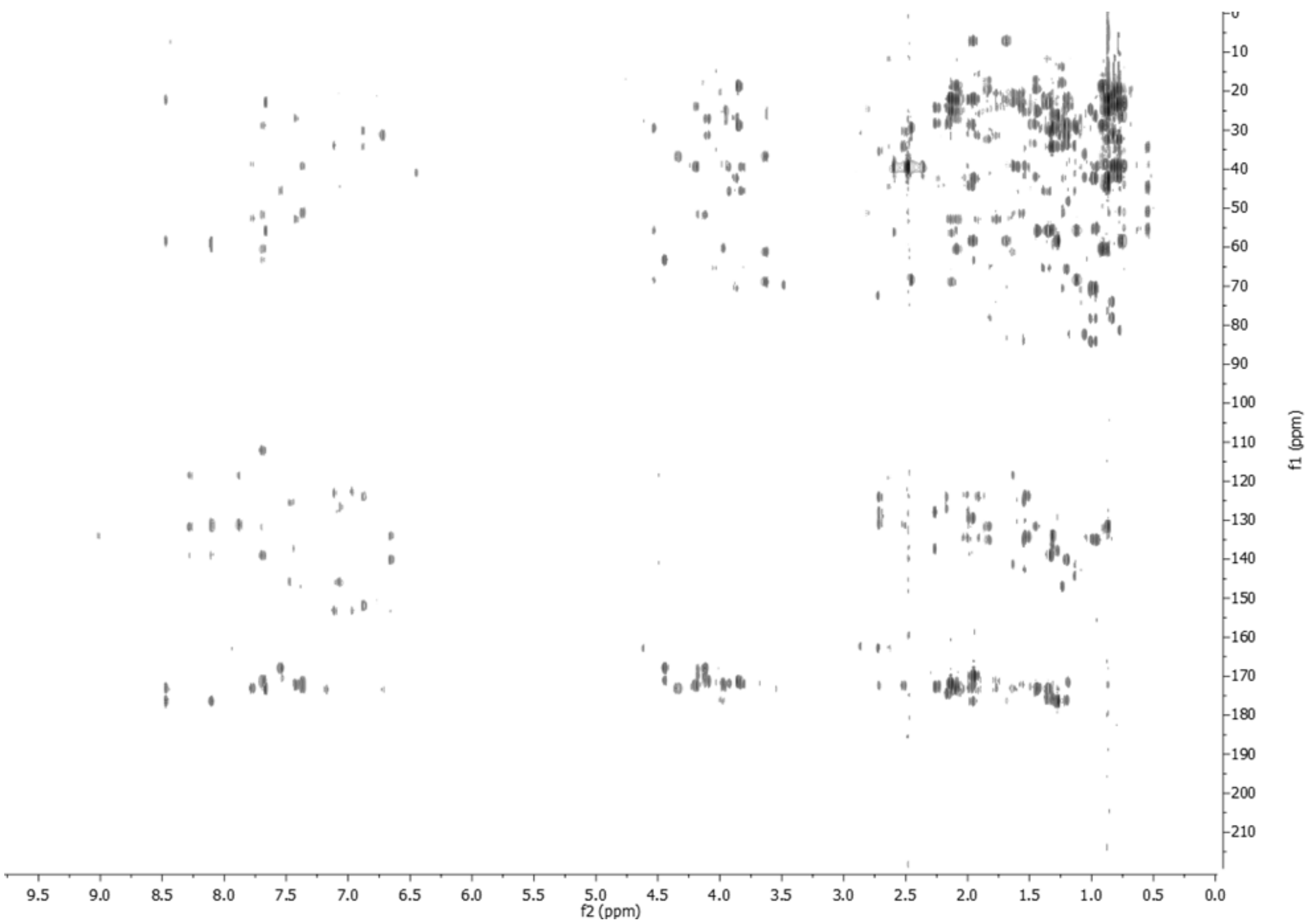

Figure S11. TOCSY (700 MHz, DMSO-d_6) of roseabol B **(1)**

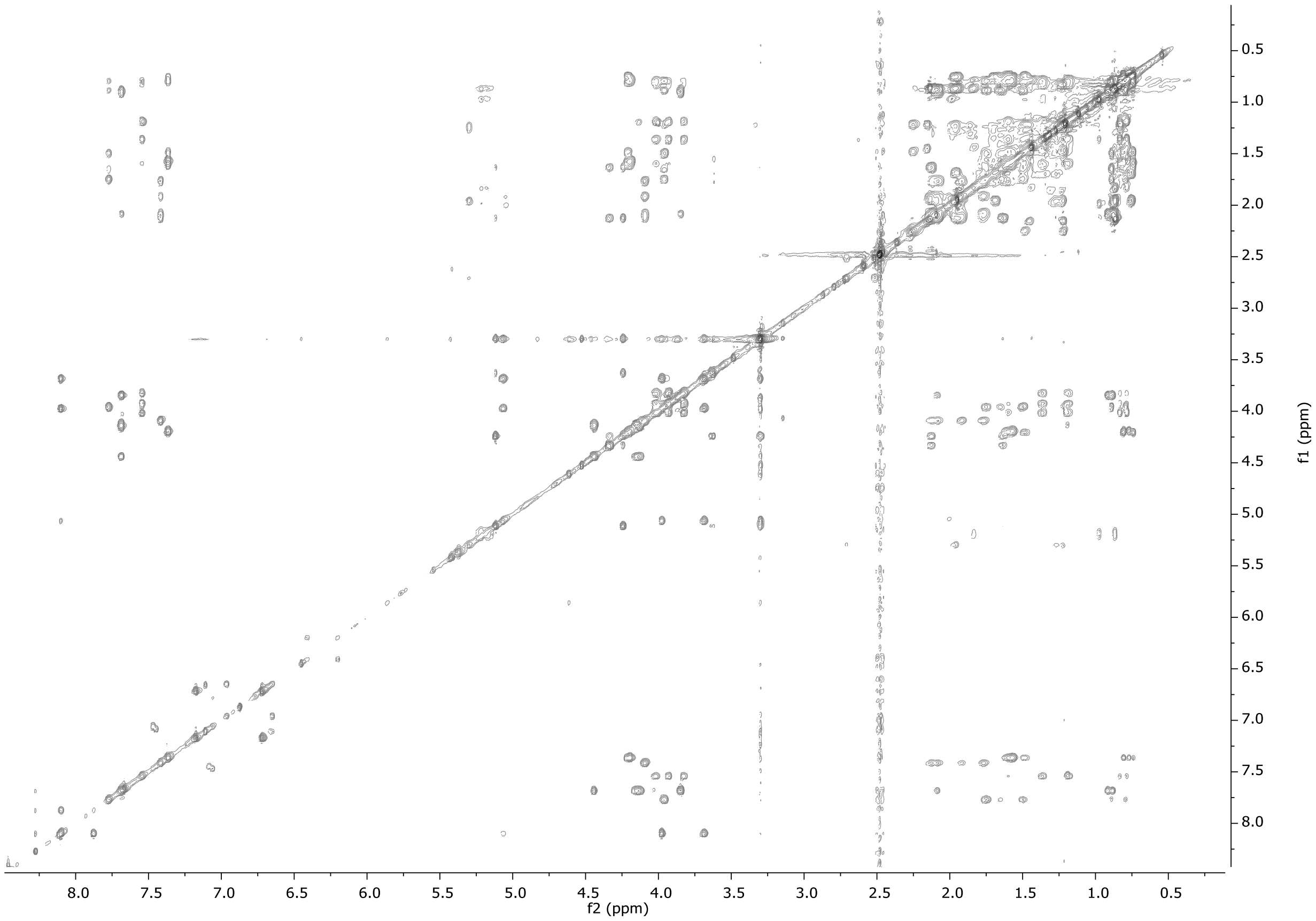

Figure S12. COSY (700 MHz, DMSO-d_6) of roseabol B **(1)**

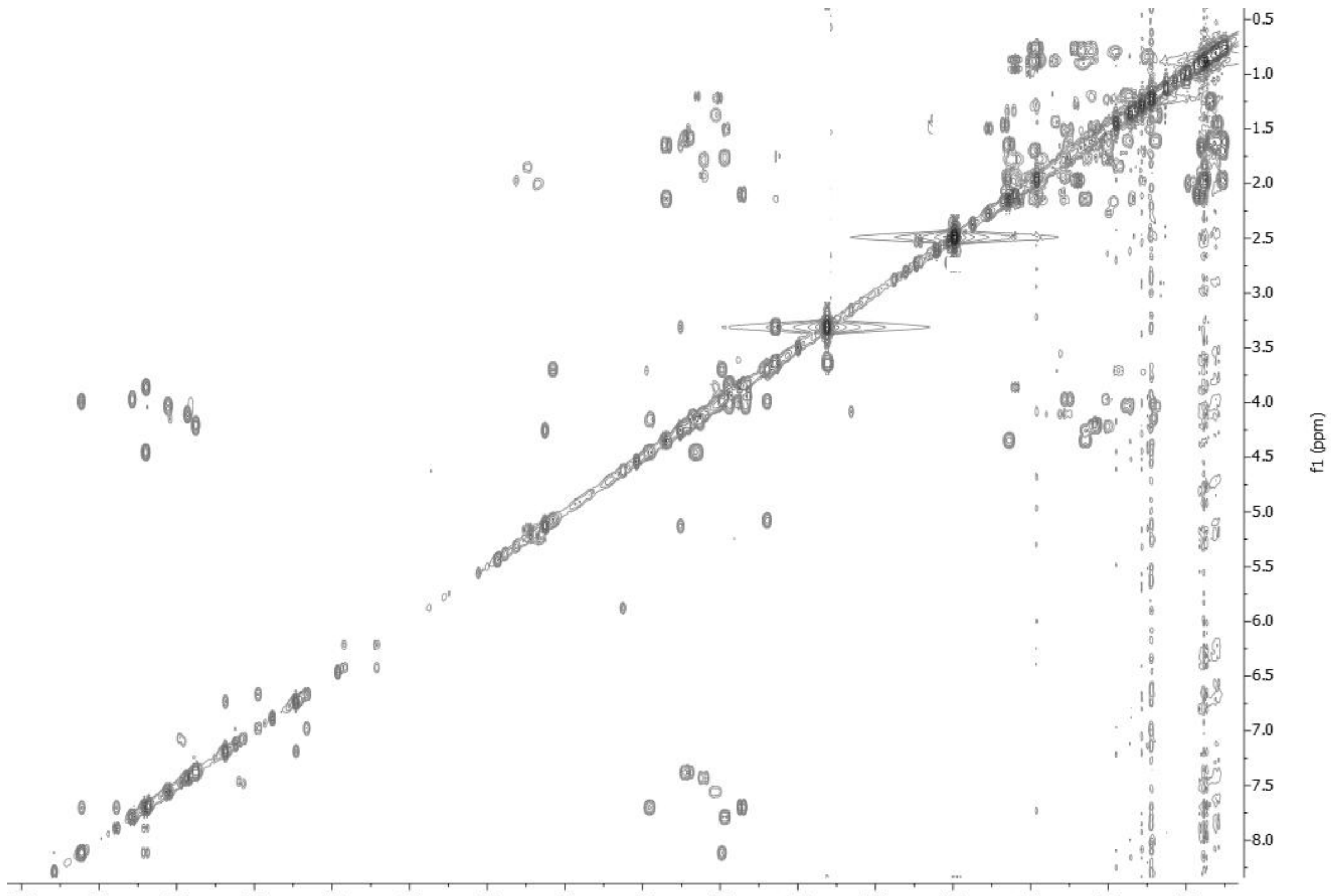

Figure S13. HSQC/TOCSY (700 MHz, DMSO-d_6) of roseabol B **(1)**

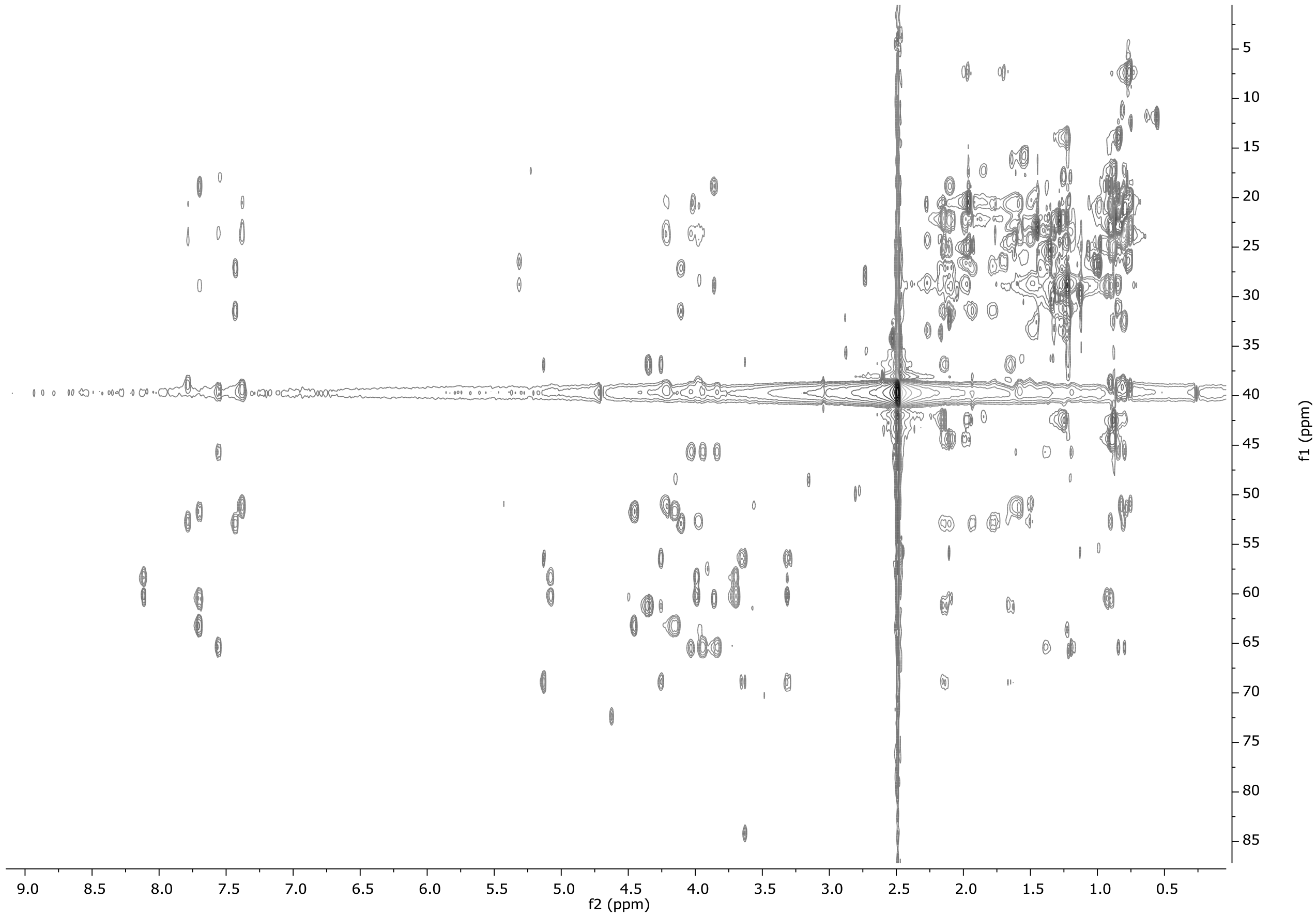

Figure S14. NOESY (700 MHz, DMSO-d_6) of roseabol B **(1)**

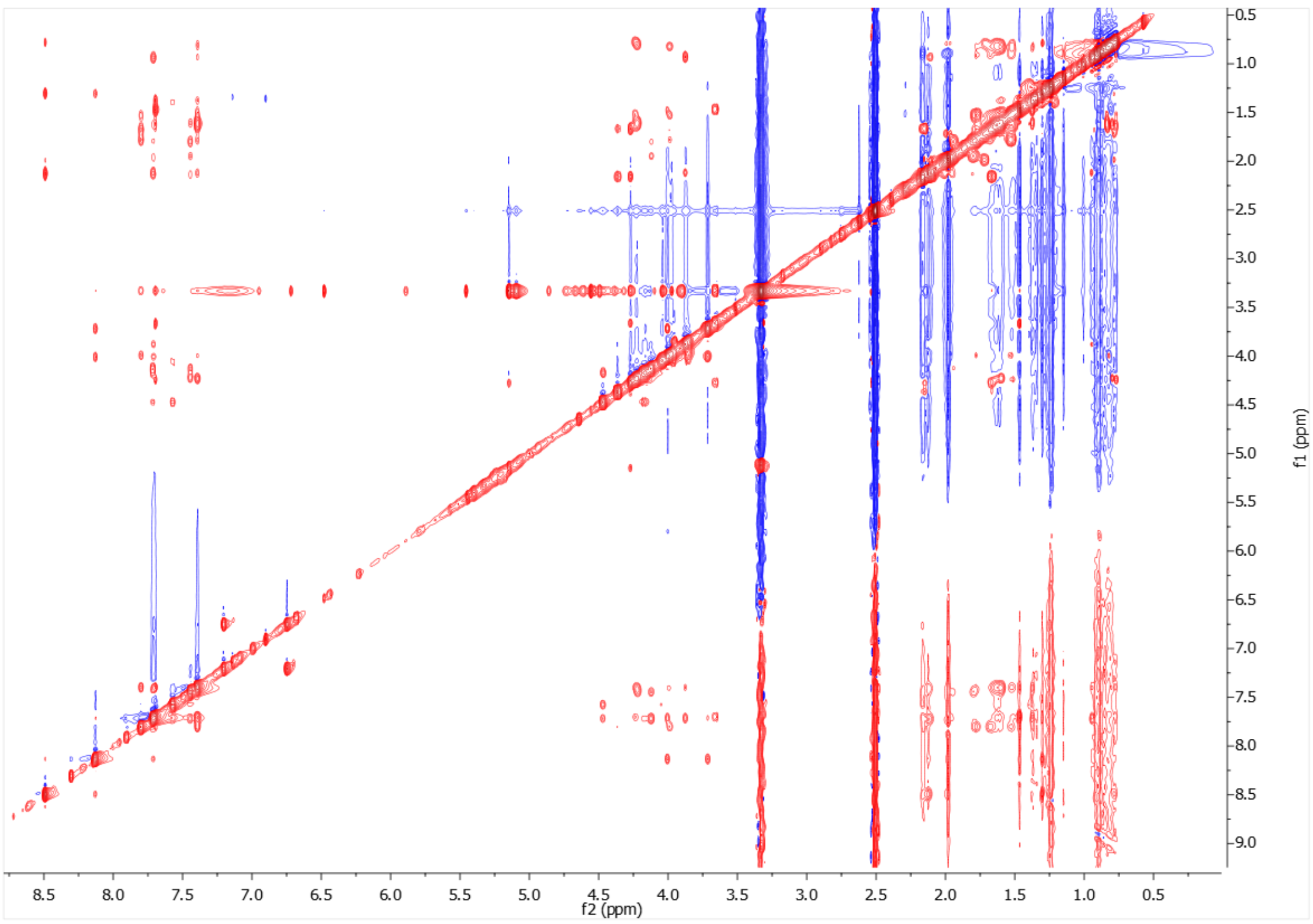

Figure S15. ^{1}H NMR (600 MHz, acetone-d$_6$) of roseabol B (**1**)

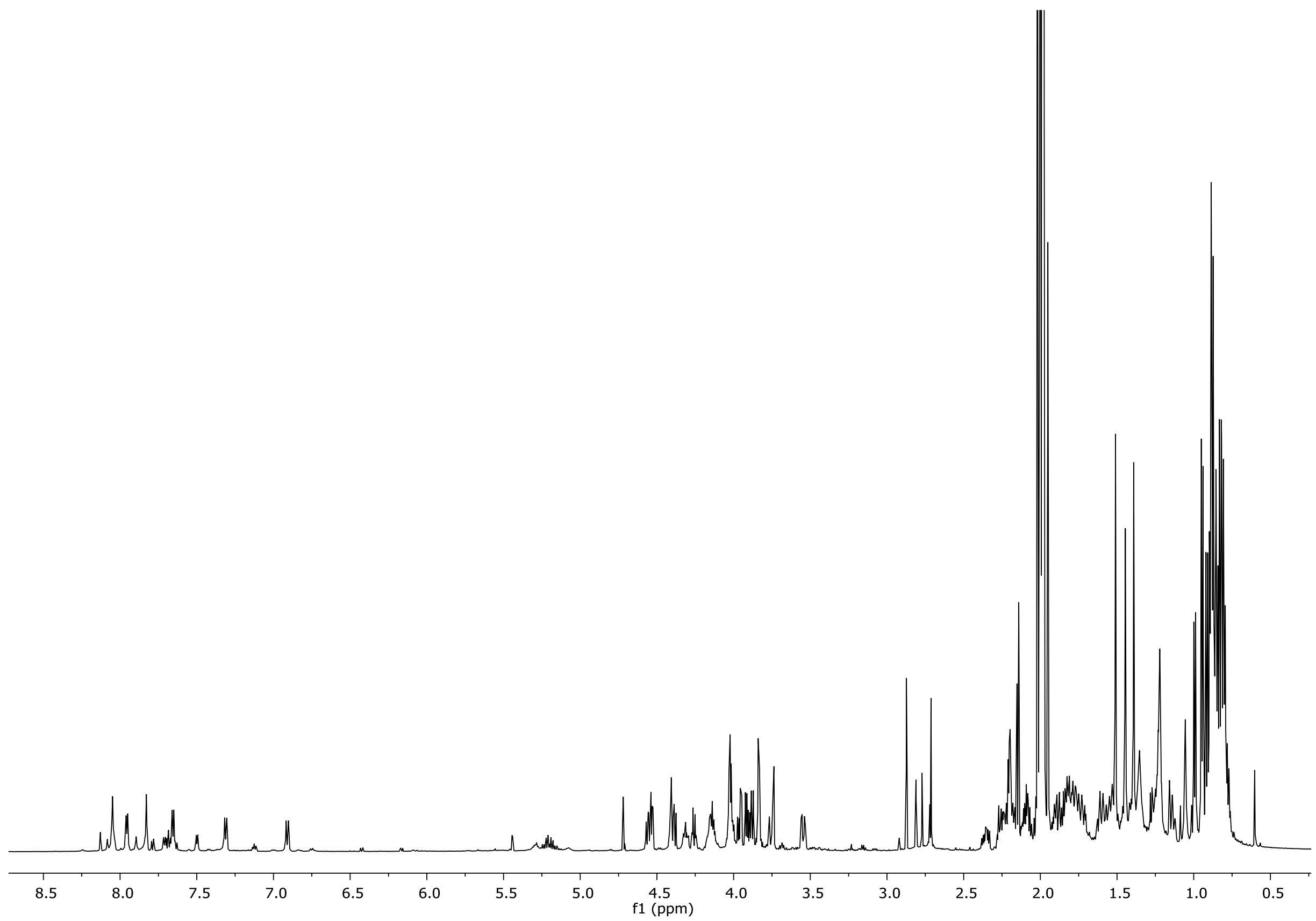

Figure S16. ^{13}C NMR (151 MHz, acetone-d$_6$) of roseabol B (**1**)

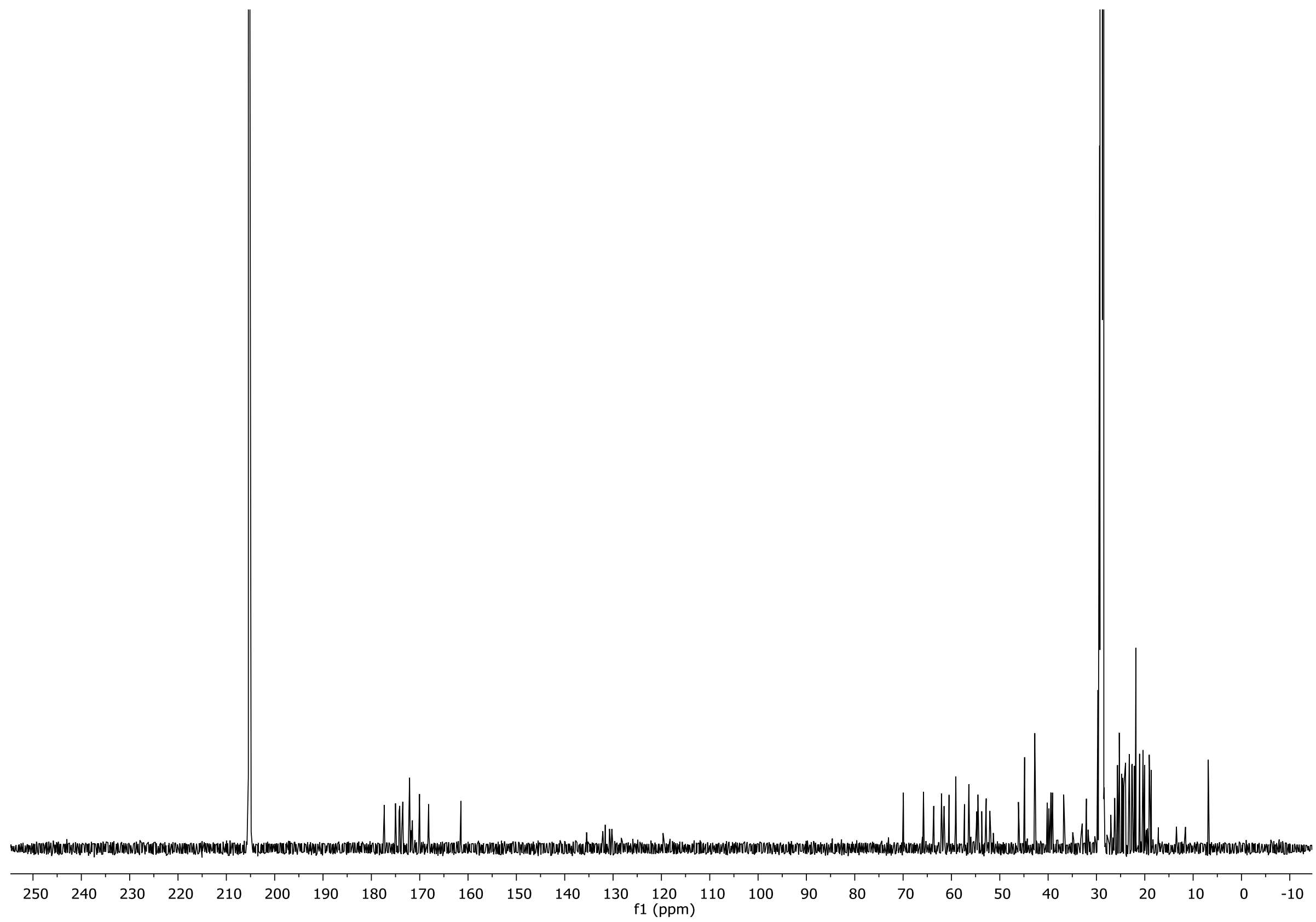

Figure S17. HSQC (600 MHz, acetone-d$_6$) of roseabol B (**1**)

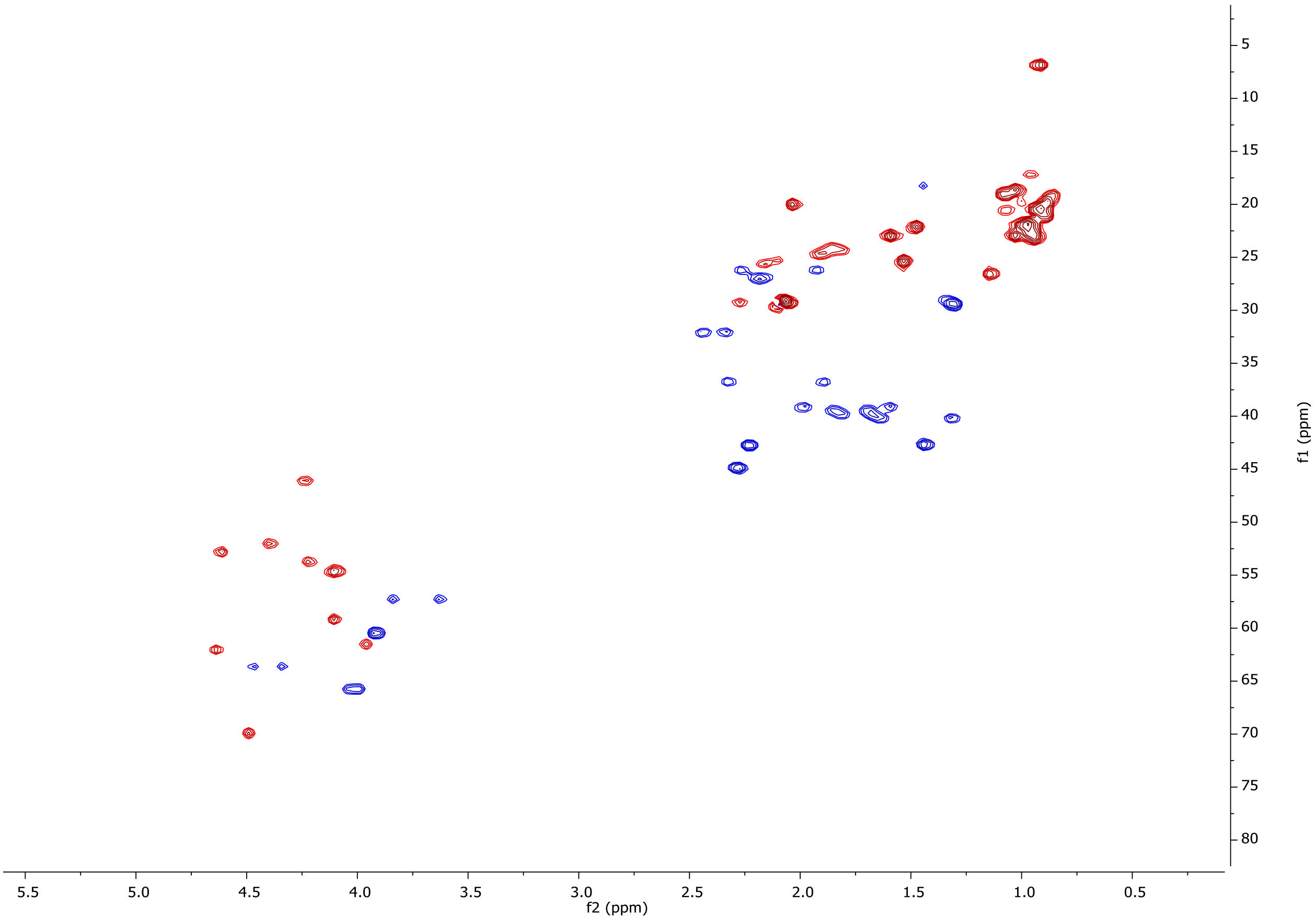

Figure S18. HMBC (600 MHz, acetone-d$_6$) of roseabol B (**1**)

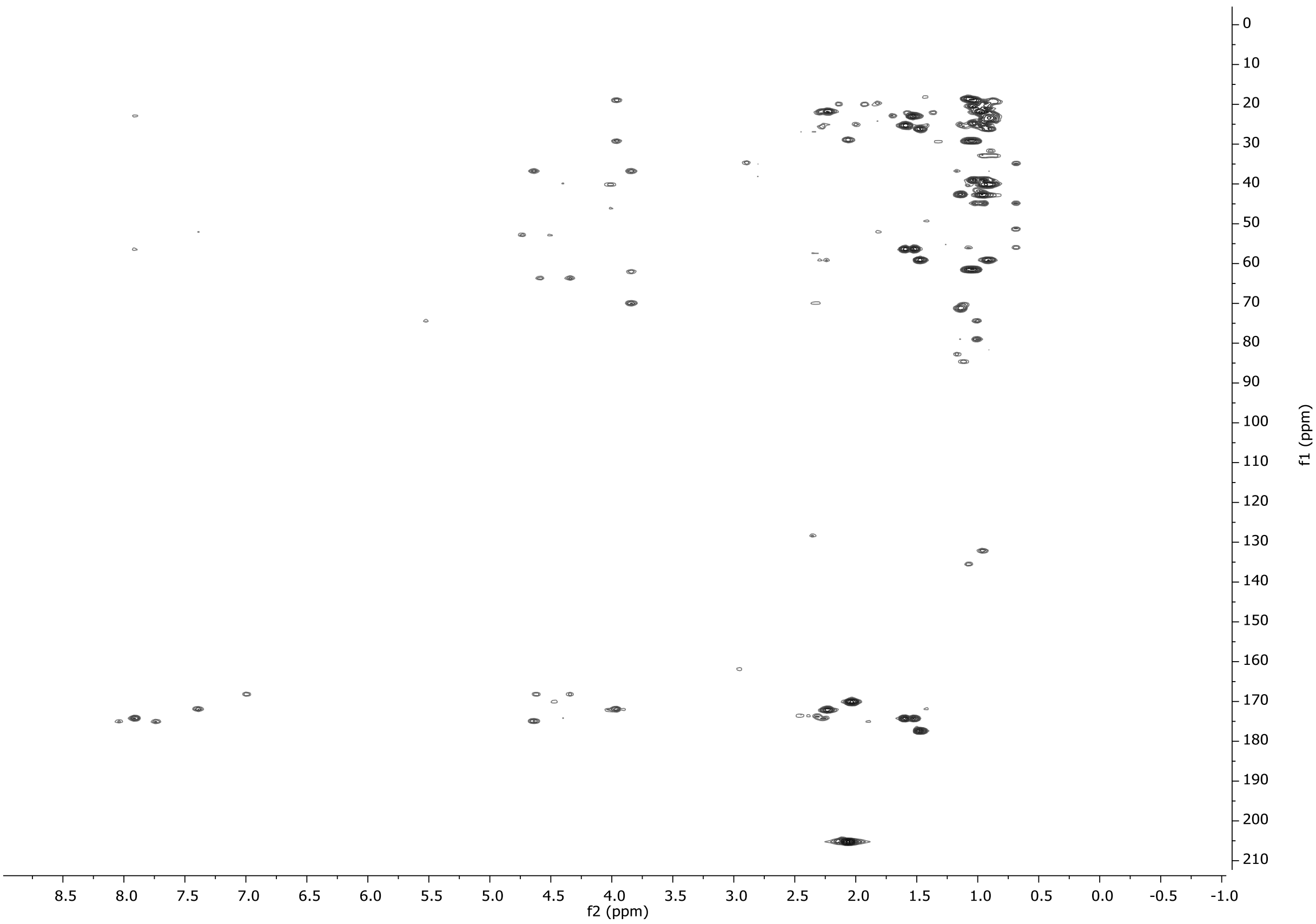

Figure S19. TOCSY (600 MHz, acetone-d_6) of roseabol B (**1**)

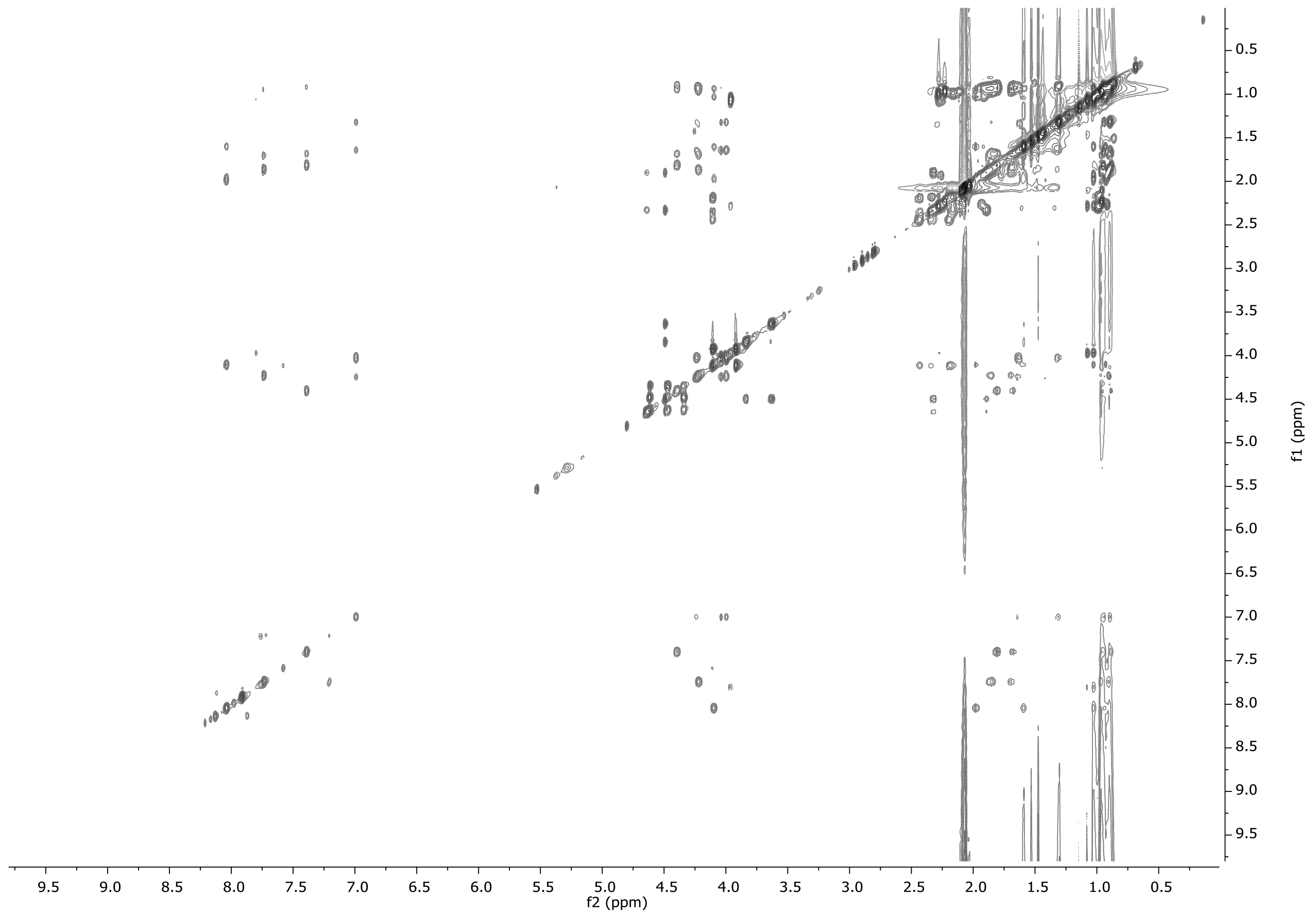

Figure S20. COSY (600 MHz, acetone-d_6) of roseabol B (**1**)

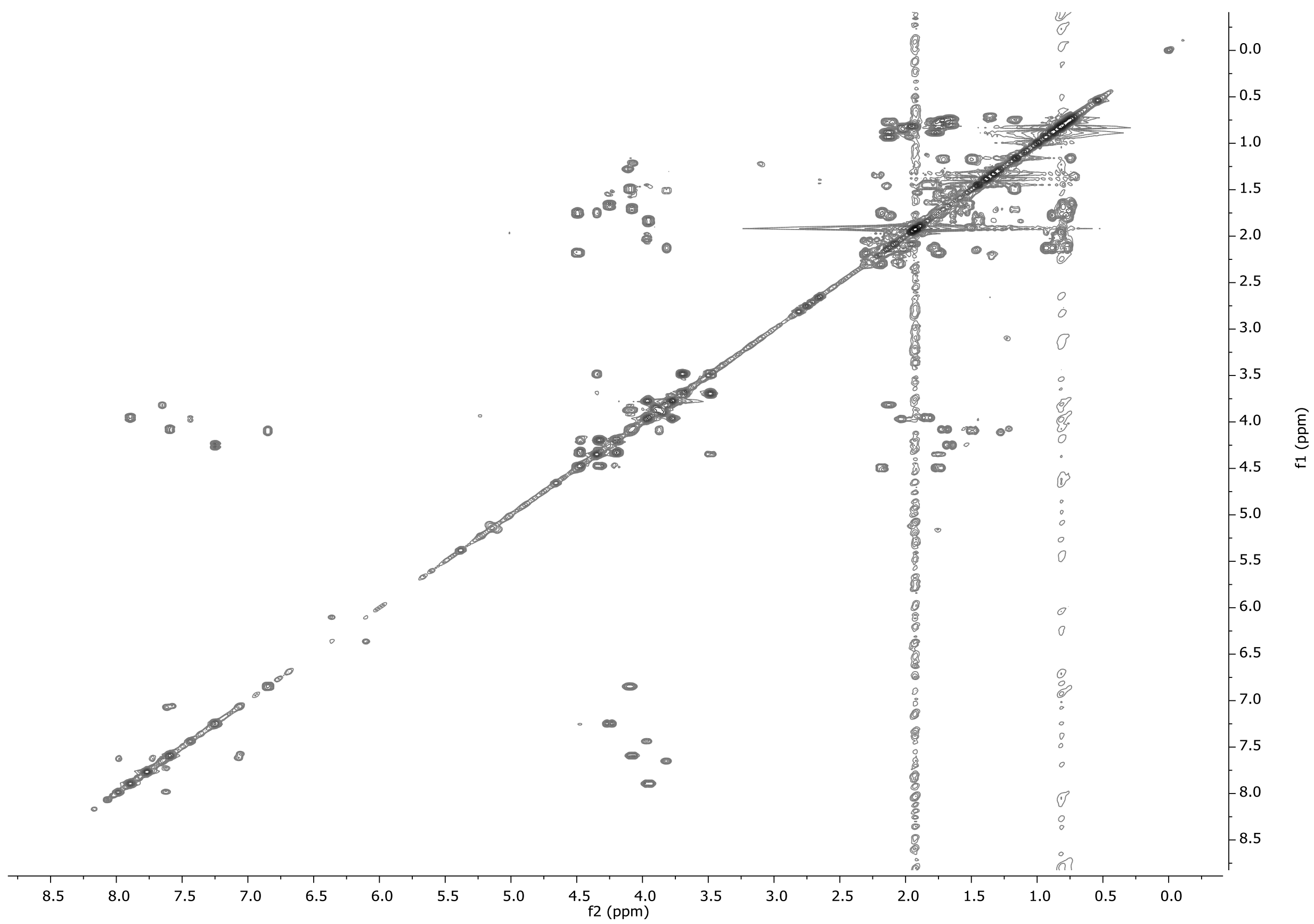

Figure S21. HSQC/TOCSY (600 MHz, DMSO-d_6) of roseabol B (**1**)

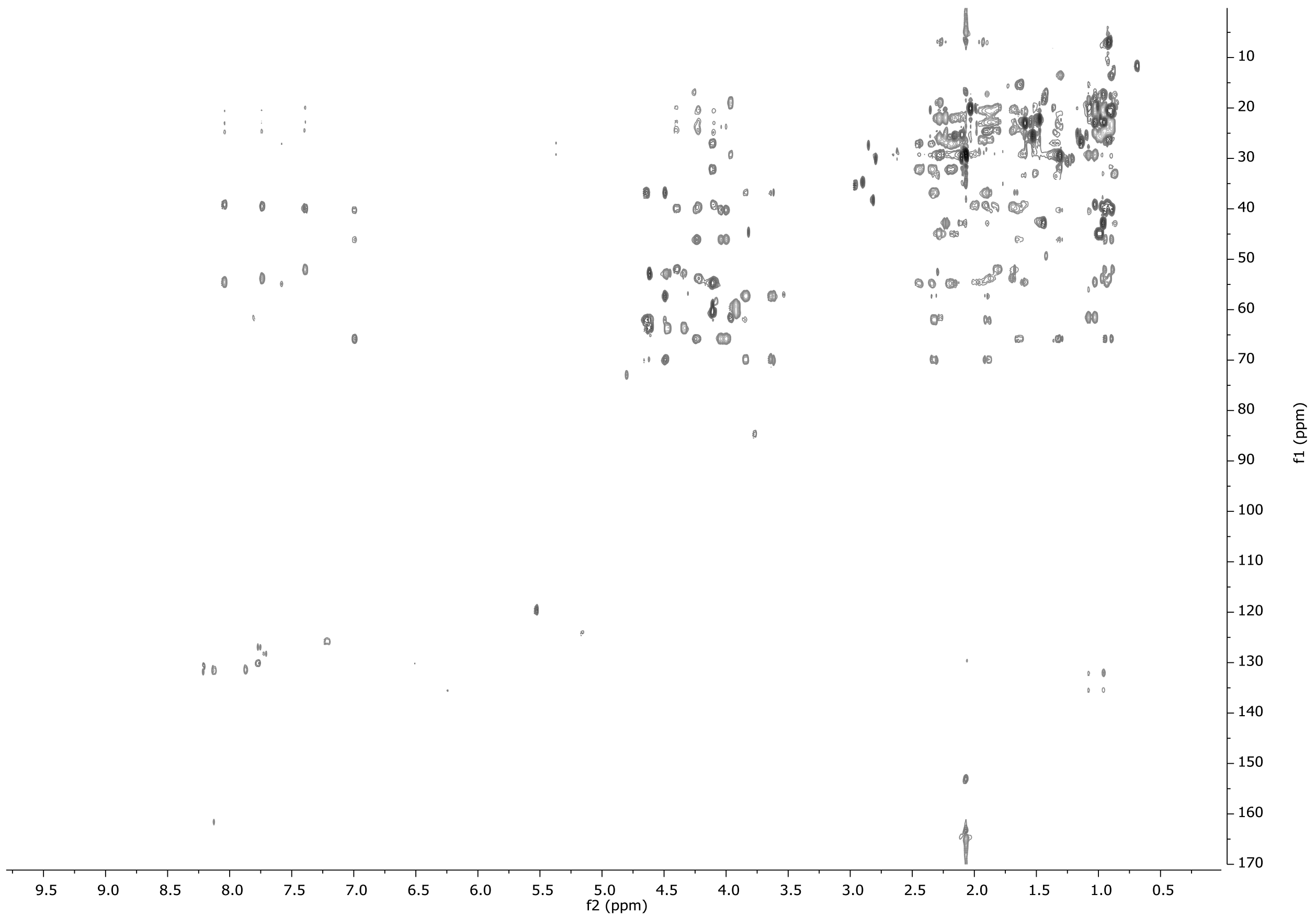

Figure S22. ^{1}H NMR (600 MHz, DMSO-d_6) of roseabol C (**2**)

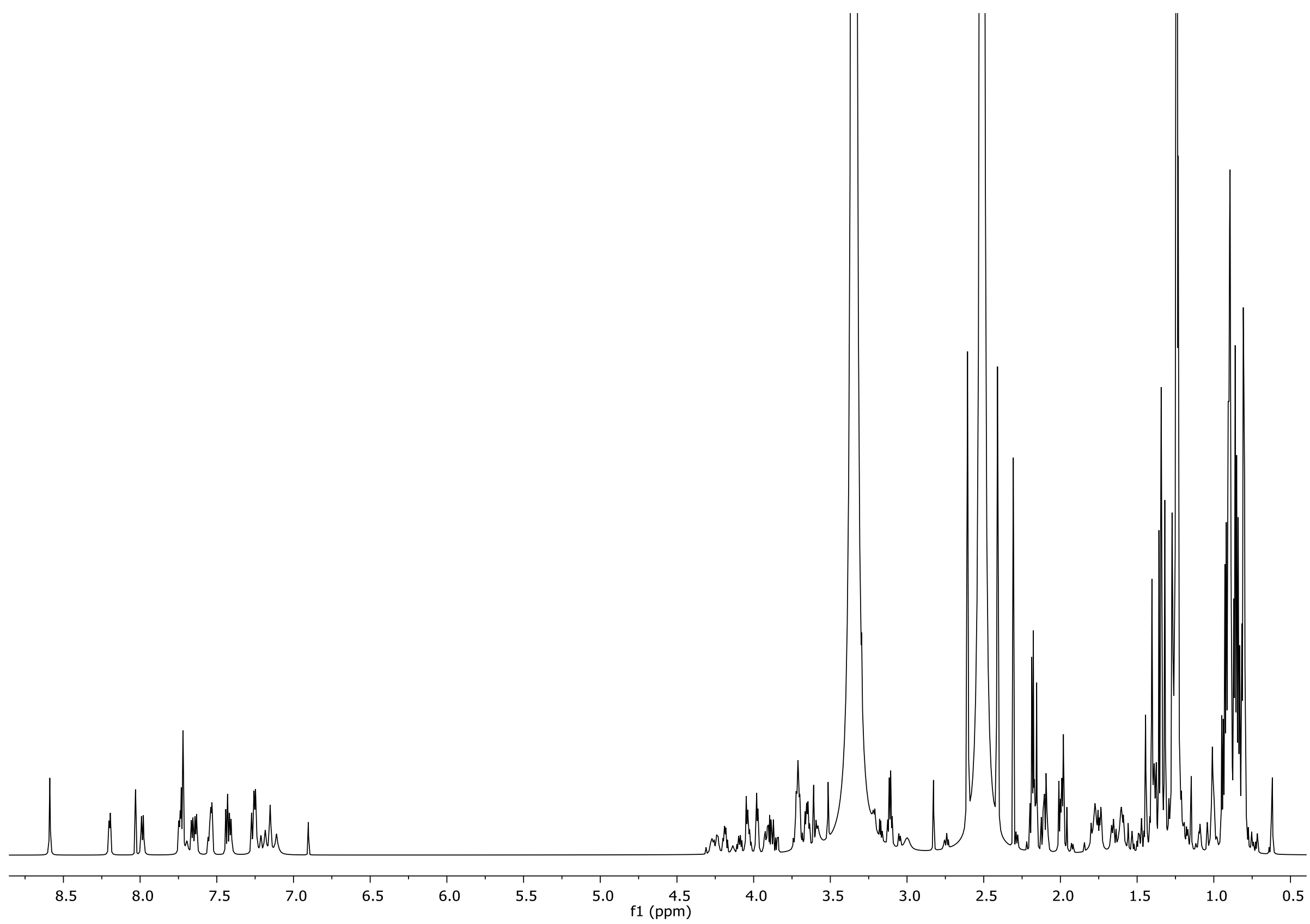

Figure S23. COSY (600 MHz, DMSO-d_6) of roseabol C (**2**)

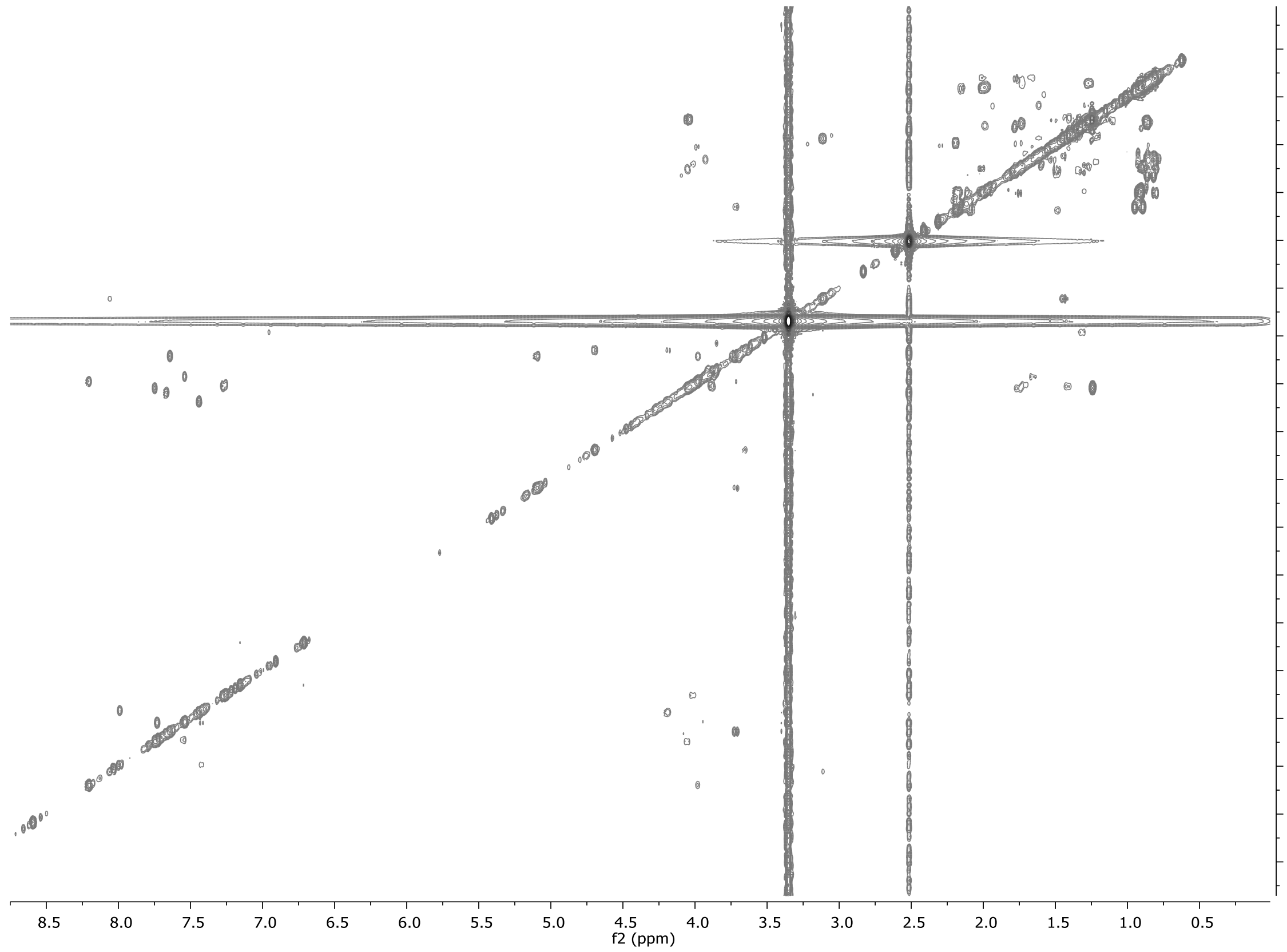

Figure S24. TOSCY (600 MHz, DMSO-d_6) of roseabol C (**2**)

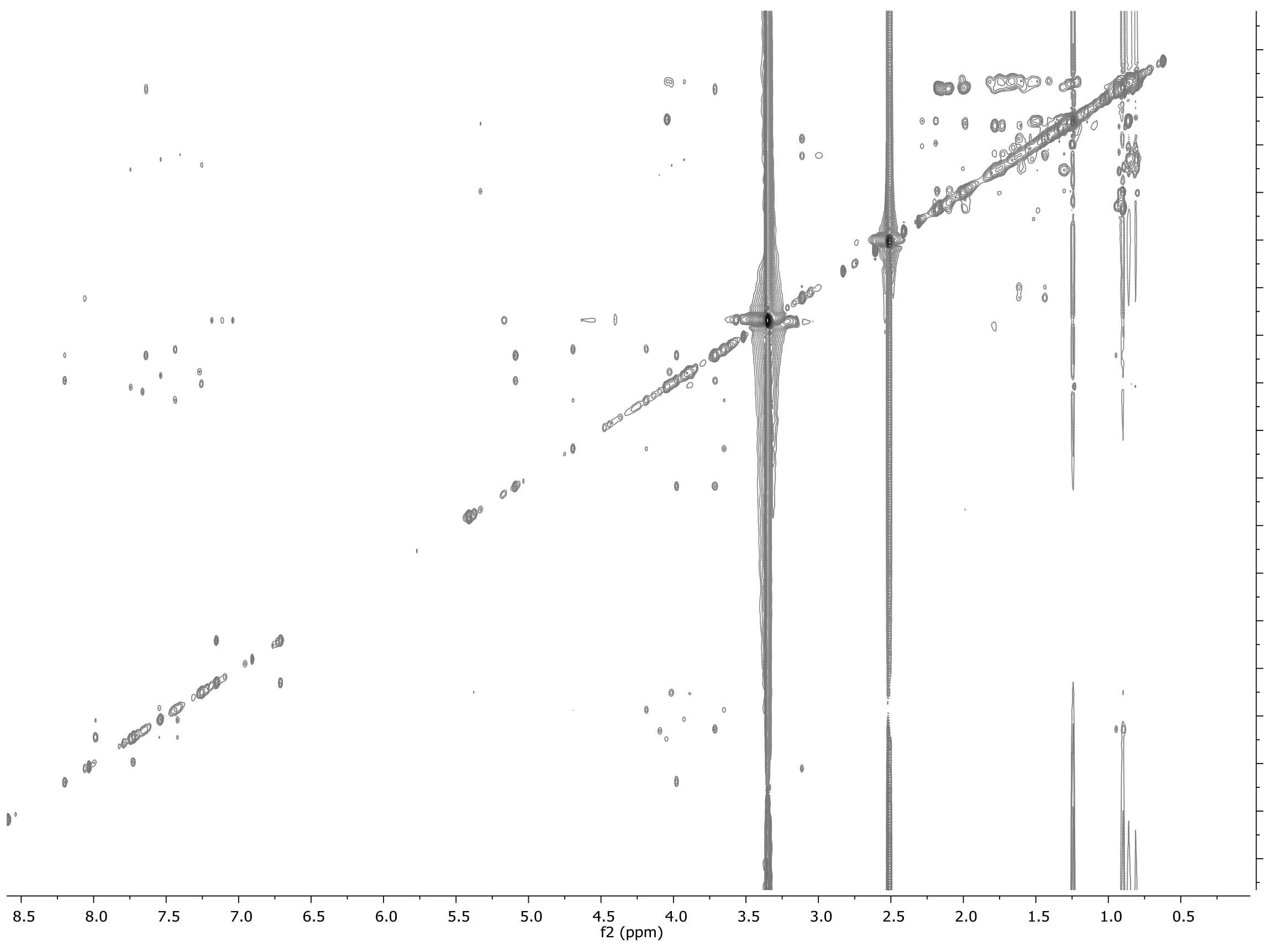

Figure S25. HSQC (600 MHz, DMSO-d6) of roseabol C (**2**)

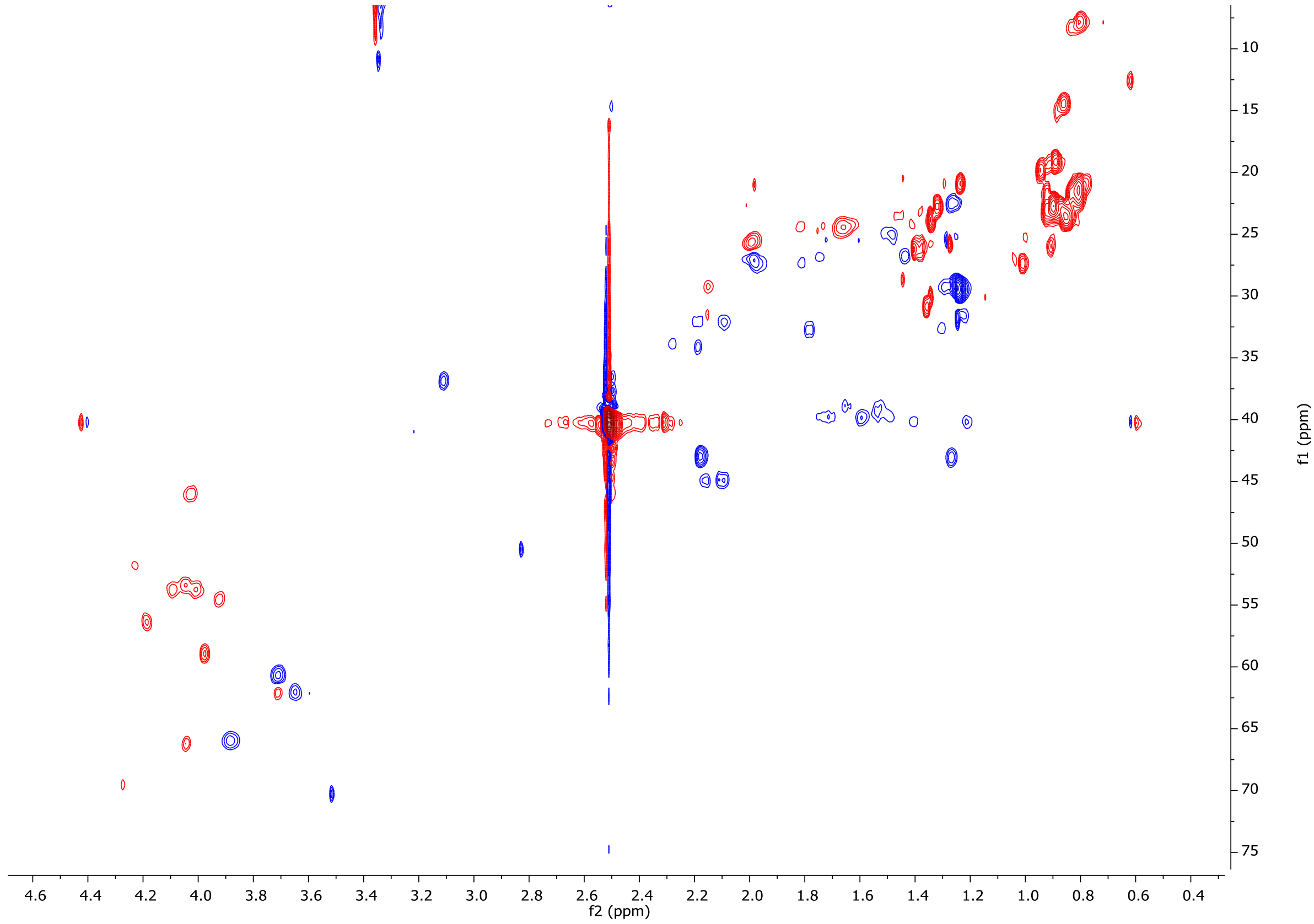

Figure S26. HSQC-TOCY (600 MHz, DMSO-d6) of roseabol C (**2**)

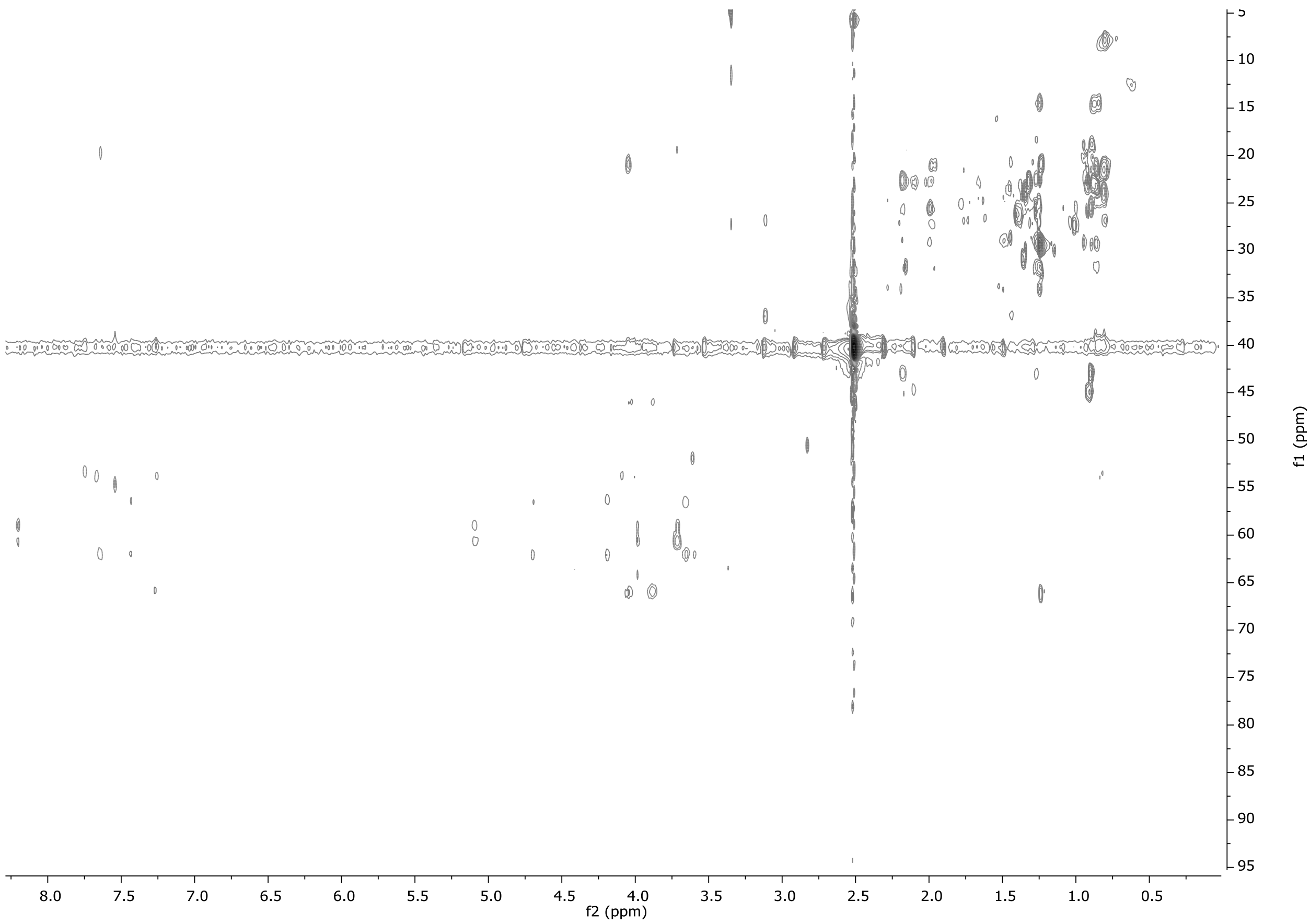

Figure S27. HMBC (600 MHz, DMSO-d_6) of roseabol C (**2**)

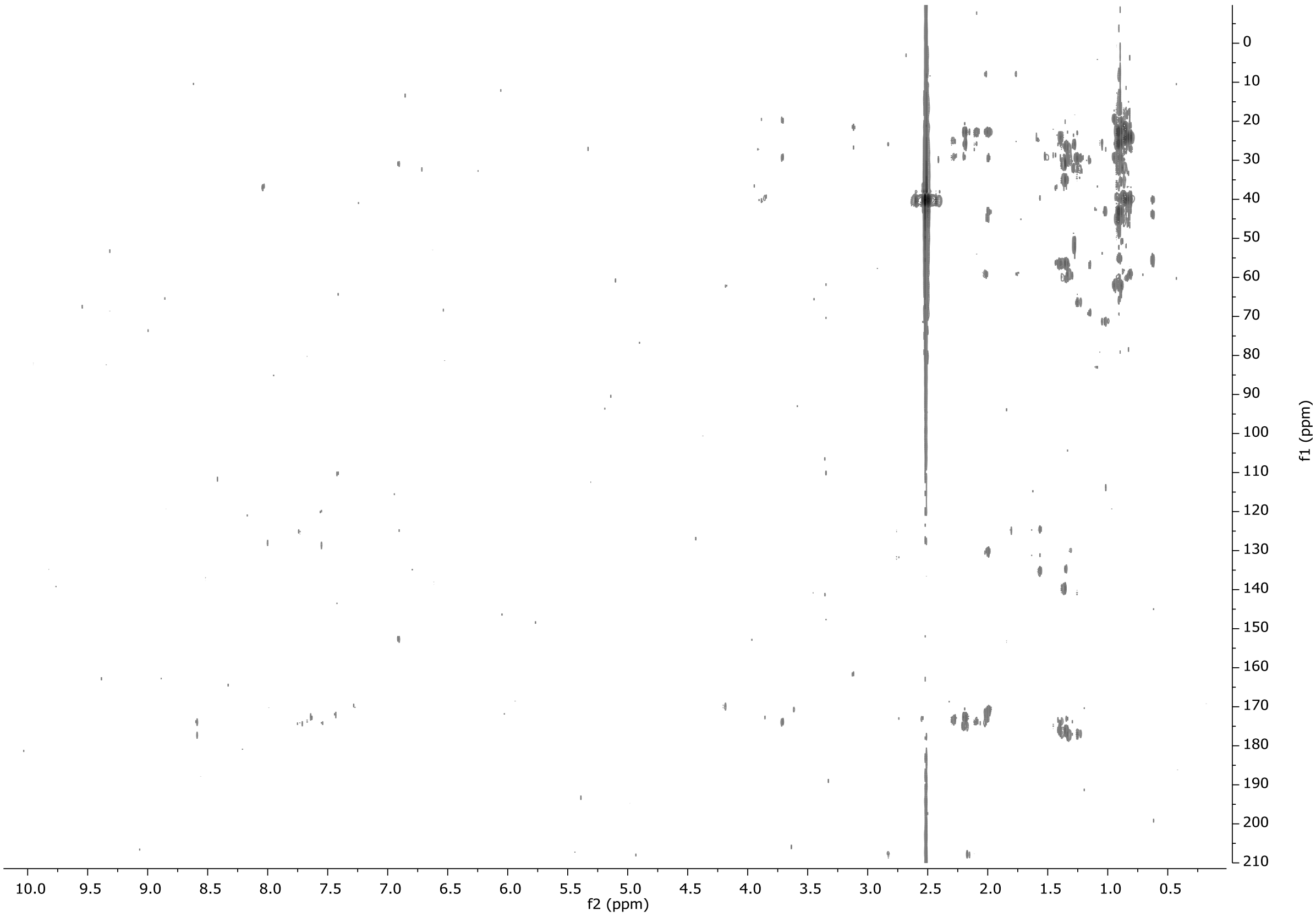

Supplement: Supplementary file 1 [file 13659_2026_659_MOESM1_ESM.pdf]
